# Supplementary material for: GDF15 is a heart‐derived hormone that regulates body growth
Source: EMBO Mol Med. 2017 Jun 1;9(8):1150–64. doi: 10.15252/emmm.201707604 (PMC5538424; doi:10.15252/emmm.201707604)
Supplement: Supplementary file 1 — Appendix [file EMMM-9-1150-s001.pdf]

APPENDIX

GDF15 is a heart-derived hormone that regulates body growth

Ting Wang[1,2,#,ǂ], Jian Liu[1,2,#], Caitlin McDonald[1,2], Katherine Lupino[1,2], Xiandun Zhai[1,2,5], Benjamin J. Wilkins[2], Hakon Hakonarson[3], Liming Pei[1,2,4*]

This appendix includes 4 figures and 5 tables.

Table of Contents **Page**

Appendix Figure S1. FTT and impaired liver GH signaling in αKOγKO mice. 2

Appendix Figure S2. GH concentration in pooled plasma used to treat WT mouse primary hepatocytes. 3

Appendix Figure S3. Impact of GDF15 and BNP on liver GH signaling and body growth. 4

Appendix Figure S4. GDF15 is a major circulating factor in αKOγKO plasma that inhibits hepatocyte GH signaling. 5

Appendix Table S1. Relative plasma protein levels in 16-day-old αHetγWT and αKOγKO mice (n=3 mice per group) measured by SOMAscan. 6

Appendix Table S2. Genes encoding secreted proteins with altered expression in 16-day -old αKOγKO mouse hearts compared to littermate control mouse hearts by RNA-Seq. 46

Appendix Table S3. Sequences of qPCR primers used. 47

Appendix Table S4. Information of human plasma samples used in Fig 6A. 48

Appendix Table S5. Statistical analysis information. 53

Appendix Figure S1

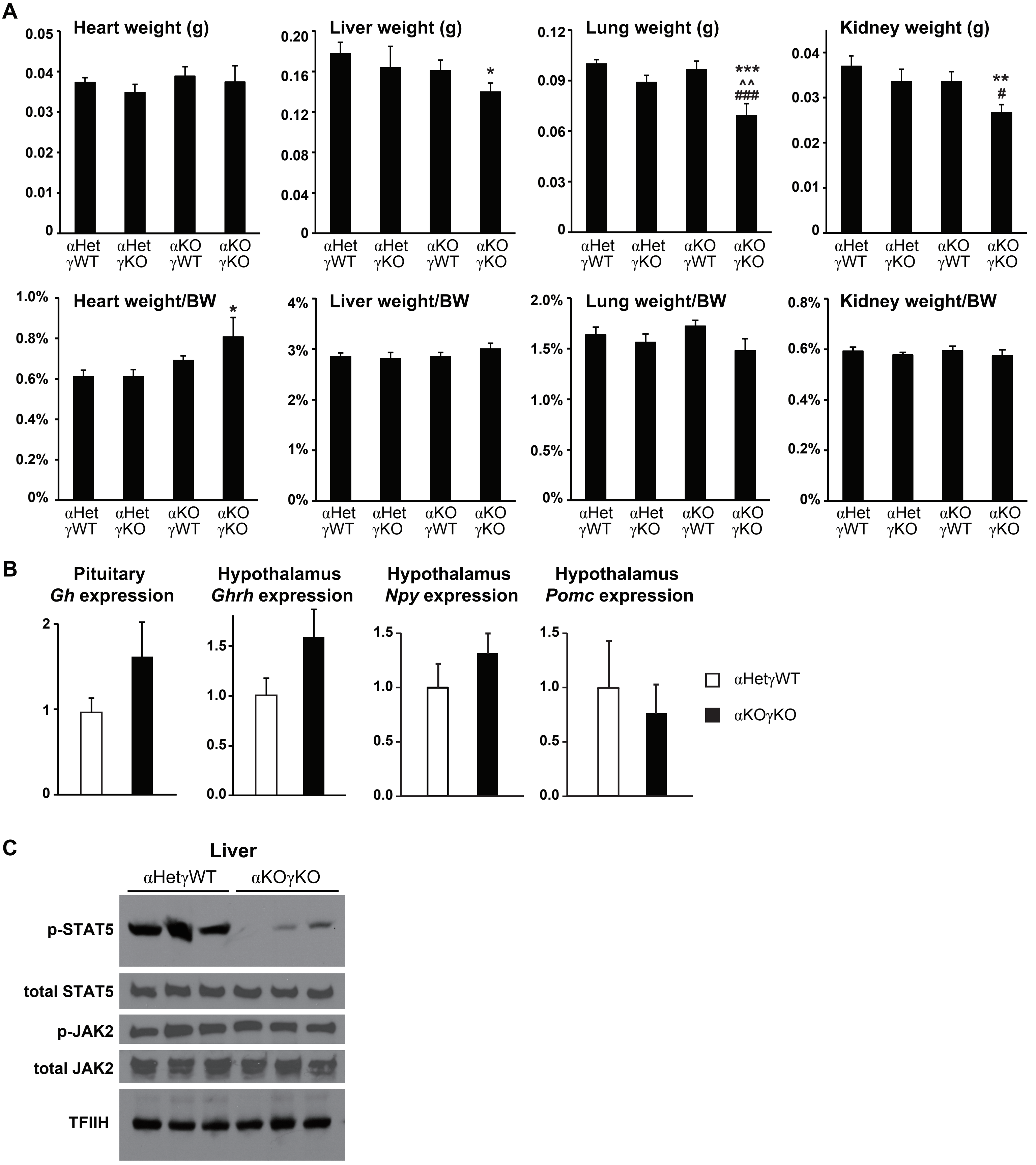

Appendix Figure S1. FTT and impaired liver GH signaling in αKOγKO mice.

A Absolute (top) and relative weight (bottom, normalized to body weight of individual mouse) of different organs in 10-day-old littermate mice (n=6-9 mice per grop). *p<0.05, **p<0.01 and ***p<0.001 between αKOγKO and αHetγWT; ^^p<0.01 between αKOγKO and αHetγKO; $^{\#}$p<0.05 and $^{\#\#\#}$p<0.001 between αKOγKO and αKOγWT by t-test.

B Expression of pituitary *Gh* and hypothalamus *Ghrh*, *Npy* and *Pomc* in 10-day-old littermate mice was determined by qPCR (n=9 mice per group). All values are presented as mean + s.e.m.

C Phosphorylated and total STAT5 and JAK2 levels in 16-day-old littermate mouse livers (n=3 mice per group) were determined by Western blot. TFIIH serves as loading control.

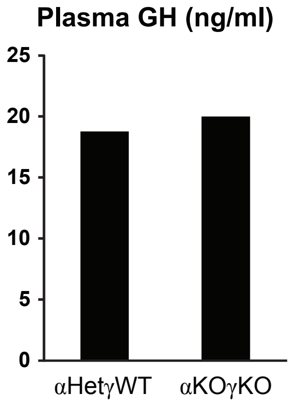

Appendix Figure S2. GH concentration in pooled plasma used to treat WT mouse primary hepatocytes.

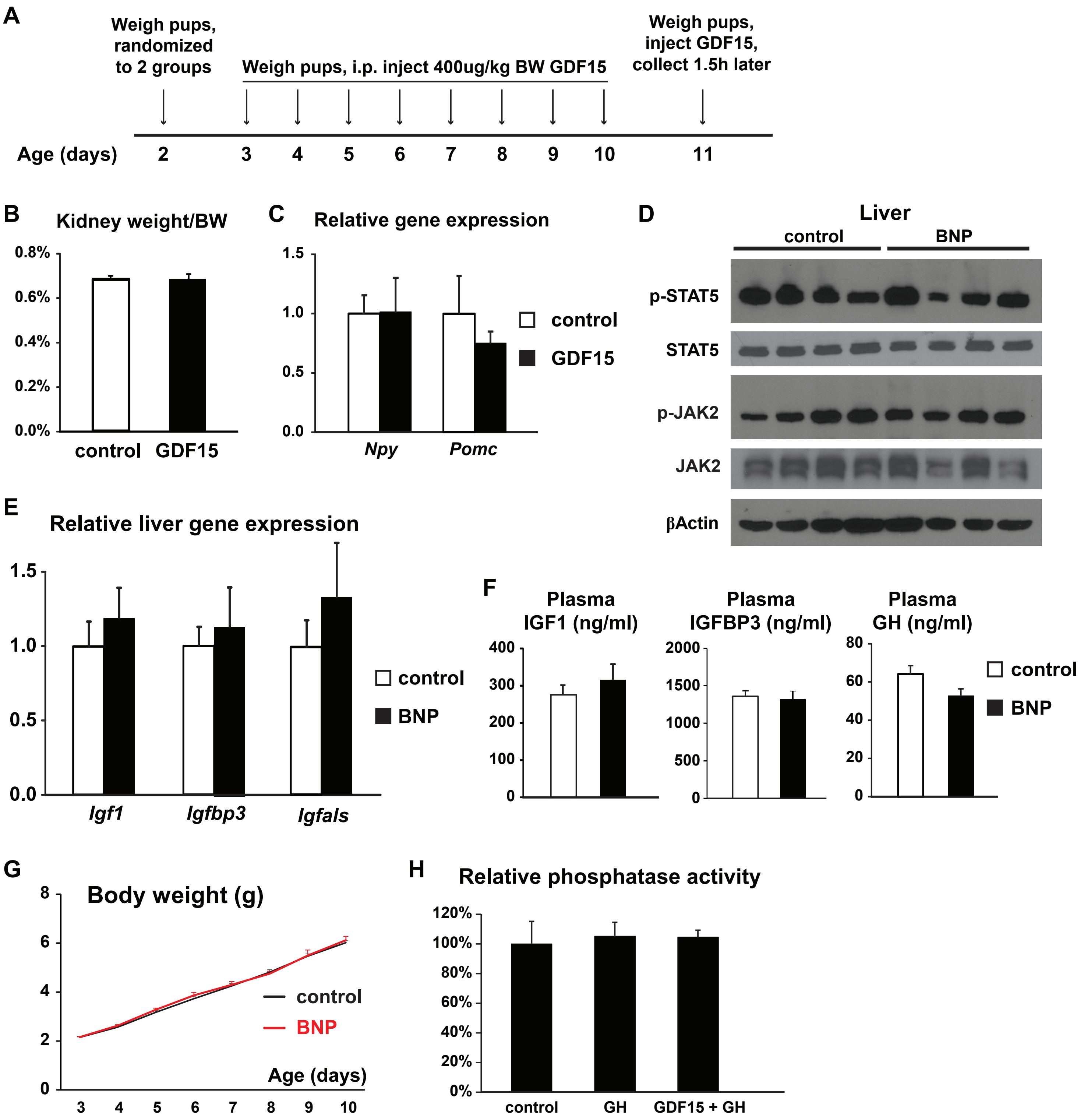

Appendix Figure S3. Impact of GDF15 and BNP on liver GH signaling and body growth.

A Scheme of long term GDF15 treatment in young WT mice. A similar scheme is used for long term BNP treatment in young WT mice.

B Relative kidney weight (normalized to body weight of individual mouse) of weight and gender-matched littermate WT mice injected with control or GDF15 (n=5).

C Expression of hypothalamic *Npy* and *Pomc* in weight and gender-matched littermate WT mice injected with control or GDF15 (n=5).

D-G Liver phosphorylated and total STAT5 and JAK2 levels (D), liver expression of STAT5 target genes *Igf1*, *Igfbp3* and *Igfals* (E), plasma IGF1, IGFBP3 and GH concentrations (F), and body weight (G) in weight and gender-matched littermate WT mice injected with control or BNP (n=4 mice per group, 500 μg/kg BW daily i.p. injection from 3 days of age).

H Overnight-fasted (in DMEM) WT mouse primary hepatocytes (n=2) were first treated with 2 ng/ml GDF15 for 30 minutes, and then with 20 ng/ml GH for 15 minutes. Protein tyrosine phosphotase activities were measured and normalized to total cellular protein amount.

Appendix Figure S4

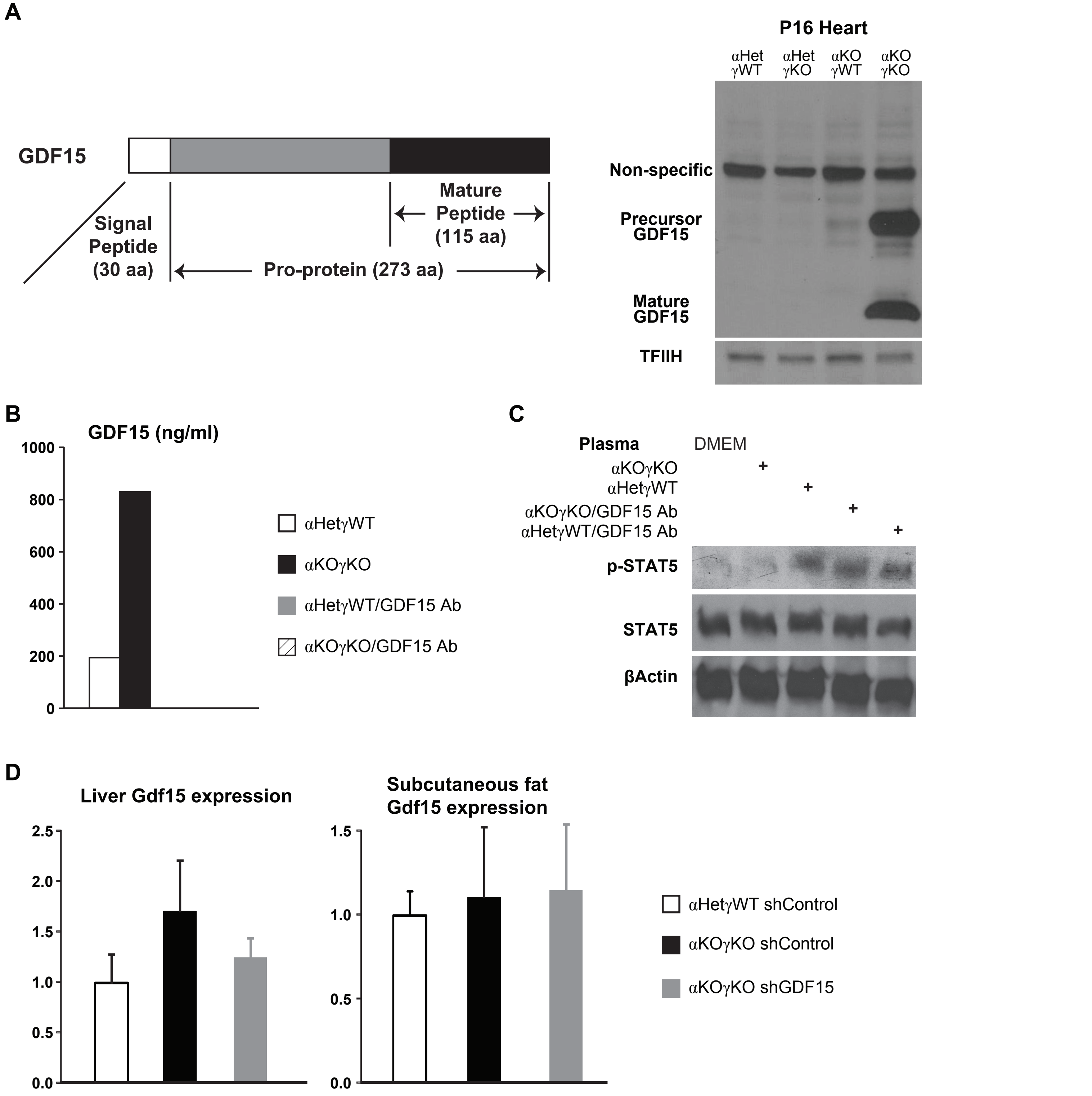

Appendix Figure S4. GDF15 is a major circulating factor in αKOγKO plasma that inhibits hepatocyte GH signaling.

A Left: primary structure of mouse GDF15 protein (based on NP_035949.2). Right: GDF15 protein level in 16-day-old littermate mouse hearts determined by Western blot. TFIIH serves as loading control.

B Plasma GDF15 levels before and after GDF15 antibody incubation were measured by ELISA.

C WT mouse primary hepatocytes were treated for 1 hour with DMEM (control) or mouse plasma pre-incubated with GDF15 antibody. Phosphorylated and total STAT5 were determined by Western blot with βActin as loading control.

D Liver and subcutaneous fat *Gdf15* expression in 9-10 days old littermate control and αKOγKO mice (n=8-12 mice per group) that received pericardial injection of AAV9-control or *Gdf15* shRNA at 2 days of age. Values are mean + s.e.m.

Appendix Table S1. Relative plasma protein levels in 16-day-old αHetγWT and αKOγKO mice (n=3 mice per group) measured by SOMAscan. The proteins are ordered based on fold change (last column). The proteins labeled in blue fonts (end of table) were tested in Fig 3A.

		αHetγWT			αKOγKO			αHetγWT	αKOγKO		Fold (αKOγKO
**TargetFullName**	**EntrezGene**	**#1**	**#2**	**#3**	**#1**	**#2**	**#3**	**Average**	**Average**	**t-test**	**/αHetγWT)**
Insulin-like growth factor I	IGF1	20367.7	16447.2	18374.6	1991.2	1033.9	995.5	18396.5	1340.2	0.002	0.073
Complement C1q subcomponent	C1QA C1QB C1QC	472.0	419.5	474.2	378.7	50.5	49.1	455.2	159.4	0.111	0.350
Insulin-like growth factor-binding protein 5	IGFBP5	107058.8	80090.1	95908.1	60254.3	17965.5	24462.8	94352.3	34227.5	0.025	0.363
Protein kinase B alpha/beta/gamma	AKT1 AKT2 AKT3	9017.7	8718.6	7651.4	7046.2	1055.7	1370.3	8462.6	3157.4	0.107	0.373
Platelet-derived growth factor subunit B	PDGFB	1003.7	1744.4	567.5	397.0	737.0	233.6	1105.2	455.9	0.190	0.412
Creatine kinase M-type:Creatine kinase B-type heterodimer	CKB CKM	6972.1	4723.2	4859.0	1220.9	2600.0	3269.9	5518.1	2363.6	0.030	0.428
Metalloproteinase inhibitor 3	TIMP3	18439.3	24492.2	13119.1	6810.9	13080.1	4347.4	18683.5	8079.5	0.068	0.432
Adenylate kinase isoenzyme 1	AK1	3773.9	9047.6	2458.6	1962.0	1982.3	2786.0	5093.4	2243.4	0.292	0.440
Fractalkine	CX3CL1	431.9	398.3	415.8	385.4	79.1	87.8	415.3	184.1	0.147	0.443
Kallikrein-12	KLK12	1696.5	1502.7	1726.9	1372.7	438.7	405.0	1642.0	738.8	0.098	0.450
DNA repair protein RAD51 homolog 1	RAD51	843.1	744.8	817.2	700.1	193.9	191.0	801.7	361.7	0.118	0.451
Semaphorin-3E	SEMA3E	188.7	159.8	224.2	92.2	80.6	89.2	190.9	87.3	0.027	0.457
Serine/threonine-protein kinase PAK 3	PAK3	611.0	543.6	584.4	525.6	136.8	140.7	579.7	267.7	0.134	0.462
Protein kinase C beta type (splice variant beta-II)	PRKCB	185.4	432.4	204.0	169.6	135.5	85.6	273.9	130.2	0.206	0.475
Angiopoietin-1	ANGPT1	2019.6	2355.6	1394.5	912.0	1140.4	706.2	1923.2	919.5	0.053	0.478
Cathepsin D	CTSD	1228.6	1238.4	1256.4	1164.6	342.5	322.6	1241.1	609.9	0.151	0.491
Interleukin-37	IL37	493.3	391.8	412.1	425.4	110.2	114.2	432.4	216.6	0.167	0.501
Vascular cell adhesion protein 1	VCAM1	2918.5	2556.6	2665.1	2788.4	768.6	655.5	2713.4	1404.2	0.197	0.517
Casein kinase II 2-alpha:2-beta heterotetramer	CSNK2A1 CSNK2B	2185.0	5778.1	5298.0	1695.8	3429.1	1807.0	4420.4	2310.6	0.194	0.523
Fibroblast growth factor 1	FGF1	1719.8	3544.5	1345.0	813.0	818.3	1865.9	2203.1	1165.7	0.268	0.529
Tyrosine-protein kinase ZAP-70	ZAP70	1725.8	1626.3	1740.7	1697.4	499.9	509.9	1697.6	902.4	0.183	0.532
Allograft inflammatory factor 1	AIF1	938.4	1290.3	1326.1	826.2	619.8	475.5	1184.9	640.5	0.029	0.541
Probable G-protein coupled receptor 114	GPR114	217.5	215.2	215.8	192.3	79.1	84.9	216.2	118.8	0.118	0.549
Lymphotoxin alpha2:beta1	LTA LTB	710.0	725.2	823.8	562.0	359.6	325.1	753.0	415.6	0.028	0.552

Tumor necrosis factor receptor superfamily member 11B	TNFRSF11B	4213.5	4039.8	4607.0	3561.5	1887.3	1775.5	4286.8	2408.1	0.073	0.562
Apolipoprotein E	APOE	481.6	596.7	549.8	449.8	257.5	209.0	542.7	305.4	0.066	0.563
High mobility group protein B1	HMGB1	2420.9	4841.1	2904.2	2270.1	1760.2	1707.3	3388.7	1912.5	0.178	0.564
Glutamate carboxypeptidase 2	FOLH1	1091.8	1003.9	1086.2	1022.7	387.6	404.8	1060.6	605.0	0.159	0.570
Cyclin-dependent kinase 5:Cyclin-dependent kinase 5 activator 1 complex	CDK5 CDK5R1	624.6	562.8	603.9	595.0	211.7	223.2	597.1	343.3	0.179	0.575
Corticotropin	POMC	539.9	471.6	779.6	467.8	256.0	309.9	597.0	344.6	0.098	0.577
Pulmonary surfactant-associated protein D	SFTPD	52307.4	82092.6	61360.4	28529.0	43598.7	42521.2	65253.5	38216.3	0.072	0.586
Aspartate aminotransferase, cytoplasmic	GOT1	9515.1	6838.2	7210.2	4459.6	4648.8	4736.2	7854.5	4614.9	0.059	0.588
Junctional adhesion molecule B	JAM2	1056.2	1269.6	1306.5	793.6	703.7	644.0	1210.8	713.8	0.010	0.590
Mast/stem cell growth factor receptor Kit	KIT	265.1	292.3	283.2	243.0	129.8	125.3	280.2	166.0	0.092	0.593
Interleukin-16	IL16	2110.4	2468.2	1978.9	2215.4	781.5	933.8	2185.8	1310.2	0.186	0.599
GTP-binding nuclear protein Ran	RAN	185.9	672.8	434.4	300.5	260.6	214.5	431.0	258.5	0.344	0.600
Fatty acid-binding protein, heart	FABP3	5997.2	11190.9	3333.2	4433.5	3350.1	4614.3	6840.4	4132.6	0.361	0.604
Complement component C8	C8A C8B C8G	1163.3	1083.6	1135.2	1079.3	497.8	480.6	1127.4	685.9	0.152	0.608
Advanced glycosylation end product-specific receptor, soluble	AGER	116.6	92.6	227.2	96.4	82.8	86.7	145.5	88.6	0.303	0.609
Death-associated protein kinase 2	DAPK2	2090.1	3956.5	4529.6	1854.2	3069.6	1535.8	3525.4	2153.2	0.203	0.611
Chordin-like protein 1	CHRDL1	1591.4	1473.0	1503.1	1671.7	482.8	637.2	1522.5	930.6	0.253	0.611
Cofilin-1	CFL1	1693.2	1763.7	1829.1	1525.9	894.8	853.9	1762.0	1091.5	0.087	0.619
Vascular endothelial growth factor receptor 3	FLT4	9553.3	8203.5	8573.0	5188.6	5876.8	5294.3	8776.6	5453.2	0.005	0.621
Low molecular weight phosphotyrosine protein phosphatase	ACP1	588.8	985.2	1036.3	502.0	673.1	447.2	870.1	540.8	0.131	0.621
Interleukin-13 receptor subunit alpha-1	IL13RA1	490.7	429.8	417.8	279.8	259.9	292.2	446.1	277.3	0.009	0.622
Gro-beta/gamma	CXCL3 CXCL2	4833.9	5662.7	4810.7	3113.2	4382.4	2029.6	5102.4	3175.1	0.089	0.622
Complement C3b, inactivated	C3	4759.4	3942.3	4107.1	1640.6	3279.3	3120.9	4269.6	2680.3	0.074	0.628
Follicle stimulating hormone	CGA FSHB	100.7	69.5	98.7	71.2	56.4	42.3	89.6	56.6	0.067	0.632
Carbonic anhydrase-related protein 10	CA10	248.9	217.6	238.3	241.5	100.1	105.3	234.9	149.0	0.201	0.634
Ubiquitin-conjugating enzyme E2 N	UBE2N	11433.4	18023.0	20659.9	9203.8	13578.8	9297.2	16705.4	10693.3	0.147	0.640
Importin subunit beta-1	KPNB1	10500.6	21177.2	18454.6	9694.0	16446.2	6174.7	16710.8	10771.6	0.248	0.645
Coagulation Factor V	F5	36228.4	32792.6	30620.4	19819.5	17323.1	27130.7	33213.8	21424.4	0.037	0.645

Tumor necrosis factor receptor superfamily member 3	LTBR	2008.3	1930.8	2195.6	1726.5	1164.7	1088.5	2044.9	1326.6	0.055	0.649
Stress-induced-phosphoprotein 1	STIP1	11072.7	22296.9	21811.6	9894.9	16575.8	9379.6	18393.7	11950.1	0.224	0.650
Tartrate-resistant acid phosphatase type 5	ACP5	3975.4	3474.6	3963.9	2631.5	2077.7	2794.7	3804.6	2501.3	0.010	0.657
Copine-1	CPNE1	167.1	313.9	286.6	169.1	200.7	135.4	255.9	168.4	0.182	0.658
Biglycan	BGN	4608.8	3740.4	3769.2	3510.8	2428.7	2066.7	4039.5	2668.7	0.067	0.661
Vacuolar protein sorting-associated protein VTA1 homolog	VTA1	1999.7	3679.6	3586.7	1864.0	2665.2	1658.1	3088.7	2062.4	0.195	0.668
Ectodysplasin-A, secreted form	EDA	774.1	704.2	806.6	759.6	382.7	387.5	761.6	509.9	0.176	0.670
Hemoglobin	HBA1 HBB	77662.6	76719.8	85462.7	76318.8	85684.1	134.4	79948.4	54045.8	0.440	0.676
Insulin-like growth factor-binding protein 4	IGFBP4	208.7	213.3	217.7	148.6	145.9	141.1	213.2	145.2	0.000	0.681
Secreted frizzled-related protein 3	FRZB	1397.3	1409.4	1442.7	1370.3	754.0	770.2	1416.5	964.8	0.155	0.681
Killer cell immunoglobulin-like receptor 2DL4	KIR2DL4	382.4	754.5	637.8	335.5	558.3	319.4	591.6	404.4	0.243	0.684
Ephrin type-A receptor 1	EPHA1	1062.9	780.8	780.3	665.9	616.8	512.6	874.7	598.4	0.081	0.684
Desmoglein-1	DSG1	999.3	986.9	1024.7	962.4	544.9	556.2	1003.6	687.8	0.147	0.685
Tropomyosin beta chain	TPM2	1228.4	1004.2	1160.8	1208.5	532.7	591.2	1131.1	777.5	0.239	0.687
Heterogeneous nuclear ribonucleoprotein A/B	HNRNPAB	11107.5	16724.1	14947.5	9448.2	10251.8	9761.4	14259.7	9820.5	0.113	0.689
Connective tissue growth factor	CTGF	724.5	687.2	639.2	438.0	438.8	538.0	683.6	471.6	0.008	0.690
Apoptosis regulator Bcl-2	BCL2	327.0	379.4	374.3	279.5	245.4	221.3	360.2	248.7	0.009	0.690
Transforming growth factor beta-1	TGFB1	816.0	809.0	829.6	708.0	476.3	511.4	818.2	565.2	0.072	0.691
C-C motif chemokine 28	CCL28	301.3	304.2	338.5	286.7	178.4	190.0	314.7	218.4	0.094	0.694
GDNF family receptor alpha-2	GFRA2	4763.1	4227.6	4219.1	3202.9	3335.5	2676.2	4403.3	3071.5	0.008	0.698
Receptor-type tyrosine-protein kinase FLT3	FLT3	876.8	1355.0	1404.8	777.0	1018.7	753.5	1212.2	849.7	0.152	0.701
Phosphatidylinositol 4,5-bisphosphate 3-kinase catalytic subunit gamma isoform	PIK3CG	516.5	525.6	497.8	507.7	295.0	276.8	513.3	359.8	0.173	0.701
Proliferation-associated protein 2G4	PA2G4	6892.6	14550.7	14139.9	7361.2	11707.7	5895.8	11861.1	8321.6	0.316	0.702
Inosine-5'-monophosphate dehydrogenase 1	IMPDH1	4061.4	7371.6	7048.4	3335.5	5908.2	3723.2	6160.5	4322.3	0.242	0.702
Cyclin-dependent kinase 8:Cyclin-C complex	CDK8 CCNC	571.5	564.4	573.1	557.9	322.2	319.4	569.7	399.8	0.165	0.702
Reticulon-4 receptor	RTN4R	2131.8	1738.9	1991.1	1486.8	1145.8	1499.2	1953.9	1377.3	0.024	0.705
Thyroid peroxidase	TPO	860.4	851.8	973.3	753.2	558.5	597.2	895.2	636.3	0.028	0.711
Junctional adhesion molecule C	JAM3	703.4	1118.3	1063.2	560.4	848.8	642.5	961.6	683.9	0.160	0.711

Peptidyl-prolyl cis-trans isomerase D	PPID	2997.9	4964.9	5261.4	2568.0	4466.5	2379.0	4408.1	3137.8	0.262	0.712
NAD-dependent protein deacetylase sirtuin-2	SIRT2	12524.9	21262.8	23076.5	11701.0	17447.1	11488.9	18954.7	13545.7	0.242	0.715
Protein kinase C alpha type	PRKCA	2126.4	4335.7	2317.3	2455.7	2882.6	941.2	2926.5	2093.2	0.418	0.715
Cyclin-dependent kinase 2:Cyclin-A2 complex	CDK2 CCNA2	575.8	803.9	711.1	425.5	598.9	471.5	696.9	498.6	0.082	0.715
40S ribosomal protein S7	RPS7	3169.6	9696.9	5757.9	3896.2	7255.8	2175.5	6208.1	4442.5	0.507	0.716
Inorganic pyrophosphatase	PPA1	11346.6	9789.0	10668.8	7743.4	8035.2	7040.7	10601.5	7606.4	0.008	0.717
beta-adrenergic receptor kinase 1	ADRBK1	847.6	1339.3	955.0	779.9	819.7	661.6	1047.3	753.7	0.180	0.720
Low affinity immunoglobulin epsilon Fc receptor	FCER2	442.6	457.0	459.3	410.3	294.9	273.5	453.0	326.2	0.094	0.720
Heparin cofactor 2	SERPIND1	95764.5	74814.3	87775.9	58372.6	60809.2	67000.0	86118.2	62060.6	0.043	0.721
Drebrin-like protein	DBNL	637.2	1249.4	874.9	639.1	802.0	550.8	920.5	664.0	0.286	0.721
L-Selectin	SELL	337.1	591.9	461.4	305.4	399.7	298.8	463.5	334.6	0.216	0.722
Inhibin beta A chain	INHBA	5163.8	3713.7	3976.2	3748.0	2505.3	3084.7	4284.6	3112.7	0.113	0.726
Ribosomal protein S6 kinase alpha-5	RPS6KA5	37.5	60.5	50.0	29.8	44.7	33.3	49.3	35.9	0.180	0.728
SUMO-conjugating enzyme UBC9	UBE2I	3358.5	4891.9	4686.4	2798.2	3714.6	2957.2	4312.3	3156.7	0.123	0.732
Troponin I, cardiac muscle	TNNI3	5299.9	14526.6	2844.7	5743.2	2784.1	8071.2	7557.1	5532.8	0.641	0.732
Growth/differentiation factor 5	GDF5	359.0	397.8	387.1	285.7	287.7	265.7	381.3	279.7	0.004	0.734
Amphiregulin	AREG	456.2	745.1	707.8	382.6	590.4	427.8	636.4	466.9	0.208	0.734
Cytokine receptor common subunit gamma	IL2RG	548.5	915.9	814.4	452.5	703.3	518.6	759.6	558.1	0.213	0.735
Rab GDP dissociation inhibitor beta	GDI2	40349.5	41216.1	44228.0	29558.4	36189.8	26715.0	41931.2	30821.1	0.043	0.735
Pyridoxal phosphate phosphatase	PDXP	586.2	986.4	936.5	458.7	795.9	590.8	836.4	615.1	0.242	0.735
Tumor necrosis factor receptor superfamily member 13B	TNFRSF13B	243.6	251.0	251.1	220.0	158.7	171.2	248.6	183.3	0.071	0.737
Serine/threonine-protein kinase receptor R3	ACVRL1	337.7	373.9	376.3	294.1	263.8	244.9	362.6	267.6	0.008	0.738
Arginase-1	ARG1	352.7	363.8	352.4	335.6	222.6	230.9	356.3	263.0	0.123	0.738
Cadherin-12	CDH12	200.3	286.2	254.4	166.3	201.8	179.3	247.0	182.5	0.109	0.739
Fibroblast growth factor 16	FGF16	397.7	600.8	544.0	317.1	474.6	351.0	514.2	380.9	0.163	0.741
Catalase	CAT	7708.6	14513.2	17354.0	7615.3	15381.2	6394.7	13191.9	9797.1	0.445	0.743
Complement factor H-related protein 5	CFHR5	221.1	347.2	275.8	197.8	240.1	189.4	281.4	209.1	0.176	0.743
Coagulation Factor X	F10	250.6	237.4	213.8	186.2	153.7	182.4	233.9	174.1	0.016	0.744
Protein jagged-1	JAG1	2485.9	2039.0	2122.1	1785.3	1480.9	1706.4	2215.7	1657.5	0.034	0.748
Complement factor D	CFD	558.4	513.0	559.4	563.7	330.9	326.6	543.6	407.1	0.220	0.749

Superoxide dismutase [Cu-Zn]	SOD1	245.5	365.9	332.2	213.6	272.3	222.2	314.5	236.0	0.147	0.750
Dickkopf-like protein 1	DKKL1	1383.1	2116.0	2060.5	1209.5	1757.5	1207.6	1853.2	1391.5	0.201	0.751
Programmed cell death 1 ligand 2	PDCD1LG2	228.8	361.4	312.0	187.3	290.0	200.8	300.7	226.0	0.214	0.752
Creatine kinase M-type	CKM	2309.6	2173.7	1560.7	1412.2	1471.9	1660.3	2014.7	1514.8	0.153	0.752
Platelet-derived growth factor subunit A	PDGFA	344.6	435.6	367.1	271.3	299.5	292.1	382.4	287.6	0.063	0.752
Interferon gamma	IFNG	237.9	371.3	327.3	204.2	276.6	225.6	312.2	235.5	0.182	0.754
Acid sphingomyelinase-like phosphodiesterase 3a	SMPDL3A	10291.2	14302.3	12579.3	7648.7	10517.8	9899.7	12390.9	9355.4	0.110	0.755
Casein kinase II 2-alpha':2-beta heterotetramer	CSNK2A2 CSNK2B	91.6	191.6	138.5	76.2	156.7	85.8	140.6	106.2	0.423	0.756
Casein kinase II subunit alpha	CSNK2A1	128.1	173.1	171.2	113.4	132.8	112.4	157.5	119.5	0.107	0.759
Glycogen synthase kinase-3 alpha/beta	GSK3A GSK3B	3503.0	5326.7	5697.3	3795.2	3892.7	3344.7	4842.3	3677.5	0.224	0.759
ATP synthase subunit beta, mitochondrial	ATP5B	621.2	627.3	768.5	582.4	521.3	432.1	672.3	511.9	0.070	0.761
Endoplasmic reticulum aminopeptidase 1	ERAP1	185.1	250.5	272.9	193.4	174.3	173.9	236.2	180.5	0.163	0.764
Prostaglandin G/H synthase 2	PTGS2	250.0	425.2	281.0	234.9	251.1	245.2	318.7	243.7	0.299	0.765
Prefoldin subunit 5	PFDN5	522.6	689.1	648.6	556.7	496.4	373.1	620.1	475.4	0.122	0.767
CD27 antigen	CD27	31173.8	25967.0	28832.9	16978.2	20783.7	28151.6	28657.9	21971.2	0.167	0.767
Delta-like protein 1	DLL1	2848.3	2392.4	2445.0	2076.7	1753.7	2067.8	2561.9	1966.1	0.033	0.767
Interleukin-23 receptor	IL23R	226.7	333.2	320.3	195.8	270.2	210.0	293.4	225.3	0.178	0.768
Interleukin-22	IL22	204.7	268.7	263.0	167.0	212.3	186.4	245.5	188.6	0.091	0.768
Platelet-activating factor acetylhydrolase IB subunit beta	PAFAH1B2	833.5	1122.4	1288.2	712.0	978.1	805.7	1081.4	831.9	0.197	0.769
Dickkopf-related protein 4	DKK4	2035.0	1896.3	2104.7	1638.9	1339.8	1666.3	2012.0	1548.3	0.028	0.770
Ubiquitin-conjugating enzyme E2 L3	UBE2L3	391.4	601.5	580.0	319.3	494.5	397.2	524.3	403.7	0.228	0.770
Desmoglein-2	DSG2	478.5	401.3	1882.6	375.0	353.2	1400.8	920.8	709.7	0.741	0.771
Protein 4.1	EPB41	2432.6	6177.9	4298.9	3550.9	4353.7	2073.4	4303.1	3326.0	0.493	0.773
Tyrosine-protein kinase Yes	YES1	567.2	728.2	767.7	522.9	605.1	466.8	687.7	531.6	0.111	0.773
Tyrosine-protein phosphatase non-receptor type 1	PTPN1	2180.8	2167.1	2198.7	2056.4	1493.3	1516.0	2182.2	1688.6	0.115	0.774
Calcineurin subunit B type 1	PPP3R1	4698.0	6486.1	6715.4	3661.5	5815.8	4403.4	5966.5	4626.9	0.210	0.775
DNA topoisomerase 1	TOP1	224.0	294.7	293.8	204.1	238.2	189.2	270.8	210.5	0.107	0.777
Tyrosine-protein kinase CSK	CSK	2982.9	4870.6	3561.9	2928.6	3792.1	2155.2	3805.1	2958.6	0.313	0.778
Endothelin-converting enzyme 1	ECE1	442.0	616.4	584.4	368.7	499.1	410.7	547.6	426.2	0.147	0.778
UMP-CMP kinase	CMPK1	2206.4	2783.7	3416.0	1734.6	2521.9	2286.8	2802.0	2181.1	0.223	0.778

Immunoglobulin G	IGHG1 IGHG2 IGHG3 IGHG4 IGK IGL	420.5	678.4	608.0	354.8	550.8	423.3	569.0	443.0	0.265	0.779
Nascent polypeptide-associated complex subunit alpha	NACA	4825.7	7647.9	8541.6	4691.5	7220.7	4477.4	7005.1	5463.2	0.343	0.780
Interleukin-1 receptor-like 2	IL1RL2	226.8	241.9	241.5	194.0	182.8	178.9	236.7	185.2	0.002	0.782
Kremen protein 2	KREMEN2	684.7	695.4	718.6	661.5	492.3	488.4	699.6	547.4	0.112	0.782
Apolipoprotein E (isoform E2)	APOE	300.1	425.7	350.8	257.2	305.7	281.2	358.9	281.4	0.156	0.784
cGMP-dependent 3',5'-cyclic phosphodiesterase	PDE2A	5359.4	7378.0	7542.4	4441.2	6685.2	4812.1	6759.9	5312.8	0.217	0.786
Histone deacetylase 8	HDAC8	723.4	740.7	773.6	736.3	511.9	513.2	745.9	587.1	0.163	0.787
1-phosphatidylinositol 4,5-bisphosphate phosphodiesterase gamma-1	PLCG1	319.0	245.9	306.5	253.9	214.9	217.5	290.5	228.8	0.093	0.788
NSFL1 cofactor p47	NSFL1C	353.0	508.6	420.5	348.4	418.9	244.6	427.4	337.3	0.255	0.789
Peroxiredoxin-6	PRDX6	1556.5	1516.6	1772.9	1249.8	1696.8	881.3	1615.3	1276.0	0.284	0.790
Interleukin-17F	IL17F	72.5	71.9	77.2	70.4	49.7	55.1	73.9	58.4	0.122	0.791
Luteinizing hormone	CGA LHB	215.4	338.5	300.2	191.9	268.4	215.9	284.7	225.4	0.251	0.792
Kallikrein-6	KLK6	1902.4	1662.5	1670.0	1455.8	1475.9	1213.2	1745.0	1381.6	0.035	0.792
40S ribosomal protein SA	RPSA	1533.8	1306.6	1415.6	1068.4	1194.0	1114.8	1418.7	1125.7	0.028	0.794
Troponin I, fast skeletal muscle	TNNI2	2539.6	2074.2	2472.6	2687.7	1262.7	1672.8	2362.1	1874.4	0.371	0.794
C-C motif chemokine 1	CCL1	1281.9	1089.4	1233.6	1236.1	795.5	832.1	1201.6	954.6	0.215	0.794
Tyrosine-protein kinase JAK2	JAK2	714.0	602.6	595.2	479.1	468.7	572.3	637.3	506.7	0.063	0.795
Matrix metalloproteinase-17	MMP17	255.5	263.4	244.5	204.6	209.3	193.6	254.5	202.5	0.002	0.796
NKG2D ligand 3	ULBP3	102.3	144.1	139.2	86.9	121.6	98.5	128.5	102.3	0.196	0.796
Coagulation factor IX	F9	126541.9	91636.1	115667.0	88241.0	79804.8	97772.4	111281.7	88606.1	0.146	0.796
Low-density lipoprotein receptor-related protein 8	LRP8	301.6	305.7	325.8	253.4	240.5	249.6	311.0	247.8	0.005	0.797
C-C motif chemokine 5	CCL5	261.9	353.2	328.6	217.0	285.0	250.2	314.6	250.7	0.137	0.797
Methionine aminopeptidase 2	METAP2	7391.9	18779.2	12962.2	10975.6	12010.2	8262.9	13044.4	10416.2	0.515	0.799
Dermatopontin	DPT	4339.3	3483.4	3549.4	3489.2	2395.9	3204.2	3790.7	3029.8	0.152	0.799
Oncostatin-M	OSM	777.6	712.1	1017.2	732.6	573.0	699.1	835.6	668.2	0.207	0.800
Adiponectin	ADIPOQ	59.7	76.0	64.5	45.9	59.6	54.6	66.7	53.4	0.103	0.800
Laminin	LAMA1 LAMB1 LAMC1	84.1	107.9	100.1	67.5	90.3	76.6	97.4	78.1	0.117	0.802
Fibroblast growth factor 8 isoform A	FGF8	69.4	101.3	94.4	63.3	83.1	66.4	88.4	70.9	0.216	0.803
Dickkopf-related protein 1	DKK1	1604.2	1540.2	1766.3	1316.1	1149.7	1482.1	1636.9	1316.0	0.059	0.804

Histone-lysine N-methyltransferase EHMT2	EHMT2	1773.6	1715.4	1913.3	1116.8	1401.3	1827.5	1800.8	1448.5	0.225	0.804
Creatine kinase B-type	CKB	1297.5	829.3	1248.5	939.7	790.3	987.8	1125.1	905.9	0.276	0.805
Mitogen-activated protein kinase 1	MAPK1	560.8	771.4	660.5	513.4	567.6	525.6	664.2	535.5	0.161	0.806
Vascular endothelial growth factor receptor 2	KDR	790.8	1179.5	1168.9	715.7	1066.8	750.3	1046.4	844.3	0.301	0.807
AH receptor-interacting protein	AIP	352.1	474.4	459.5	317.6	377.0	343.0	428.7	345.9	0.152	0.807
Transforming growth factor-beta-induced protein ig-h3	TGFBI	38395.9	30011.2	38790.0	32550.9	20924.4	33073.6	35732.4	28849.6	0.239	0.807
Vitamin K-dependent protein C	PROC	149.3	205.4	180.9	140.3	154.6	137.7	178.5	144.2	0.160	0.808
Calcium/calmodulin-dependent protein kinase kinase 1	CAMKK1	212.3	326.1	291.0	192.0	266.5	212.1	276.5	223.5	0.270	0.809
Calcium/calmodulin-dependent protein kinase type II subunit beta	CAMK2B	505.9	732.5	445.1	471.2	447.5	444.0	561.2	454.2	0.346	0.809
Membrane frizzled-related protein	MFRP	101823.6	93145.0	79481.5	75435.7	78689.1	68222.9	91483.4	74115.9	0.099	0.810
Fibroblast growth factor receptor 2	FGFR2	268.7	77.4	356.6	191.1	74.9	303.4	234.2	189.8	0.696	0.810
3-hydroxy-3-methylglutaryl-coenzyme A reductase	HMGCR	148.5	162.9	149.0	141.5	111.9	119.7	153.5	124.4	0.061	0.810
Neurexophilin-1	NXPH1	1291.4	1424.1	1119.1	1108.6	1107.9	895.9	1278.2	1037.5	0.104	0.812
Alcohol dehydrogenase [NADP(+)]	AKR1A1	38392.8	28180.0	30431.5	25927.6	29308.4	23594.2	32334.8	26276.7	0.181	0.813
Inhibitor of growth protein 1	ING1	842.2	1126.9	1118.7	677.5	930.4	902.4	1029.3	836.8	0.195	0.813
Extracellular matrix protein 1	ECM1	226.1	223.4	231.4	235.9	162.2	155.9	227.0	184.7	0.241	0.814
Tumor necrosis factor ligand superfamily member 11	TNFSF11	197.4	222.7	239.0	179.8	191.2	165.5	219.7	178.8	0.056	0.814
Cyclin-dependent kinase inhibitor 1B	CDKN1B	290.4	414.4	393.1	294.9	308.8	290.2	366.0	298.0	0.216	0.814
C-X-C motif chemokine 5	CXCL5	274.6	291.3	319.2	246.6	240.4	233.8	295.0	240.3	0.043	0.814
SLIT and NTRK-like protein 5	SLITRK5	2088.8	1373.8	1574.7	1539.6	1114.2	1449.8	1679.1	1367.9	0.293	0.815
Ubiquitin	RPS27A	57186.6	70672.6	86911.8	58837.3	65149.3	51144.9	71590.3	58377.2	0.263	0.815
Seizure 6-like protein 2	SEZ6L2	350.4	501.1	471.7	287.5	423.4	368.8	441.1	359.9	0.254	0.816
Kinesin-like protein KIF23	KIF23	151.6	234.3	204.8	148.8	184.4	148.9	196.9	160.7	0.274	0.816
Malate dehydrogenase, cytoplasmic	MDH1	138773.9	136748.8	100111.6	92786.3	106056.0	107943.8	125211.4	102262.0	0.201	0.817
Beta-endorphin	POMC	148.0	140.0	182.4	145.1	113.1	126.0	156.8	128.1	0.154	0.817
Neuronal cell adhesion molecule	NRCAM	1036.5	821.9	944.6	760.7	688.1	841.0	934.3	763.3	0.096	0.817
Calcium/calmodulin-dependent protein kinase type II subunit delta	CAMK2D	1495.9	1889.6	1267.0	1309.2	1266.6	1225.3	1550.8	1267.0	0.258	0.817
Protein disulfide-isomerase A3	PDIA3	705.4	987.9	995.7	612.8	890.4	693.9	896.3	732.4	0.265	0.817

Opioid-binding protein/cell adhesion molecule	OPCML	1859.8	1685.5	1712.2	1586.6	1535.2	1175.9	1752.5	1432.6	0.117	0.817
SHC-transforming protein 1	SHC1	2972.5	3943.6	4153.6	2950.5	3589.6	2519.4	3689.9	3019.8	0.236	0.818
Interleukin-2 receptor subunit alpha	IL2RA	1223.1	362.7	379.3	953.5	319.3	337.1	655.0	536.6	0.755	0.819
Decorin	DCN	186.3	259.6	234.6	167.3	214.3	176.0	226.8	185.9	0.199	0.819
Bone morphogenetic protein 1	BMP1	9811.2	7872.6	8696.7	6776.5	6628.3	8212.3	8793.5	7205.7	0.104	0.819
Peptidyl-prolyl cis-trans isomerase E	PPIE	244.3	343.7	326.7	241.0	272.4	237.3	304.9	250.2	0.210	0.821
Kallikrein-14	KLK14	2327.6	3316.6	3199.5	1992.8	3026.5	2242.6	2947.9	2420.6	0.298	0.821
Calcium/calmodulin-dependent protein kinase type 1D	CAMK1D	661.3	624.0	677.5	623.5	484.7	503.7	654.3	537.3	0.101	0.821
Hyaluronan and proteoglycan link protein 1	HAPLN1	24298.0	18879.8	21487.2	17632.5	16137.4	19334.8	21555.0	17701.6	0.117	0.821
Complement component C6	C6	492.5	549.8	523.6	385.2	449.8	453.2	522.0	429.4	0.032	0.823
Eukaryotic translation initiation factor 5A-1	EIF5A	11847.4	24206.0	21496.3	15059.0	20667.2	11695.4	19183.2	15807.2	0.506	0.824
Ephrin type-B receptor 6	EPHB6	12137.9	8943.9	11473.0	9973.8	7448.5	9416.0	10851.6	8946.1	0.203	0.824
Macrophage scavenger receptor types I and II	MSR1	319.1	368.2	390.0	242.6	311.0	336.3	359.1	296.6	0.154	0.826
DnaJ homolog subfamily B member 1	DNAJB1	116.1	230.2	210.2	138.7	200.9	120.4	185.5	153.3	0.499	0.827
Mannose-binding protein C	MBL2	217.1	302.4	285.6	179.1	248.1	238.8	268.4	222.0	0.245	0.827
Retinol-binding protein 4	RBP4	344.1	535.1	413.9	362.1	402.5	308.8	431.0	357.8	0.326	0.830
Serine/threonine-protein kinase 17B	STK17B	1511.6	1950.7	2170.9	1316.6	1799.1	1562.5	1877.7	1559.4	0.260	0.830
Growth-regulated alpha protein	CXCL1	394.4	427.5	453.3	341.1	366.5	351.5	425.1	353.0	0.036	0.831
Retinoic acid receptor responder protein 2	RARRES2	84182.5	68765.5	76084.7	66721.4	51617.8	72024.0	76344.2	63454.4	0.170	0.831
Heterogeneous nuclear ribonucleoproteins A2/B1	HNRNPA2B1	36132.1	40899.8	39119.4	29582.4	32715.0	34408.9	38717.1	32235.4	0.031	0.833
Glycylpeptide N-tetradecanoyltransferase 1	NMT1	671.2	960.9	1033.2	637.7	850.8	730.7	888.4	739.7	0.322	0.833
Complement decay-accelerating factor	CD55	118.7	165.2	140.8	114.6	125.3	113.9	141.6	117.9	0.215	0.833
Elongation factor 1-beta	EEF1B2	435.0	629.9	566.2	370.1	574.1	415.1	543.7	453.1	0.344	0.833
Interleukin-5	IL5	3644.4	4404.3	5073.7	2830.7	4000.6	4108.8	4374.1	3646.7	0.279	0.834
Erythropoietin receptor	EPOR	92187.3	91829.6	75512.8	74672.7	74288.9	67617.5	86509.9	72193.0	0.106	0.835
Serine/threonine-protein kinase 16	STK16	496.7	492.1	487.9	462.4	378.0	393.5	492.2	411.3	0.088	0.836
Prolactin	PRL	1020.9	1306.6	1205.9	845.8	1087.0	1024.4	1177.8	985.7	0.159	0.837

Tumor necrosis factor receptor superfamily member 25	TNFRSF25	125.8	92.6	321.5	99.0	77.4	275.6	180.0	150.7	0.774	0.837
C-C motif chemokine 23	CCL23	89.1	84.3	84.1	81.8	65.6	68.5	85.8	72.0	0.097	0.838
C-type lectin domain family 7 member A	CLEC7A	546.0	612.9	683.1	439.3	537.2	568.1	614.0	514.9	0.148	0.839
Carboxypeptidase E	CPE	2267.7	1935.4	2612.5	2111.1	1550.1	2055.5	2271.9	1905.6	0.239	0.839
Dual specificity tyrosine-phosphorylation-regulated kinase 3	DYRK3	572.8	705.6	818.7	490.1	673.7	597.2	699.0	587.0	0.281	0.840
Ras GTPase-activating protein 1	RASA1	581.4	506.8	531.2	587.4	373.3	399.8	539.8	453.5	0.329	0.840
Limbic system-associated membrane protein	LSAMP	12129.8	9974.3	9862.7	11140.8	7820.8	7894.8	10655.6	8952.1	0.275	0.840
Fibroblast growth factor 7	FGF7	280.0	343.1	315.4	241.4	293.7	253.4	312.8	262.8	0.109	0.840
Angiotensin-converting enzyme 2	ACE2	109.2	134.0	138.0	89.6	114.1	116.9	127.1	106.9	0.182	0.841
Collectin-12	COLEC12	703.6	840.7	848.9	571.7	715.6	727.0	797.7	671.4	0.140	0.842
C-C motif chemokine 3-like 1	CCL3L1	649.5	639.8	685.1	589.8	535.2	538.1	658.1	554.4	0.011	0.842
Proteasome subunit alpha type-6	PSMA6	177.0	253.3	244.4	171.1	219.0	178.6	224.9	189.6	0.293	0.843
Hepatitis A virus cellular receptor 2	HAVCR2	180.4	224.0	205.0	153.0	184.2	176.7	203.1	171.3	0.119	0.843
Peroxiredoxin-5, mitochondrial	PRDX5	786.8	676.1	490.1	630.2	521.9	495.3	651.0	549.1	0.369	0.844
Angiopoietin-related protein 4	ANGPTL4	6688.6	7080.8	8585.3	5102.4	6392.0	7393.2	7451.6	6295.9	0.260	0.845
Testican-2	SPOCK2	1636.7	1083.0	1740.2	1641.2	1072.5	1056.7	1486.6	1256.8	0.458	0.845
Ephrin type-B receptor 2	EPHB2	7272.5	5390.0	6872.1	5963.3	4923.2	5635.7	6511.5	5507.4	0.218	0.846
Basal Cell Adhesion Molecule	BCAM	173.5	148.5	154.4	151.9	120.5	130.7	158.8	134.4	0.113	0.846
Fatty acid-binding protein, epidermal	FABP5	1439.2	690.2	443.7	566.6	943.9	667.0	857.7	725.8	0.712	0.846
Inosine-5'-monophosphate dehydrogenase 2	IMPDH2	3015.2	3985.4	4013.9	2611.9	3706.3	3006.2	3671.5	3108.1	0.286	0.847
Azurocidin	AZU1	95.1	125.7	116.4	85.2	107.6	92.7	112.4	95.2	0.205	0.847
CD97 antigen	CD97	890.5	807.5	865.9	851.4	657.0	664.0	854.6	724.1	0.166	0.847
Basigin	BSG	11615.1	14121.7	14936.0	9436.6	13387.9	11638.7	13557.6	11487.7	0.246	0.847
Tyrosine-protein kinase BTK	BTK	1978.7	3970.4	2406.6	2400.3	3185.5	1509.3	2785.2	2365.0	0.618	0.849
Cadherin-3	CDH3	6937.5	6253.1	6804.9	5415.9	5083.8	6479.4	6665.2	5659.7	0.124	0.849
Mitogen-activated protein kinase kinase kinase 7:TGF-beta-activated kinase 1 and MAP3K7-binding protein 1 fusion	MAP3K7 TAB1	1341.7	1601.1	1900.7	1098.5	1533.6	1481.3	1614.5	1371.1	0.316	0.849
Serine protease 27	PRSS27	1750.5	2145.1	2491.7	1505.8	2028.0	1893.9	2129.1	1809.2	0.300	0.850
Leucine-rich repeat transmembrane protein FLRT1	FLRT1	203.0	239.5	220.4	178.0	203.1	182.3	221.0	187.8	0.070	0.850

Stabilin-2	STAB2	407.3	421.9	463.2	375.8	380.3	343.1	430.8	366.4	0.040	0.851
Alpha-2-HS-glycoprotein	AHSG	391.2	395.0	405.2	303.7	370.0	341.4	397.1	338.4	0.086	0.852
Hsp90 co-chaperone Cdc37	CDC37	123.3	148.1	141.9	103.5	121.9	126.9	137.8	117.4	0.120	0.852
Fc receptor-like protein 3	FCRL3	776.3	160.0	167.2	595.3	167.8	178.2	367.8	313.8	0.839	0.853
Bone morphogenetic protein receptor type-1A	BMPR1A	209.8	151.8	210.4	181.3	122.4	184.7	190.7	162.8	0.377	0.854
Bcl-2-like protein 1	BCL2L1	201.3	184.0	191.6	201.5	145.6	145.7	192.3	164.3	0.268	0.854
Estrogen receptor	ESR1	123.8	131.0	139.1	98.7	117.5	120.4	131.3	112.2	0.089	0.855
Interleukin-3 receptor subunit alpha	IL3RA	131.7	171.6	255.9	115.6	155.0	207.3	186.4	159.3	0.584	0.855
Midkine	MDK	314.1	324.9	320.0	298.0	266.0	256.8	319.7	273.6	0.059	0.856
Microtubule-associated protein tau	MAPT	319.0	320.2	309.8	270.8	274.0	267.8	316.3	270.9	0.001	0.856
Low affinity immunoglobulin gamma Fc region receptor II-a/b	FCGR2A FCGR2B	143.1	106.0	93.3	135.2	75.6	82.4	114.1	97.7	0.534	0.856
Anterior gradient protein 2 homolog	AGR2	347.5	455.3	402.8	333.2	352.2	347.3	401.9	344.2	0.202	0.857
Protein kinase C gamma type	PRKCG	64.4	80.1	51.9	63.0	51.2	54.1	65.5	56.1	0.377	0.857
Tumor necrosis factor receptor superfamily member 11A	TNFRSF11A	372.9	451.9	548.1	312.5	448.8	416.1	457.6	392.5	0.377	0.858
Carbonic anhydrase 1	CA1	156.8	192.2	174.3	148.1	156.5	144.7	174.4	149.8	0.126	0.859
Fibroblast growth factor 18	FGF18	142.4	139.7	129.9	113.5	114.0	126.4	137.3	118.0	0.027	0.859
Insulin-like growth factor-binding protein 7	IGFBP7	235.9	251.4	253.2	225.4	219.1	191.8	246.8	212.1	0.058	0.859
RGM domain family member B	RGMB	267.2	290.2	274.8	227.0	238.0	250.5	277.4	238.5	0.015	0.860
Follistatin	FST	123.5	154.5	143.3	113.6	130.8	118.5	140.4	121.0	0.154	0.861
Inducible T-cell costimulator	ICOS	512.9	458.4	548.5	389.2	399.1	521.8	506.6	436.7	0.249	0.862
Cytochrome P450 3A4	CYP3A4	15774.5	23119.6	15342.1	16158.2	19689.6	10913.3	18078.7	15587.0	0.526	0.862
Cytotoxic and regulatory T-cell molecule	CRTAM	97.4	118.2	112.4	86.8	103.6	92.6	109.3	94.3	0.135	0.863
Beta-Ala-His dipeptidase	CNDP1	367.1	493.2	420.6	356.7	399.6	349.4	427.0	368.6	0.247	0.863
Complement C1s subcomponent	C1S	885.0	1092.2	986.3	774.7	937.3	851.3	987.8	854.4	0.158	0.865
GDNF family receptor alpha-3	GFRA3	508.4	522.7	559.9	429.5	483.0	463.7	530.3	458.7	0.031	0.865
Desert hedgehog protein N-product	DHH	209.1	290.8	263.4	190.0	251.7	218.6	254.4	220.1	0.320	0.865
Matrilin-2	MATN2	42975.5	30609.5	39199.4	34514.3	24903.0	38251.1	37594.8	32556.1	0.404	0.866
Insulin-like growth factor-binding protein 3	IGFBP3	254.0	237.4	274.9	263.5	192.1	208.1	255.4	221.2	0.254	0.866
Fibroblast growth factor receptor 4	FGFR4	450.0	503.1	549.2	369.9	476.9	455.1	500.8	434.0	0.200	0.867
Mammaglobin-B	SCGB2A1	24255.4	21409.1	22231.7	17843.9	19890.1	21118.0	22632.1	19617.3	0.078	0.867

Tumor necrosis factor receptor superfamily member 10A	TNFRSF10A	243.7	307.3	262.7	227.3	245.5	232.8	271.2	235.2	0.189	0.867
Cathepsin S	CTSS	111.3	81.7	76.0	107.9	62.3	63.2	89.7	77.8	0.561	0.868
Dynactin subunit 2	DCTN2	83.0	103.5	115.0	89.5	96.5	75.6	100.5	87.2	0.310	0.868
Adapter molecule crk	CRK	5835.0	6809.0	5909.7	5851.5	5722.7	4528.6	6184.6	5367.6	0.200	0.868
Insulin-degrading enzyme	IDE	1990.2	1836.5	2375.9	2102.1	1623.7	1658.5	2067.5	1794.8	0.287	0.868
Brevican core protein	BCAN	73.5	100.7	103.6	66.0	83.1	92.1	92.6	80.4	0.379	0.868
Fibroblast growth factor 6	FGF6	166.0	218.2	204.6	148.6	197.1	165.8	196.3	170.5	0.290	0.869
Cell adhesion molecule 3	CADM3	266.5	346.3	323.9	243.4	293.7	276.9	312.2	271.3	0.231	0.869
Dual specificity mitogen-activated protein kinase kinase 1	MAP2K1	980.9	956.9	777.8	790.3	845.1	725.6	905.2	787.0	0.201	0.869
Breast cancer anti-estrogen resistance protein 3	BCAR3	883.6	1056.3	1206.7	743.6	1023.4	969.6	1048.9	912.2	0.342	0.870
Thyroxine-Binding Globulin	SERPINA7	175.8	156.7	153.8	164.7	135.1	123.6	162.1	141.1	0.228	0.871
Apolipoprotein D	APOD	2038.5	2661.0	2627.7	1827.3	2504.3	2051.5	2442.4	2127.7	0.330	0.871
Bone morphogenetic protein 6	BMP6	1136.3	1194.5	1315.4	872.2	1122.0	1183.3	1215.4	1059.2	0.243	0.871
Protein kinase C iota type	PRKCI	409.6	455.3	432.5	352.7	410.2	368.5	432.5	377.1	0.067	0.872
Calcium/calmodulin-dependent protein kinase type 1	CAMK1	3091.0	3547.5	3598.1	3328.1	3688.9	1917.0	3412.2	2978.0	0.511	0.873
OX-2 membrane glycoprotein	CD200	218.0	250.9	253.5	191.1	214.9	225.3	240.8	210.4	0.119	0.874
Noggin	NOG	371.4	462.1	486.0	369.3	423.4	361.4	439.8	384.7	0.258	0.875
Persulfide dioxygenase ETHE1, mitochondrial	ETHE1	312.2	305.9	327.0	260.1	258.6	308.8	315.0	275.8	0.128	0.876
Ubiquitin carboxyl-terminal hydrolase isozyme L1	UCHL1	1645.5	1933.6	1958.2	1488.9	2014.6	1345.5	1845.8	1616.3	0.388	0.876
Ubiquitin-fold modifier-conjugating enzyme 1	UFC1	3156.1	3564.1	3559.1	3234.7	3196.5	2576.0	3426.4	3002.4	0.182	0.876
Keratin, type I cytoskeletal 18	KRT18	155.5	188.6	173.0	138.2	162.3	152.7	172.4	151.1	0.153	0.876
Tissue-type plasminogen activator	PLAT	144.8	148.9	141.6	136.5	118.4	126.7	145.1	127.2	0.060	0.877
Dynein light chain roadblock-type 1	DYNLRB1	1069.9	1352.0	963.8	1022.1	922.3	1024.1	1128.6	989.5	0.353	0.877
Protein kinase C theta type	PRKCQ	471.9	527.3	501.3	404.1	456.3	456.4	500.2	438.9	0.061	0.878
Endoplasmic reticulum resident protein 29	ERP29	1194.1	1152.5	1175.3	997.2	986.7	1107.0	1174.0	1030.3	0.054	0.878
Cytoskeleton-associated protein 2	CKAP2	22249.9	21198.9	26509.9	19398.8	21150.1	20858.5	23319.6	20469.1	0.215	0.878
Interleukin-1 Receptor accessory protein	IL1RAP	121422.4	87197.5	108643.5	97847.4	82157.6	98523.0	105754.5	92842.7	0.336	0.878
Focal adhesion kinase 1	PTK2	45.8	47.9	45.9	41.3	40.6	40.7	46.5	40.9	0.009	0.878
Ephrin type-B receptor 4	EPHB4	18304.4	17957.6	19083.9	16509.2	16556.7	15540.2	18448.6	16202.0	0.009	0.878
Calreticulin	CALR	982.4	1136.7	1184.9	830.3	1088.9	983.1	1101.3	967.4	0.241	0.878

Vascular endothelial growth factor C	VEGFC	472.3	446.9	489.9	446.5	370.0	423.8	469.7	413.4	0.115	0.880
Galectin-4	LGALS4	137.3	149.5	141.4	125.0	128.7	123.6	142.7	125.8	0.028	0.881
Interleukin-22 receptor subunit alpha-1	IL22RA1	139.6	171.8	143.3	144.9	131.9	123.9	151.6	133.6	0.219	0.881
Thrombospondin-1	THBS1	150.3	192.2	179.8	145.7	170.9	143.7	174.1	153.4	0.253	0.881
Anti-Muellerian hormone type-2 receptor	AMHR2	10373.4	5753.9	49761.0	8509.1	5242.7	44337.4	21962.8	19363.1	0.897	0.882
3-phosphoinositide-dependent protein kinase 1	PDPK1	145.8	215.1	191.0	170.0	167.2	149.4	184.0	162.2	0.399	0.882
Complement C5b-C6 complex	C5 C6	42.7	44.0	45.4	48.4	34.0	34.1	44.0	38.8	0.391	0.882
Aurora kinase B	AURKB	362.6	361.5	399.8	335.5	334.5	321.3	374.6	330.4	0.059	0.882
Estradiol 17-beta-dehydrogenase 1	HSD17B1	496.8	591.9	620.7	464.9	548.5	495.9	569.8	503.1	0.221	0.883
Coagulation Factor XI	F11	113.8	119.2	116.2	116.4	96.6	95.6	116.4	102.9	0.179	0.884
AMP Kinase (alpha2beta2gamma1)	PRKAA2 PRKAB2 PRKAG1	535.6	791.4	795.2	550.0	784.8	541.3	707.4	625.4	0.523	0.884
Bone sialoprotein 2	IBSP	237.9	165.1	158.1	205.0	149.2	142.7	187.0	165.6	0.546	0.886
Ribosome maturation protein SBDS	SBDS	664.8	1013.8	930.6	658.5	939.3	713.1	869.7	770.3	0.506	0.886
Programmed cell death 1 ligand 1	CD274	190.5	188.6	197.6	175.6	153.0	182.2	192.2	170.3	0.121	0.886
Ficolin-2	FCN2	317.4	351.7	365.8	295.9	329.3	291.8	345.0	305.7	0.105	0.886
Neurotrophin-3	NTF3	70.7	71.6	74.2	56.4	62.9	72.6	72.2	64.0	0.220	0.886
Retinoblastoma-associated protein	RB1	482.0	531.8	522.4	450.4	454.2	457.7	512.1	454.1	0.060	0.887
cGMP-inhibited 3',5'-cyclic phosphodiesterase A	PDE3A	867.8	1016.5	1262.5	676.3	1100.3	1015.8	1048.9	930.8	0.533	0.887
Methionine aminopeptidase 1	METAP1	408.1	923.3	549.6	609.1	685.2	375.9	627.0	556.7	0.720	0.888
Disintegrin and metalloproteinase domain-containing protein 9	ADAM9	264.5	84.0	83.2	216.7	80.7	86.1	143.9	127.8	0.842	0.888
Caspase-3	CASP3	378.4	398.6	418.8	298.4	363.3	400.6	398.6	354.1	0.272	0.888
GRB2-related adapter protein 2	GRAP2	493.3	549.1	608.7	397.8	526.7	542.6	550.4	489.0	0.345	0.889
Heterogeneous nuclear ribonucleoprotein Q	SYNCRIP	527.6	598.2	605.3	531.7	532.7	474.1	577.0	512.8	0.115	0.889
Serine/threonine-protein kinase MRCK beta	CDC42BPB	243.9	318.7	291.4	248.0	269.9	241.8	284.7	253.2	0.285	0.890
Stromal cell-derived factor 1	CXCL12	2834.0	2889.2	2737.2	3264.4	1901.4	2360.4	2820.1	2508.7	0.519	0.890
Afamin	AFM	345.7	331.0	333.9	359.1	272.2	267.9	336.9	299.7	0.337	0.890
Proto-oncogene tyrosine-protein kinase Src	SRC	352.1	552.9	407.2	465.3	418.3	284.1	437.4	389.2	0.584	0.890
Apolipoprotein E (isoform E3)	APOE	157.2	180.4	177.4	150.0	164.7	143.7	171.7	152.8	0.122	0.890
Repulsive guidance molecule A	RGMA	7164.9	5953.6	6162.4	6143.7	5131.1	5887.6	6427.0	5720.8	0.219	0.890

Histone H1.2	HIST1H1C	119.6	241.2	187.2	172.0	173.3	142.5	182.7	162.6	0.632	0.890
T-lymphocyte activation antigen CD80	CD80	242.0	219.5	219.9	240.1	174.5	192.3	227.1	202.3	0.334	0.891
Pituitary adenylate cyclase-activating polypeptide 27	ADCYAP1	608.9	760.5	717.0	555.8	676.5	626.4	695.5	619.6	0.258	0.891
Angiopoietin-1 receptor, soluble	TEK	159.2	182.1	178.1	144.2	162.7	156.0	173.1	154.3	0.106	0.891
Calcium/calmodulin-dependent protein kinase type II subunit alpha	CAMK2A	219.8	272.7	209.5	210.2	207.0	208.7	234.0	208.6	0.324	0.892
Plexin-C1	PLXNC1	73.5	71.9	74.5	75.0	61.0	60.1	73.3	65.4	0.240	0.892
Interleukin-34	IL34	208.3	185.7	192.1	211.1	159.9	151.9	195.4	174.3	0.377	0.892
S-phase kinase-associated protein 1	SKP1	1378.8	1480.7	1489.6	1210.2	1348.7	1321.3	1449.7	1293.4	0.049	0.892
Carbonic anhydrase 4	CA4	607.8	568.1	612.5	616.2	492.2	487.6	596.1	532.0	0.264	0.892
Neutrophil-activating peptide 2	PPBP	190.8	189.1	186.7	184.0	162.9	158.8	188.9	168.6	0.118	0.893
Protein kinase C zeta type	PRKCZ	455.9	522.7	436.0	390.4	456.9	415.9	471.5	421.1	0.203	0.893
Cadherin-15	CDH15	1305.8	1651.2	1809.0	1249.1	1573.6	1436.7	1588.7	1419.8	0.400	0.894
Collagen alpha-1(VIII) chain	COL8A1	101.5	73.5	574.3	102.8	70.3	496.7	249.8	223.3	0.907	0.894
Netrin-4	NTN4	287.3	306.1	288.0	244.2	247.5	296.3	293.8	262.7	0.198	0.894
Cytokine receptor-like factor 2	CRLF2	274.0	308.2	308.0	242.0	289.3	265.3	296.7	265.5	0.156	0.895
AMP Kinase (alpha1beta1gamma1)	PRKAA1 PRKAB1 PRKAG1	244.5	125.7	124.3	217.8	110.0	114.8	164.8	147.5	0.761	0.895
Ubiquitin+1, truncated mutation for UbB	RPS27A	35657.6	41209.5	46889.9	36815.1	40104.4	33944.4	41252.3	36954.6	0.327	0.896
Interleukin-3	IL3	551.0	709.9	596.0	534.3	557.9	575.7	619.0	556.0	0.313	0.898
Bactericidal permeability-increasing protein	BPI	438.6	495.3	470.5	370.3	446.3	445.5	468.1	420.7	0.201	0.899
CD48 antigen	CD48	99.0	121.6	105.1	97.0	95.8	100.3	108.6	97.7	0.246	0.900
MAP kinase-activated protein kinase 3	MAPKAPK3	270.7	398.4	260.3	280.1	285.3	271.3	309.8	278.9	0.559	0.900
Kynureninase	KYNU	127.5	137.4	136.4	130.4	120.7	110.2	133.8	120.4	0.136	0.900
GTPase KRas	KRAS	115.5	130.8	129.2	103.2	117.1	118.0	125.2	112.8	0.143	0.901
High affinity nerve growth factor receptor	NTRK1	849.9	882.6	904.4	675.8	820.6	879.7	879.0	792.0	0.285	0.901
Mitogen-activated protein kinase 3	MAPK3	6305.4	6474.1	6385.3	5577.8	5800.3	5896.6	6388.3	5758.2	0.010	0.901
Non-receptor tyrosine-protein kinase TYK2	TYK2	6378.3	6864.7	7865.9	5256.6	6977.2	6795.7	7036.3	6343.2	0.380	0.901
Protein jagged-2	JAG2	116.2	126.1	131.0	111.2	112.3	113.4	124.4	112.3	0.105	0.902
Apolipoprotein B	APOB	115.9	99.0	111.0	105.6	88.7	99.9	108.6	98.1	0.209	0.903

Cathepsin H	CTSH	264.6	318.6	315.7	240.3	290.2	281.3	299.6	270.6	0.282	0.903
Matrix metalloproteinase-16	MMP16	439.7	513.0	554.1	392.5	488.9	479.6	502.3	453.7	0.345	0.903
Alpha-soluble NSF attachment protein	NAPA	194.0	230.3	218.0	183.0	219.1	178.3	214.1	193.5	0.287	0.904
Tyrosine-protein kinase Tec	TEC	138.3	213.3	166.0	159.2	163.1	145.9	172.5	156.1	0.534	0.905
Membrane metallo-endopeptidase-like 1	MMEL1	337.7	398.8	391.8	314.3	348.8	357.8	376.1	340.3	0.210	0.905
Platelet endothelial cell adhesion molecule	PECAM1	178.3	178.9	181.2	163.3	160.2	164.1	179.5	162.5	0.001	0.906
Leukotriene A-4 hydrolase	LTA4H	363.8	410.1	512.3	330.5	452.5	382.3	428.7	388.4	0.516	0.906
Cytosolic non-specific dipeptidase	CNDP2	445.1	459.5	419.8	509.8	358.9	331.5	441.5	400.1	0.535	0.906
Translationally-controlled tumor protein	TPT1	80441.7	99502.7	114890.1	80980.3	101610.9	85292.9	98278.2	89294.7	0.495	0.909
Hemojuvelin	HFE2	30621.8	22818.2	26097.2	26287.7	21875.6	24112.9	26512.4	24092.1	0.417	0.909
Eukaryotic initiation factor 4A-III	EIF4A3	125.6	189.8	154.8	142.5	150.2	134.6	156.7	142.4	0.525	0.909
Interleukin-27 receptor subunit alpha	IL27RA	149.5	154.6	147.6	149.0	129.7	131.8	150.6	136.8	0.143	0.909
Interleukin-17 receptor C	IL17RC	313.5	272.6	298.8	273.3	265.7	265.2	295.0	268.1	0.148	0.909
alpha-2-macroglobulin receptor-associated protein	LRPAP1	107.7	173.6	123.3	130.7	132.8	104.5	134.9	122.7	0.618	0.910
Mediator of RNA polymerase II transcription subunit 1	MED1	105.8	132.3	132.8	106.4	118.4	112.6	123.6	112.5	0.339	0.910
Kininogen-1	KNG1	83.2	115.6	104.6	83.4	106.6	86.0	101.1	92.0	0.492	0.910
Bone morphogenetic protein 10	BMP10	2197.5	2084.1	2190.8	1654.3	1896.5	2346.2	2157.5	1965.7	0.445	0.911
Mesothelin	MSLN	108.8	140.5	125.2	99.0	128.8	113.7	124.8	113.8	0.431	0.912
Abelson tyrosine-protein kinase 2	ABL2	906.3	1040.5	1213.2	747.3	1079.1	1058.3	1053.3	961.6	0.547	0.913
Urokinase plasminogen activator surface receptor	PLAUR	320.6	349.1	329.1	285.0	318.1	308.8	332.9	304.0	0.091	0.913
Ubiquitin-fold modifier 1	UFM1	750.8	753.3	648.4	681.4	726.9	558.2	717.5	655.5	0.374	0.914
Interleukin-12 receptor subunit beta-2	IL12RB2	212.5	204.1	215.4	192.9	184.6	200.0	210.7	192.5	0.035	0.914
High affinity cAMP-specific 3',5'-cyclic phosphodiesterase 7A	PDE7A	122.0	130.6	125.9	109.3	123.7	113.0	126.2	115.3	0.112	0.914
Peptidyl-prolyl cis-trans isomerase A	PPIA	126520.4	107245.1	128873.2	104496.5	111528.1	115514.5	120879.6	110513.0	0.269	0.914
Activated Protein C	PROC	251.1	275.5	278.4	220.5	252.6	263.1	268.3	245.4	0.222	0.915
Peptidyl-prolyl cis-trans isomerase F, mitochondrial	PPIF	1294.8	1523.2	1583.4	1157.0	1430.1	1443.5	1467.1	1343.5	0.390	0.916
Tyrosine-protein kinase transmembrane receptor ROR1	ROR1	154.4	174.3	159.3	141.9	144.2	160.9	162.7	149.0	0.182	0.916
Protein FAM107A	FAM107A	110.0	110.1	113.4	99.0	102.8	103.9	111.2	101.9	0.009	0.917

Tenascin	TNC	182.1	178.8	171.5	182.1	151.4	154.6	177.5	162.7	0.265	0.917
Growth/differentiation factor 11	GDF11	6562.6	5242.9	5663.6	5594.7	5460.5	4962.3	5823.0	5339.2	0.348	0.917
Contactin-4	CNTN4	648.4	697.6	714.9	614.9	639.0	637.0	687.0	630.3	0.090	0.918
Nucleoside diphosphate kinase A	NME1	1437.9	1809.5	1624.9	1332.3	1651.9	1487.0	1624.1	1490.4	0.399	0.918
Tumor necrosis factor receptor superfamily member 14	TNFRSF14	54.2	59.9	59.3	55.0	51.9	52.4	57.8	53.1	0.104	0.919
Calpain I	CAPN1 CAPNS1	16995.1	19549.2	22640.4	19077.2	17461.2	17840.6	19728.2	18126.3	0.433	0.919
Matrilysin	MMP7	158.2	175.5	165.9	154.7	154.0	150.4	166.5	153.0	0.106	0.919
Cathepsin G	CTSG	390.0	393.4	406.1	322.9	375.8	395.6	396.5	364.8	0.279	0.920
Complement component C7	C7	100.7	113.6	107.5	103.2	99.2	93.9	107.3	98.8	0.145	0.921
C-C motif chemokine 17	CCL17	140.5	133.2	136.5	139.1	115.3	123.3	136.7	125.9	0.258	0.921
Cadherin-5	CDH5	29001.6	22781.8	25729.9	25052.8	21768.5	24574.6	25837.8	23798.6	0.393	0.921
Toll-like receptor 4:Lymphocyte antigen 96 complex	TLR4 LY96	137.2	204.7	147.1	156.7	158.8	135.1	163.0	150.2	0.614	0.921
Peptidyl-prolyl cis-trans isomerase B	PPIB	1507.7	1563.7	1624.4	1202.6	1464.9	1661.6	1565.3	1443.0	0.458	0.922
Interleukin-17 receptor D	IL17RD	82.8	83.6	80.9	76.4	76.4	75.2	82.4	76.0	0.006	0.922
40S ribosomal protein S3	RPS3	92.8	123.8	110.2	104.7	111.2	85.4	108.9	100.4	0.514	0.922
Neurogenic locus notch homolog protein 2	NOTCH2	182.4	169.3	175.3	165.5	150.5	169.9	175.7	162.0	0.133	0.922
Lamin-B1	LMNB1	126.9	158.0	155.2	122.4	147.0	136.4	146.7	135.3	0.408	0.922
C-C motif chemokine 27	CCL27	141.4	161.2	149.7	134.5	147.6	135.0	150.8	139.0	0.183	0.922
Delta-like protein 4	DLL4	74.7	81.9	76.3	70.5	67.6	76.9	77.6	71.7	0.168	0.923
Methyl-CpG-binding domain protein 4	MBD4	117.5	112.7	498.1	104.3	98.2	469.9	242.8	224.1	0.921	0.923
CD226 antigen	CD226	494.6	488.8	545.3	404.0	483.3	524.8	509.6	470.7	0.401	0.924
Growth arrest-specific protein 1	GAS1	241.3	254.0	270.3	226.6	241.0	240.2	255.2	235.9	0.135	0.925
Glia-derived nexin	SERPINE2	405.9	436.5	396.4	382.1	412.8	350.9	412.9	381.9	0.234	0.925
Histone acetyltransferase KAT6A	KAT6A	206.2	229.4	212.4	226.5	198.9	175.0	216.0	200.1	0.409	0.927
Macrophage colony-stimulating factor 1	CSF1	225.5	267.4	244.1	212.3	242.4	228.3	245.7	227.7	0.300	0.927
Leptin	LEP	245.4	293.1	301.8	241.1	269.8	267.9	280.1	259.6	0.376	0.927
L-lactate dehydrogenase B chain	LDHB	28401.4	32882.8	25126.9	24329.0	27697.4	28082.2	28803.7	26702.9	0.469	0.927
Complement component 1 Q subcomponent-binding protein, mitochondrial	C1QBP	14389.1	12627.4	13869.5	11930.5	12476.8	13557.4	13628.7	12654.9	0.242	0.929
Eotaxin	CCL11	122.3	105.7	110.5	110.3	101.4	103.3	112.8	105.0	0.255	0.931
Cathepsin E	CTSE	76.8	81.3	72.4	73.4	71.8	69.4	76.8	71.5	0.164	0.931
Semaphorin-3A	SEMA3A	135.1	149.5	176.2	128.4	135.8	165.0	153.6	143.1	0.556	0.931

Sialic acid-binding Ig-like lectin 14	SIGLEC14	281.2	274.4	251.6	225.6	227.8	298.5	269.1	250.6	0.531	0.931
Granulocyte colony-stimulating factor receptor	CSF3R	533.9	488.9	514.5	437.4	481.7	513.1	512.4	477.4	0.257	0.932
Group 10 secretory phospholipase A2	PLA2G10	140.4	134.3	132.6	139.9	122.3	117.3	135.8	126.5	0.308	0.932
CD40 ligand	CD40LG	115.4	123.0	122.2	108.4	108.2	119.5	120.2	112.0	0.152	0.932
Parathyroid hormone	PTH	112.8	120.5	118.7	101.0	108.0	119.1	117.3	109.4	0.268	0.932
C-C motif chemokine 3	CCL3	221.2	183.9	184.0	181.7	180.0	187.5	196.4	183.1	0.397	0.932
Interleukin-4	IL4	98.7	101.2	100.6	94.0	87.3	98.9	100.2	93.4	0.177	0.932
SLAM family member 7	SLAMF7	172.1	186.2	208.0	161.8	171.5	194.9	188.8	176.1	0.426	0.933
Phosphatidylinositol 4,5-bisphosphate 3-kinase catalytic subunit alpha isoform:Phosphatidylinositol 3-kinase regulatory subunit alpha complex	PIK3CA PIK3R1	855.7	754.3	724.1	835.5	684.5	657.4	778.0	725.8	0.491	0.933
Transcription factor IIIB 90 kDa subunit	BRF1	444.9	517.1	602.3	429.6	554.0	476.0	521.4	486.5	0.583	0.933
GDNF family receptor alpha-1	GFRA1	259.3	262.3	248.5	254.1	214.3	250.8	256.7	239.7	0.314	0.934
Interleukin-18 receptor accessory protein	IL18RAP	133.1	78.8	82.1	125.7	71.9	77.1	98.0	91.6	0.806	0.934
C-C motif chemokine 2	CCL2	57.2	68.4	66.4	57.3	60.5	61.6	64.0	59.8	0.350	0.934
Tumor necrosis factor receptor superfamily member 8	TNFRSF8	259.2	262.1	285.2	254.9	266.7	232.4	268.8	251.3	0.252	0.935
Carbonic anhydrase 13	CA13	104.9	117.8	114.2	101.8	115.4	97.9	112.3	105.0	0.335	0.935
N-acetyl-D-glucosamine kinase	NAGK	65.5	95.4	79.2	67.9	87.1	69.6	80.0	74.9	0.654	0.935
Fibroblast growth factor 9	FGF9	145.7	144.9	148.0	124.9	133.3	152.1	146.2	136.8	0.361	0.935
Protein SET	SET	2764.6	2745.0	2392.8	2108.1	2262.0	3023.8	2634.1	2464.6	0.624	0.936
Alpha-1-antichymotrypsin	SERPINA3	63.6	91.6	81.3	63.4	93.6	64.3	78.8	73.8	0.714	0.936
Interleukin-18 receptor 1	IL18R1	260.1	227.4	240.6	262.9	205.5	213.5	242.7	227.3	0.503	0.937
SLAM family member 5	CD84	2494.1	2403.0	2804.6	1935.9	2448.5	2829.9	2567.2	2404.8	0.612	0.937
cAMP-regulated phosphoprotein 19	ARPP19	77.4	84.0	79.3	76.1	74.1	75.5	80.2	75.2	0.116	0.938
AT-rich interactive domain-containing protein 3A	ARID3A	222.6	267.7	253.6	204.3	256.3	237.1	248.0	232.6	0.489	0.938
Cystatin-SA	CST2	166.7	114.2	129.8	127.0	118.4	140.1	136.9	128.5	0.656	0.939
OCIA domain-containing protein 1	OCIAD1	598.1	693.3	803.4	570.1	734.8	664.0	698.3	656.3	0.612	0.940
Proto-oncogene tyrosine-protein kinase receptor Ret	RET	239.7	227.6	232.8	234.8	211.4	212.0	233.4	219.4	0.204	0.940
Secreted frizzled-related protein 1	SFRP1	343.1	325.3	307.7	271.6	331.3	314.8	325.4	305.9	0.409	0.940
Galectin-8	LGALS8	55.3	51.9	50.9	50.0	48.2	50.6	52.7	49.6	0.131	0.941

Neutral ceramidase	ASAH2	253.8	259.5	302.1	246.4	275.6	245.8	271.8	255.9	0.439	0.942
Fibroblast growth factor 20	FGF20	129.8	119.2	122.1	118.9	114.0	116.6	123.7	116.5	0.137	0.942
D-dimer	FGA FGB FGG	74008.4	68586.8	73886.4	60454.1	60684.8	82941.2	72160.5	68026.7	0.639	0.943
CD109 antigen	CD109	189.9	196.0	201.7	177.7	185.5	191.0	195.9	184.7	0.098	0.943
NKG2D ligand 1	ULBP1	102.4	119.6	110.6	104.4	104.1	105.2	110.9	104.6	0.332	0.943
Heat shock protein HSP 90-alpha/beta	HSP90AA1 HSP90AB1	119230.0	103518.9	108126.5	97644.2	110442.3	104157.5	110291.8	104081.3	0.358	0.944
Arylsulfatase A	ARSA	140.1	146.8	146.7	127.9	145.4	135.9	144.5	136.4	0.245	0.944
Tumor necrosis factor receptor superfamily member 1A	TNFRSF1A	161.6	191.4	169.1	147.2	162.0	183.6	174.0	164.3	0.521	0.944
Cystatin-F	CST7	208.0	208.1	201.8	183.2	197.0	203.4	206.0	194.5	0.187	0.944
Haptoglobin	HP	64.2	77.5	73.6	74.7	66.9	61.9	71.8	67.8	0.509	0.945
Platelet-derived growth factor receptor beta	PDGFRB	246.4	235.8	247.1	231.7	229.7	228.0	243.1	229.8	0.058	0.945
Fibronectin Fragment 3	FN1	183.2	192.2	186.2	157.2	178.4	195.8	187.2	177.1	0.465	0.946
Tumor necrosis factor receptor superfamily member 13C	TNFRSF13C	411.0	444.8	538.0	400.8	473.5	445.0	464.6	439.8	0.606	0.947
Dentin matrix acidic phosphoprotein 1	DMP1	292.8	279.1	276.3	248.5	271.7	282.9	282.7	267.7	0.278	0.947
Leucine carboxyl methyltransferase 1	LCMT1	1275.1	1107.0	1257.7	1119.1	1204.2	1124.4	1213.3	1149.2	0.365	0.947
Interleukin-12	IL12A IL12B	381.9	382.7	358.5	373.8	350.3	339.8	374.4	354.6	0.202	0.947
Tumor necrosis factor receptor superfamily member 6B	TNFRSF6B	70.8	86.0	69.8	78.7	66.6	69.4	75.5	71.6	0.572	0.947
Thymic stromal lymphopoietin	TSLP	99.8	102.0	110.5	99.6	98.3	98.0	104.1	98.6	0.234	0.947
Small glutamine-rich tetratricopeptide repeat-containing protein alpha	SGTA	717.6	840.6	763.7	743.1	748.9	710.3	774.0	734.1	0.385	0.948
B-cell receptor CD22	CD22	538.9	528.5	562.6	514.6	518.4	513.2	543.3	515.4	0.106	0.949
Nuclear receptor subfamily 1 group D member 1	NR1D1	83.8	90.4	92.3	84.1	83.1	85.6	88.8	84.3	0.213	0.949
MAP kinase-activated protein kinase 5	MAPKAPK5	250.6	223.5	215.0	224.3	207.8	221.8	229.7	218.0	0.400	0.949
Junctional adhesion molecule-like	AMICA1	128.0	126.3	125.0	129.3	110.8	120.0	126.4	120.0	0.353	0.949
Ectonucleoside triphosphate diphosphohydrolase 5	ENTPD5	263.7	291.2	294.4	252.7	288.5	265.3	283.1	268.8	0.376	0.950
Angiogenin	ANG	128.6	139.6	123.0	101.3	87.2	183.0	130.4	123.8	0.847	0.950
Epidermal growth factor receptor substrate 15-like 1	EPS15L1	105.4	109.1	103.9	100.3	104.1	98.2	106.1	100.9	0.086	0.950
Hepatocyte growth factor	HGF	186.3	210.3	206.5	174.0	192.3	207.2	201.0	191.2	0.465	0.951
Interferon alpha-2	IFNA2	29.6	37.6	28.0	29.9	28.7	32.0	31.7	30.2	0.664	0.952

Tumor necrosis factor ligand superfamily member 8	TNFSF8	225.4	240.7	229.6	226.4	215.8	220.8	231.9	221.0	0.128	0.953
Pescadillo homolog	PES1	156.7	166.3	168.8	141.2	157.3	170.4	163.9	156.3	0.473	0.953
Bone morphogenetic protein receptor type-2	BMPR2	188.3	171.1	167.4	185.8	157.7	158.8	175.6	167.4	0.511	0.953
Serine/threonine-protein kinase TBK1	TBK1	123.6	137.5	139.9	123.9	132.7	126.1	133.7	127.6	0.365	0.954
Phospholipase A2, membrane associated	PLA2G2A	234.7	215.3	231.4	231.1	198.5	221.4	227.1	217.0	0.432	0.955
SH2 domain-containing protein 1A	SH2D1A	719.7	682.3	764.4	513.4	661.5	896.0	722.1	690.3	0.804	0.956
Ficolin-1	FCN1	103.0	114.8	95.1	101.3	99.9	98.0	104.3	99.7	0.510	0.956
Fibroblast growth factor 10	FGF10	64.8	63.1	62.1	61.1	57.9	62.7	63.3	60.6	0.181	0.956
Discoidin domain-containing receptor 2	DDR2	2395.9	2378.4	2874.7	1914.5	2576.4	2825.9	2549.7	2438.9	0.748	0.957
Plasma serine protease inhibitor	SERPINA5	249.4	257.8	258.7	251.1	249.7	232.3	255.3	244.4	0.206	0.957
Parathyroid hormone-related protein	PTHLH	265.5	253.7	298.7	277.0	219.2	286.9	272.6	261.0	0.671	0.957
Interleukin-1 beta	IL1B	3067.4	2767.0	3002.7	2391.2	2668.2	3404.6	2945.7	2821.3	0.727	0.958
Peroxiredoxin-1	PRDX1	5537.5	7163.0	6211.7	5219.5	8831.1	4065.6	6304.1	6038.7	0.874	0.958
Intercellular adhesion molecule 1	ICAM1	1929.2	1655.4	1753.5	1654.3	1673.4	1786.0	1779.4	1704.6	0.467	0.958
Chitotriosidase-1	CHIT1	224.9	304.5	236.3	251.7	257.5	224.4	255.2	244.5	0.720	0.958
Sphingosine kinase 1	SPHK1	395.2	302.9	475.3	326.9	396.0	401.4	391.1	374.8	0.787	0.958
R-spondin-2	RSPO2	126.2	139.2	141.5	120.6	137.2	132.2	135.6	130.0	0.457	0.958
Protein amnionless	AMN	161.7	170.3	162.3	141.6	163.1	169.1	164.8	157.9	0.506	0.959
Alanine aminotransferase 1	GPT	11572.4	10939.9	11752.9	10616.1	11557.0	10680.4	11421.7	10951.2	0.298	0.959
Tissue factor pathway inhibitor	TFPI	293.9	324.0	305.9	252.6	293.8	339.4	307.9	295.3	0.672	0.959
C-X-C motif chemokine 16	CXCL16	491.8	532.6	518.6	501.0	490.3	488.7	514.3	493.3	0.215	0.959
Transforming growth factor beta-3	TGFB3	124.8	119.1	113.9	112.6	114.2	116.4	119.3	114.4	0.258	0.959
Disintegrin and metalloproteinase domain-containing protein 12	ADAM12	177.9	170.3	196.0	152.3	167.3	202.4	181.4	174.0	0.688	0.959
Neutrophil elastase	ELANE	121.7	121.8	131.6	113.6	115.7	130.5	125.0	119.9	0.469	0.959
Coagulation Factor VII	F7	166.3	175.2	177.7	173.2	167.3	157.6	173.1	166.0	0.290	0.959
Matrix metalloproteinase-9	MMP9	240.5	273.2	247.5	221.0	241.3	268.0	253.7	243.4	0.577	0.959
Cell surface glycoprotein CD200 receptor 1	CD200R1	99.1	96.8	106.4	98.0	90.3	101.9	100.8	96.7	0.419	0.960
Tumor necrosis factor receptor superfamily member 1B	TNFRSF1B	545.5	624.6	577.9	566.7	553.5	558.2	582.7	559.5	0.419	0.960
Tryptase beta-2	TPSB2	268.9	232.0	270.9	244.3	251.6	245.4	257.3	247.1	0.507	0.960
Tumor necrosis factor ligand superfamily member 15	TNFSF15	211.1	193.6	211.7	197.2	185.1	210.2	205.5	197.5	0.445	0.961

Gelsolin	GSN	101760.9	76633.3	94124.8	87520.2	85778.6	88667.8	90839.7	87322.2	0.684	0.961
NKG2-D type II integral membrane protein	KLRK1	134.1	359.5	138.6	139.1	328.6	140.4	210.7	202.7	0.938	0.962
Hepcidin	HAMP	115.4	112.6	116.2	110.6	110.2	110.6	114.7	110.5	0.058	0.963
Dickkopf-related protein 3	DKK3	178.2	191.4	169.3	170.8	166.1	182.2	179.6	173.0	0.459	0.963
Fibroblast growth factor 5	FGF5	107.1	114.9	102.5	103.5	103.8	105.6	108.2	104.3	0.398	0.964
N-acetylglucosamine-6-sulfatase	GNS	143.0	173.1	134.1	142.9	151.2	140.1	150.1	144.7	0.701	0.964
Kallikrein-4	KLK4	77.3	76.4	82.2	73.3	75.8	78.5	78.6	75.9	0.306	0.965
Cardiotrophin-1	CTF1	208.0	173.3	183.2	200.1	168.1	176.7	188.2	181.6	0.667	0.965
Kin of IRRE-like protein 3	KIRREL3	75.5	66.1	67.1	72.3	65.5	63.7	69.6	67.2	0.578	0.966
Interleukin-15 receptor subunit alpha	IL15RA	212.2	223.4	203.1	198.2	210.5	208.0	212.9	205.6	0.362	0.966
cAMP-specific 3',5'-cyclic phosphodiesterase 4D	PDE4D	405.4	498.1	460.7	400.6	489.5	428.6	454.7	439.6	0.707	0.967
Kallikrein-8	KLK8	187.0	181.5	192.0	180.8	185.3	175.8	186.8	180.6	0.205	0.967
Coagulation factor IXab	F9	35.8	39.4	34.9	33.4	36.5	36.6	36.7	35.5	0.529	0.967
Granulysin	GNLY	28.6	34.9	30.3	32.9	27.3	30.8	31.3	30.3	0.727	0.970
Cystatin-S	CST4	27.6	30.4	29.9	29.6	26.4	29.3	29.3	28.4	0.553	0.970
C-X-C motif chemokine 10	CXCL10	119.9	117.5	111.5	105.5	116.7	116.4	116.3	112.9	0.489	0.970
Interleukin-7 receptor subunit alpha	IL7R	90.8	94.4	92.7	91.2	88.4	90.2	92.6	89.9	0.115	0.971
Cyclin-dependent kinase 1:G2/mitotic-specific cyclin-B1 complex	CDC2 CCNB1	216.5	183.7	183.3	210.5	182.3	174.6	194.5	189.1	0.747	0.972
Phospholipase A2	PLA2G1B	237.5	196.8	195.6	206.9	224.2	181.8	210.0	204.3	0.774	0.973
Glucocorticoid receptor	NR3C1	43.9	55.1	49.1	45.3	51.9	47.2	49.4	48.1	0.764	0.975
Tropomyosin alpha-1 chain	TPM1	166.0	188.8	169.8	172.7	176.6	162.3	174.9	170.5	0.632	0.975
Neurogenic locus notch homolog protein 1	NOTCH1	5684.8	4849.0	5062.1	5017.4	4782.9	5411.7	5198.6	5070.7	0.703	0.975
Kallistatin	SERPINA4	130.6	117.0	126.5	134.2	111.5	119.5	124.7	121.7	0.726	0.976
C-type lectin domain family 4 member M	CLEC4M	58989.1	48111.2	52736.5	46132.0	50569.4	59383.7	53278.9	52028.4	0.816	0.977
Ectonucleoside triphosphate diphosphohydrolase 1	ENTPD1	808.9	725.9	761.2	785.3	725.1	731.8	765.3	747.4	0.592	0.977
Fibroblast growth factor 2	FGF2	202.6	217.4	202.2	188.9	176.0	242.8	207.4	202.6	0.838	0.977
Dipeptidyl peptidase 2	DPP7	2772.1	2629.3	2514.9	2401.6	2488.6	2844.0	2638.8	2578.1	0.720	0.977
Coagulation factor Xa	F10	118651.8	96666.1	112394.9	104199.1	101920.7	114209.4	109237.6	106776.4	0.765	0.977
Interferon lambda-1	IFNL1	94.8	105.0	106.5	86.2	106.4	106.9	102.1	99.8	0.788	0.978
Protein lin-7 homolog B	LIN7B	174.7	171.9	179.8	189.0	167.2	159.1	175.5	171.8	0.723	0.979
Corticosteroid-binding globulin	SERPINA6	93.4	113.1	108.4	99.7	103.4	105.4	105.0	102.8	0.758	0.980

C-C motif chemokine 13	CCL13	124.7	118.6	122.6	121.5	113.5	123.5	122.0	119.5	0.533	0.980
Artemin	ARTN	166.5	163.3	157.9	154.4	158.2	165.5	162.6	159.4	0.483	0.980
Serum amyloid A-1 protein	SAA1	129.9	120.3	132.1	118.1	132.3	124.5	127.4	125.0	0.676	0.981
Tumor necrosis factor receptor superfamily member 18	TNFRSF18	187.0	188.9	197.9	190.3	188.4	184.3	191.3	187.7	0.413	0.981
TGF-beta receptor type-2	TGFBR2	138.4	144.2	152.4	140.9	144.8	141.6	145.0	142.4	0.598	0.982
dCTP pyrophosphatase 1	DCTPP1	1536.8	1430.9	1338.3	1812.9	1361.5	1055.6	1435.3	1410.0	0.920	0.982
Histidine triad nucleotide-binding protein 1	HINT1	96.1	93.8	93.5	90.9	92.5	95.0	94.5	92.8	0.321	0.982
Phosphatidylinositol 3,4,5-trisphosphate 3-phosphatase and dual-specificity protein phosphatase PTEN	PTEN	218.6	201.6	225.0	198.3	201.1	234.6	215.1	211.3	0.800	0.983
Leptin receptor	LEPR	73.5	83.1	80.0	82.8	77.6	72.1	78.9	77.5	0.761	0.983
Lymphocyte activation gene 3 protein	LAG3	27.2	33.0	26.7	30.2	30.9	24.3	29.0	28.5	0.872	0.983
Tumor necrosis factor receptor superfamily member 17	TNFRSF17	88.3	90.4	88.8	78.8	88.4	95.7	89.2	87.6	0.785	0.983
Natural cytotoxicity triggering receptor 3	NCR3	71.8	69.3	69.2	63.2	71.4	72.1	70.1	68.9	0.721	0.983
Transmembrane glycoprotein NMB	GPNMB	20.3	25.2	20.5	21.4	19.8	23.7	22.0	21.6	0.862	0.983
Tyrosine-protein kinase Lck	LCK	154.1	128.8	127.8	147.2	127.0	129.8	136.9	134.7	0.845	0.984
Urokinase-type plasminogen activator	PLAU	183.4	201.9	194.3	186.8	201.5	182.1	193.2	190.1	0.719	0.984
WNT1-inducible-signaling pathway protein 1	WISP1	12711.2	11512.8	11422.2	10439.7	12001.1	12642.5	11882.1	11694.4	0.823	0.984
Interleukin-11	IL11	153.0	157.9	155.5	134.7	153.0	171.4	155.5	153.0	0.840	0.984
Killer cell immunoglobulin-like receptor 3DS1	KIR3DS1	86.5	91.5	91.1	92.7	84.3	88.1	89.7	88.4	0.674	0.985
Hepatoma-derived growth factor-related protein 2	HDGFRP2	166.4	166.4	172.1	165.4	163.1	168.9	168.3	165.8	0.382	0.985
Carbohydrate sulfotransferase 2	CHST2	85.2	106.6	99.7	91.1	109.6	86.5	97.2	95.7	0.887	0.985
Ephrin type-A receptor 5	EPHA5	73.0	85.6	79.2	80.0	80.3	74.1	79.3	78.1	0.802	0.986
Apolipoprotein A-I	APOA1	79.8	78.8	80.0	84.4	77.0	73.9	79.5	78.4	0.758	0.986
Cadherin-6	CDH6	161750.4	125021.9	147063.9	134636.4	136032.5	157437.1	144612.1	142702.0	0.891	0.987
Roundabout homolog 3	ROBO3	47.3	51.1	54.9	48.0	53.0	50.3	51.1	50.4	0.814	0.987
Syntaxin-1A	STX1A	79.8	78.1	73.7	79.9	76.0	72.7	77.2	76.2	0.736	0.987
Complement factor I	CFI	17857.6	15310.7	17171.8	15356.9	16357.6	17980.4	16780.0	16565.0	0.852	0.987
T-lymphocyte activation antigen CD86	CD86	52.6	52.4	59.9	51.7	49.2	61.9	55.0	54.3	0.888	0.987

Cytoplasmic protein NCK1	NCK1	181.4	164.8	176.7	179.1	171.5	165.8	174.3	172.1	0.748	0.988
Tyrosine-protein kinase receptor TYRO3	TYRO3	182.0	155.2	160.5	184.1	147.8	159.8	165.9	163.9	0.890	0.988
Endoglin	ENG	48.3	28.2	31.9	42.0	31.2	34.0	36.1	35.7	0.958	0.989
Angiostatin	PLG	43.8	55.3	46.6	50.5	50.7	42.9	48.6	48.0	0.908	0.989
Tumor necrosis factor receptor superfamily member 19L	RELT	37.1	39.0	38.5	38.5	38.0	36.9	38.2	37.8	0.618	0.990
Tyrosine-protein kinase ABL1	ABL1	92.5	87.6	97.4	83.5	99.4	91.7	92.5	91.5	0.868	0.990
Epithelial discoidin domain-containing receptor 1	DDR1	91.1	88.7	88.3	87.1	85.7	92.5	89.4	88.4	0.709	0.990
Integrin alpha-IIb: beta-3 complex	ITGA2B ITGB3	99.3	110.5	106.2	98.6	113.0	101.2	105.3	104.3	0.857	0.990
MAP kinase-activated protein kinase 2	MAPKAPK2	263.3	255.1	256.4	246.7	263.3	257.0	258.3	255.7	0.667	0.990
Prolactin receptor	PRLR	718.5	696.6	737.6	674.3	738.0	719.5	717.6	710.6	0.773	0.990
Interleukin-10 receptor subunit beta	IL10RB	238.4	218.0	228.1	236.0	221.2	220.9	228.2	226.0	0.796	0.991
alpha-L-iduronidase	IDUA	132.0	139.9	140.4	133.4	142.0	133.3	137.4	136.2	0.777	0.991
Interleukin-8	CXCL8	218.4	207.7	220.3	215.6	208.4	216.8	215.5	213.6	0.715	0.991
Ck-beta-8-1	CCL23	73.8	77.6	73.7	74.0	72.1	77.1	75.0	74.4	0.761	0.992
Mitogen-activated protein kinase 11	MAPK11	139.3	115.9	149.2	125.4	121.4	154.3	134.8	133.7	0.942	0.992
Wnt inhibitory factor 1	WIF1	6422.9	5326.9	6945.3	6885.3	4691.4	6979.4	6231.7	6185.4	0.961	0.993
Ferritin	FTH1 FTL	113.8	93.9	122.9	110.2	97.1	121.0	110.2	109.4	0.948	0.993
Matrix metalloproteinase-14	MMP14	203.8	183.8	193.9	193.2	190.8	193.5	193.8	192.5	0.840	0.993
Tyrosine-protein kinase Fyn	FYN	456.2	461.3	428.1	505.9	412.2	418.4	448.5	445.5	0.932	0.993
Tumor necrosis factor ligand superfamily member 12	TNFSF12	124.0	130.8	122.0	120.8	119.0	134.6	125.6	124.8	0.895	0.994
beta-nerve growth factor	NGF	83.2	89.1	101.0	77.9	95.2	98.6	91.1	90.6	0.952	0.994
Tumor necrosis factor ligand superfamily member 14	TNFSF14	61.7	67.5	64.5	71.7	60.2	60.7	64.6	64.2	0.935	0.994
Eukaryotic translation initiation factor 5	EIF5	420.2	696.2	1039.1	635.6	929.1	578.9	718.5	714.5	0.986	0.994
Baculoviral IAP repeat-containing protein 5	BIRC5	187.9	183.4	191.9	189.2	181.8	189.4	187.7	186.8	0.803	0.995
Kallikrein-11	KLK11	166.0	165.6	171.4	156.7	168.8	175.0	167.7	166.8	0.895	0.995
Layilin	LAYN	291.6	280.5	280.1	287.9	280.8	279.4	284.1	282.7	0.783	0.995
Interleukin-13	IL13	285.3	272.3	276.0	289.3	264.6	275.7	277.9	276.5	0.880	0.995
Ephrin-A5	EFNA5	3047.0	2433.4	2583.0	3203.5	2172.8	2650.2	2687.8	2675.5	0.974	0.995
Natural cytotoxicity triggering receptor 1	NCR1	530.5	513.6	535.2	535.9	535.1	501.2	526.4	524.1	0.868	0.996

Platelet factor 4	PF4	98.5	111.2	112.9	105.9	112.3	103.0	107.5	107.1	0.935	0.996
Interleukin-1 receptor type 1	IL1R1	32.8	31.7	32.2	33.3	30.9	32.1	32.2	32.1	0.873	0.996
Fibroblast growth factor receptor 3	FGFR3	199.9	206.1	205.9	194.0	205.3	210.1	204.0	203.1	0.884	0.996
C-C motif chemokine 21	CCL21	39.7	43.8	41.4	40.2	42.6	41.6	41.6	41.5	0.911	0.996
Killer cell lectin-like receptor subfamily F member 1	KLRF1	92.7	97.4	90.2	103.9	89.8	85.6	93.4	93.1	0.959	0.996
Netrin receptor UNC5C	UNC5C	62.3	65.6	71.0	67.7	59.4	71.1	66.3	66.1	0.960	0.996
Lysosomal protective protein	CTSA	168.4	153.4	161.4	172.6	149.7	159.3	161.1	160.5	0.950	0.997
Proprotein convertase subtilisin/kexin type 9	PCSK9	20.5	21.4	20.9	20.1	20.4	22.1	20.9	20.9	0.928	0.997
Toll-like receptor 2	TLR2	280.2	233.4	249.9	241.4	278.2	241.6	254.5	253.7	0.969	0.997
Collagenase 3	MMP13	101.8	104.3	96.4	94.7	102.7	104.2	100.8	100.5	0.940	0.997
Myeloblastin	PRTN3	43.0	43.5	43.6	45.8	40.8	43.2	43.4	43.3	0.951	0.998
C-C motif chemokine 24	CCL24	143.2	129.8	137.9	144.7	131.6	133.8	137.0	136.7	0.964	0.998
Tyrosine-protein phosphatase non-receptor type 11	PTPN11	3217.4	3463.0	3100.4	3845.7	3466.1	2451.1	3260.3	3254.3	0.990	0.998
Interleukin-25	IL25	67.2	56.3	54.2	63.9	55.4	58.1	59.2	59.1	0.984	0.998
Serum amyloid P-component	APCS	280.5	271.7	290.8	263.3	293.7	285.6	281.0	280.9	0.991	1.000
CMRF35-like molecule 6	CD300C	81.7	88.6	84.5	90.0	82.6	82.1	84.9	84.9	0.992	1.000
Platelet-activating factor acetylhydrolase	PLA2G7	292.5	264.1	256.7	292.0	267.9	253.1	271.1	271.0	0.995	1.000
Protein E7_HPV18	Human-virus	445.0	421.1	454.7	449.6	430.1	441.0	440.3	440.2	0.998	1.000

		αHetγWT			αKOγKO			αHetγWT	αKOγKO		Fold (αKOγKO
**TargetFullName**	**EntrezGene**	**#1**	**#2**	**#3**	**#1**	**#2**	**#3**	**Average**	**Average**	**t-test**	**/αHetγWT)**
C-C motif chemokine 15	CCL15	76.7	82.2	75.9	82.1	74.7	78	78.3	78.3	1.000	1.000
Stromelysin-2	MMP10	104.6	118.9	106.7	110.4	125.3	94.7	110.1	110.1	0.995	1.001
Fms-related tyrosine kinase 3 ligand	FLT3LG	222.4	229.6	223.3	231.8	219	225.6	225.1	225.5	0.937	1.002
Immunoglobulin E	IGHE IGK@ IGL@	51.8	52	50.2	50.3	51.6	52.4	51.3	51.4	0.911	1.002
Lysosome membrane protein 2	SCARB2	65	65.6	63.6	68.1	62.4	64.1	64.7	64.9	0.946	1.002
BMP-binding endothelial regulator protein	BMPER	62.7	73	65	69.1	67	65.1	66.9	67.1	0.964	1.002
Cytoplasmic tyrosine-protein kinase BMX	BMX	139.8	146.2	143.8	133.7	149.8	147.4	143.3	143.6	0.950	1.003
Sonic hedgehog protein	SHH	436.4	373	405.7	410.9	425.1	382.3	405.0	406.1	0.964	1.003
Oxidized low-density lipoprotein receptor 1	OLR1	49.7	49.5	48	49.2	48.7	49.7	49.1	49.2	0.841	1.003

Coiled-coil domain-containing protein 80	CCDC80	19353.4	15250.6	16658.3	17584	16189.8	17661.3	17087.4	17145.0	0.968	1.003
Fibronectin	FN1	209279.7	161572.2	199594.7	180997.9	183991.5	207468.6	190148.9	190819.3	0.971	1.004
Ephrin type-A receptor 2	EPHA2	96.3	93.9	96.8	92.6	96.1	99.4	95.7	96.0	0.877	1.004
Acidic leucine-rich nuclear phosphoprotein 32 family member B	ANP32B	95.9	131.9	163.3	137.6	127.2	127.9	130.4	130.9	0.981	1.004
Interleukin-27	IL27 EBI3	226.8	214.3	218.3	233.6	219.8	208.7	219.8	220.7	0.918	1.004
Tumor necrosis factor ligand superfamily member 4	TNFSF4	83.8	78.8	76.9	79.5	75.5	85.5	79.8	80.2	0.930	1.004
Neural cell adhesion molecule L1-like protein	CHL1	71.4	110.5	75	98.7	88.3	71	85.6	86.0	0.982	1.004
C-X-C motif chemokine 11	CXCL11	27	25.5	35.2	29.1	25.2	33.8	29.2	29.4	0.974	1.005
Intercellular adhesion molecule 3	ICAM3	211	219.6	203.9	217.1	209.4	210.9	211.5	212.5	0.862	1.005
Inter-alpha-trypsin inhibitor heavy chain H4	ITIH4	44.7	42.4	41.2	46.8	40.3	41.8	42.8	43.0	0.934	1.005
Ectonucleoside triphosphate diphosphohydrolase 3	ENTPD3	529.7	526.7	532.9	524.6	542.2	530.3	529.8	532.4	0.674	1.005
Histone acetyltransferase type B catalytic subunit	HAT1	70	75.3	78.4	70.5	78.3	76.1	74.6	75.0	0.911	1.005
Serine/threonine-protein kinase PAK 7	PAK7	280.1	241.3	252.3	282.4	250.2	245.3	257.9	259.3	0.936	1.005
Chymase	CMA1	149.4	165.2	156.3	159.7	161.1	152.8	157.0	157.9	0.874	1.006
Vasoactive Intestinal Peptide	VIP	123.4	146.6	144.2	124.1	145.4	147.1	138.1	138.9	0.943	1.006
Kallikrein-7	KLK7	18257.3	13696.6	15614.9	17562.4	13343.8	16953.5	15856.3	15953.2	0.961	1.006
3-hydroxyacyl-CoA dehydrogenase type-2	HSD17B10	176.1	167.3	172.5	163.8	171.7	183.8	172.0	173.1	0.871	1.007
Matrix extracellular phosphoglycoprotein	MEPE	33.4	33.7	34.6	32.3	34.5	35.6	33.9	34.1	0.839	1.007
Transketolase	TKT	28126.5	30359.1	25759	26604.3	33358.2	24906.4	28081.5	28289.6	0.947	1.007
Aggrecan core protein	ACAN	118.2	107.3	111.5	115.4	117.3	107.3	112.3	113.3	0.832	1.009
Transforming growth factor beta receptor type 3	TGFBR3	619	623.5	729.2	666.9	621.3	702.4	657.2	663.5	0.892	1.010
C-X-C motif chemokine 13	CXCL13	61.4	59.1	58.8	62.7	55.7	62.7	59.8	60.4	0.827	1.010
Teratocarcinoma-derived growth factor 1	TDGF1	119.8	120.2	119.3	114.9	123.9	124.4	119.8	121.1	0.715	1.011
Intercellular adhesion molecule 5	ICAM5	666.5	620.8	1139.6	599.7	591.7	1263.7	809.0	818.4	0.975	1.012
Trefoil factor 3	TFF3	126.2	126.5	123.2	114.6	116.7	149.2	125.3	126.8	0.904	1.012
Chromobox protein homolog 5	CBX5	133.3	120.4	123.8	131.8	123.7	126.9	125.8	127.5	0.740	1.013
Thymidine kinase, cytosolic	TK1	237.2	213.3	227.3	251.9	214.6	220.3	225.9	228.9	0.837	1.013
C-reactive protein	CRP	80.2	86.3	76.8	84.6	76	86	81.1	82.2	0.806	1.014

Leukocyte immunoglobulin-like receptor subfamily B member 2	LILRB2	37.7	48.9	39.2	43.8	45.1	38.7	41.9	42.5	0.890	1.014
Angiopoietin-4	ANGPT4	83.1	83.2	78.8	85.1	84.3	79.4	81.7	82.9	0.621	1.015
Fibroblast growth factor 8 isoform B	FGF8	418.1	409.1	419.2	405.5	405.3	454.7	415.5	421.8	0.738	1.015
Annexin A2	ANXA2	3623	3683.2	3738.6	3647.4	3657.8	3911.1	3681.6	3738.8	0.586	1.016
Fibroblast growth factor 19	FGF19	186.9	120.8	125.5	180.3	127.8	132	144.4	146.7	0.937	1.016
SPARC	SPARC	157677.2	111205.8	142089.9	134781.6	136946.5	145912.6	136991.0	139213.6	0.888	1.016
Tumor necrosis factor ligand superfamily member 18	TNFSF18	209	203.9	219.4	207	216.1	219.5	210.8	214.2	0.593	1.016
Brain-derived neurotrophic factor	BDNF	147.4	148.3	138.6	143.6	148.6	149.3	144.8	147.2	0.547	1.017
Dual 3',5'-cyclic-AMP and -GMP phosphodiesterase 11A	PDE11A	618.7	817.1	804.6	659.8	896.2	722.2	746.8	759.4	0.901	1.017
FACT complex subunit SSRP1	SSRP1	47.7	49.3	49.2	48.9	48.6	51.2	48.7	49.6	0.447	1.017
Platelet glycoprotein Ib alpha chain	GP1BA	66.5	62.7	62.7	71.5	58.9	64.8	64.0	65.1	0.797	1.017
Cytokine receptor-like factor 1:Cardiotrophin-like cytokine factor 1 Complex	CRLF1 CLCF1	160.3	137	143	157.4	143.5	147.1	146.8	149.3	0.771	1.017
Tyrosine-protein kinase HCK	HCK	88.8	82.8	82.9	87.9	82	89.3	84.8	86.4	0.628	1.018
X-linked interleukin-1 receptor accessory protein-like 2	IL1RAPL2	222.9	210.4	216.2	220.8	223.8	217	216.5	220.5	0.397	1.019
Plasmin	PLG	154	157.5	155.4	165.3	154.1	156.2	155.6	158.5	0.492	1.019
A disintegrin and metalloproteinase with thrombospondin motifs 1	ADAMTS1	84.2	93.4	88.6	83.9	93.8	93.5	88.7	90.4	0.712	1.019
Cadherin-1	CDH1	224	218.8	215.6	207.4	220.7	242.7	219.5	223.6	0.730	1.019
Proteasome activator complex subunit 3	PSME3	74.8	74.8	72.7	79.3	76.5	70.7	74.1	75.5	0.641	1.019
RNA-binding protein 39	RBM39	140.7	144.1	128.4	155.1	142.6	123.6	137.7	140.4	0.811	1.020
Pituitary adenylate cyclase-activating polypeptide 38	ADCYAP1	127.8	183.1	137.6	166	157.5	133.8	149.5	152.4	0.890	1.020
Calcium-dependent phospholipase A2	PLA2G5	88.9	95.8	94.4	94.1	92.2	98.3	93.0	94.9	0.545	1.020
Lymphotoxin-alpha	LTA	166.3	172.8	174.5	171.9	176.6	175.6	171.2	174.7	0.306	1.020
Kunitz-type protease inhibitor 1	SPINT1	282.2	260.3	266.6	282.6	289.6	253.7	269.7	275.3	0.689	1.021
G2/mitotic-specific cyclin-B1	CCNB1	56.2	72.1	58.5	75.7	58.1	56.9	62.3	63.6	0.877	1.021
Stromelysin-1	MMP3	131.8	122.2	136.5	123.4	130.1	145.3	130.2	132.9	0.741	1.021
Mitogen-activated protein kinase 13	MAPK13	58.6	54.9	55.4	62.4	52.9	57.2	56.3	57.5	0.717	1.021

Endothelial monocyte-activating polypeptide 2	AIMP1	9574.4	8060.4	10362	8370.2	9034.6	11192.8	9332.3	9532.5	0.863	1.021
Mitochondrial glutamate carrier 2	SLC25A18	548.4	542.1	538.4	480	595	589	543.0	554.7	0.784	1.022
Nidogen-2	NID2	12745.1	10847.3	12092.8	11208.5	11246.5	14013.7	11895.1	12156.2	0.824	1.022
Cysteine-rich secretory protein 3	CRISP3	57.2	61.8	54.8	62.2	60.8	54.8	57.9	59.3	0.686	1.023
Nucleoside diphosphate kinase B	NME2	33	28.2	29.3	30.7	30.4	31.5	30.2	30.9	0.681	1.023
Serine/threonine-protein kinase PLK1	PLK1	60.3	59.9	59.4	64.4	60.5	58.9	59.9	61.3	0.483	1.023
Insulin	INS	51.2	43.7	31.9	43	43.3	43.5	42.3	43.3	0.875	1.024
Plasminogen activator inhibitor 1	SERPINE1	39.1	34.1	42.9	39.4	40.1	39.4	38.7	39.6	0.750	1.024
Interleukin-17B	IL17B	64.3	28.5	27.4	57.5	32	33.6	40.1	41.0	0.951	1.024
Alpha-1-antitrypsin	SERPINA1	54.4	77.8	52.7	67.5	64.1	57.8	61.6	63.1	0.874	1.024
MHC class I polypeptide-related sequence B	MICB	42.3	42.4	41.8	42.3	44.8	42.5	42.2	43.2	0.326	1.025
Amyloid beta A4 protein	APP	73.1	73.2	76.3	79.3	76.1	72.7	74.2	76.0	0.459	1.025
Epiregulin	EREG	154.9	154.8	152.9	161.9	155.1	157.1	154.2	158.0	0.190	1.025
Interleukin-6 receptor subunit beta	IL6ST	137.9	136.1	136.3	146.3	135.1	139.1	136.8	140.2	0.409	1.025
CD5 antigen-like	CD5L	55	51.5	53.7	55.1	54.8	54.3	53.4	54.7	0.321	1.025
Tissue Factor	F3	175.7	178.5	177.3	177.2	174.9	192.8	177.2	181.6	0.511	1.025
Mitogen-activated protein kinase 8	MAPK8	373.2	455.6	310.9	320.4	417.8	430.3	379.9	389.5	0.869	1.025
cAMP-dependent protein kinase catalytic subunit alpha	PRKACA	404.4	762.4	444	606.6	668.9	376.1	536.9	550.5	0.930	1.025
NKG2D ligand 2	ULBP2	58	55.4	55	62.5	54.6	55.6	56.1	57.6	0.633	1.026
Protein disulfide-isomerase	P4HB	58.7	58.9	58.6	60.4	59.8	60.6	58.7	60.3	0.015	1.026
Plasminogen	PLG	24.6	22.8	23.8	23.5	25.6	24	23.7	24.4	0.484	1.027
NADPH--cytochrome P450 reductase	POR	73.7	68.7	70	75.4	66.9	76	70.8	72.8	0.593	1.028
Fibroblast growth factor 4	FGF4	72.6	76.1	76.1	72.1	78.6	80.5	74.9	77.1	0.505	1.028
Alkaline phosphatase, tissue-nonspecific isozyme	ALPL	719.2	304	285.6	271.5	265.6	809	436.3	448.7	0.959	1.028
Myeloperoxidase	MPO	100	99.5	87.6	102.7	93.3	99.3	95.7	98.4	0.610	1.029
Interleukin-4 receptor subunit alpha	IL4R	52.1	54.7	49.5	50.6	55	55.3	52.1	53.6	0.512	1.029
Hepatocyte growth factor activator	HGFAC	196137.5	156323.2	181305.2	176590.9	181546.6	191503.5	177922.0	183213.7	0.703	1.030
Tryptase gamma	TPSG1	108.2	90.9	98.4	107	98.3	101.1	99.2	102.1	0.635	1.030
Iduronate 2-sulfatase	IDS	221.2	213.9	206.7	211.4	227.9	221.8	213.9	220.4	0.371	1.030
Placenta growth factor	PGF	116	116.3	117.2	125.2	114.2	120.8	116.5	120.1	0.381	1.031
Angiotensinogen	AGT	33.6	34.8	32	36.4	35	32.1	33.5	34.5	0.536	1.031

Alpha-2-antiplasmin	SERPINF2	40.4	28.2	27.7	43	26.9	29.4	32.1	33.1	0.885	1.031
Annexin A1	ANXA1	445.7	440.5	452.9	377	496	508.2	446.4	460.4	0.770	1.031
Prothrombin	F2	222246.4	174046.2	207772.3	208100.1	197013.7	218111.7	201355.0	207741.8	0.711	1.032
Bone morphogenetic protein 7	BMP7	664.9	503.4	633.1	569.7	530.3	758.9	600.5	619.6	0.836	1.032
Interleukin-20 receptor subunit alpha	IL20RA	31.4	31.7	30.8	32.2	32.7	32.1	31.3	32.3	0.039	1.033
Complement C1r subcomponent	C1R	17.9	16.1	16.7	16.7	17.4	18.3	16.9	17.5	0.466	1.034
Macrophage-stimulating protein receptor	MST1R	236.4	216.9	224.8	255.9	223	222.1	226.0	233.7	0.584	1.034
Activin receptor type-1B	ACVR1B	108.2	182	103.7	110.4	185.4	111.5	131.3	135.8	0.906	1.034
Killer cell immunoglobulin-like receptor 3DL2	KIR3DL2	57.6	60.1	56.9	64.3	58.3	58	58.2	60.2	0.446	1.034
CD70 antigen	CD70	218.4	248.6	235.2	232	254.9	239.8	234.1	242.2	0.502	1.035
Leukemia inhibitory factor receptor	LIFR	35.3	29.5	29.6	33.4	28.6	35.7	31.5	32.6	0.718	1.035
Tumor-associated calcium signal transducer 2	TACSTD2	122.1	133.4	133.2	127.8	139.4	135.2	129.6	134.1	0.417	1.035
Glucose-6-phosphate isomerase	GPI	41.4	34.8	33.7	42.9	35.1	35.8	36.6	37.9	0.726	1.035
[Pyruvate dehydrogenase (acetyl-transferring)] kinase isozyme 1, mitochondrial	PDK1	624.5	574.5	575.1	627.1	597.7	612.7	591.4	612.5	0.339	1.036
Cadherin-2	CDH2	311	302	317.5	309.8	308.6	345.5	310.2	321.3	0.462	1.036
C-type mannose receptor 2	MRC2	43.9	52.2	42.6	51.2	47.9	44.6	46.2	47.9	0.668	1.036
EGF-like module-containing mucin-like hormone receptor-like 2	EMR2	50.4	45.4	45	47.1	47.8	51	46.9	48.6	0.471	1.036
Platelet glycoprotein VI	GP6	104.6	108.1	109.4	109	112.7	112.2	107.4	111.3	0.103	1.037
Aurora kinase A	AURKA	505.5	480.1	490.9	521.4	508.3	501.5	492.2	510.4	0.128	1.037
Persephin	PSPN	69.9	63.3	63.5	69.4	65.3	69.4	65.6	68.0	0.400	1.038
Aromatic-L-amino-acid decarboxylase	DDC	47.6	37	36.5	47.2	39.1	39.4	40.4	41.9	0.751	1.038
Interleukin-6	IL6	70.3	76.1	65.3	77.1	73	69.7	70.6	73.3	0.520	1.038
Gremlin-1	GREM1	78.1	82.8	77.6	83.6	88.3	75.8	79.5	82.6	0.503	1.039
Histidine-rich glycoprotein	HRG	110.6	112	111.5	123.9	110.3	112.8	111.4	115.7	0.412	1.039
Fibrinogen	FGA FGB FGG	208245.4	165637.2	194427.7	187576.5	192637	210089	189436.8	196767.5	0.642	1.039
Superoxide dismutase [Mn], mitochondrial	SOD2	21602	21504.4	23070.3	23992.3	22169	22624.9	22058.9	22928.7	0.309	1.039
Baculoviral IAP repeat-containing protein 3	BIRC3	72.7	65.7	66.6	79.4	66.9	66.8	68.3	71.0	0.607	1.040
A disintegrin and metalloproteinase with thrombospondin motifs 5	ADAMTS5	142.6	132.7	136.6	143.3	134.3	150.6	137.3	142.7	0.392	1.040

Interleukin-12 receptor subunit beta-1	IL12RB1	129.1	123.6	119.4	135.8	126.2	124.9	124.0	129.0	0.331	1.040
Inhibin beta A chain:Inhibin beta B chain heterodimer	INHBA INHBB	39.9	51.3	49	45.5	52.7	47.6	46.7	48.6	0.676	1.040
Cystatin-SN	CST1	106.2	84.4	73.6	78.5	76	120.5	88.1	91.7	0.847	1.041
Asialoglycoprotein receptor 1	ASGR1	57	59.5	55.4	61.5	57.1	60.4	57.3	59.7	0.255	1.041
Interstitial collagenase	MMP1	127.6	120.8	125.8	137.5	124.1	128.1	124.7	129.9	0.331	1.041
Megakaryocyte-associated tyrosine-protein kinase	MATK	104.1	102.4	96.5	100.1	102.2	113.3	101.0	105.2	0.434	1.042
Ephrin-B3	EFNB3	426.6	421.1	382.8	424.8	405.3	452	410.2	427.4	0.424	1.042
Carbonic anhydrase 2	CA2	44.5	46.1	40.3	49.9	43.1	43.4	43.6	45.5	0.552	1.042
Pancreatic hormone	PPY	114.4	124.5	111.5	128.7	121.2	115.3	116.8	121.7	0.423	1.042
N-acylethanolamine-hydrolyzing acid amidase	NAAA	118.4	114.4	124	121.3	118.8	131.9	118.9	124.0	0.365	1.043
Ficolin-3	FCN3	34.3	87.2	30.6	46.3	81.2	31.1	50.7	52.9	0.931	1.043
Insulin-like growth factor-binding protein 6	IGFBP6	143.7	115	112	149.8	117.9	118.9	123.6	128.9	0.734	1.043
Eukaryotic translation initiation factor 4E-binding protein 2	EIF4EBP2	93	94.1	91.4	93.3	93.5	103.7	92.8	96.8	0.364	1.043
Pleiotrophin	PTN	19735.4	15671.5	16571.9	17223.8	16939.8	20056	17326.3	18073.2	0.663	1.043
Scavenger receptor class F member 2	SCARF2	77	80.1	70.1	91.8	75.8	69.6	75.7	79.1	0.679	1.044
Immunoglobulin D	IGHD IGK@ IGL@	42.5	39.9	38	41.5	40.4	43.8	40.1	41.9	0.347	1.044
Protein Rev_HV2BE	Human-virus	30.5	30	27.5	30	29.7	32.2	29.3	30.6	0.347	1.044
Interleukin-17D	IL17D	74.4	84	82	85.6	86.5	79.1	80.1	83.7	0.393	1.045
Matrilin-3	MATN3	149.3	147.5	145.2	158.1	157.7	146.2	147.3	154.0	0.224	1.045
Contactin-5	CNTN5	72.6	68.8	72.6	70.2	82	71.6	71.3	74.6	0.479	1.046
ATP-dependent RNA helicase DDX19B	DDX19B	159	154.8	141.5	172.5	145.8	157.9	151.8	158.7	0.503	1.046
Carboxypeptidase B2	CPB2	61.4	64.1	63.3	63.5	66.8	67.3	62.9	65.9	0.121	1.047
Growth hormone receptor	GHR	123.3	119.5	127.5	135.4	120.7	131.7	123.4	129.3	0.326	1.047
Complement factor B	CFB	37.1	26.1	23.1	36.6	26.9	26.9	28.8	30.1	0.812	1.048
Leucine-rich repeats and immunoglobulin-like domains protein 3	LRIG3	94.9	94.5	98.4	99.4	103.1	99.1	95.9	100.5	0.062	1.048
C-C motif chemokine 14	CCL14	32.6	32.9	30	34.7	32.6	32.8	31.8	33.4	0.255	1.048
Homeodomain-interacting protein kinase 3	HIPK3	438.7	377.2	409.8	438.6	409.4	437.8	408.6	428.6	0.393	1.049
Neuropilin-1	NRP1	2607.2	2331.1	2376.8	2582.5	2584	2508.9	2438.4	2558.5	0.293	1.049
CD209 antigen	CD209	243.9	221.4	207.7	230.7	242.4	233.1	224.3	235.4	0.408	1.049

Leukocyte immunoglobulin-like receptor subfamily B member 1	LILRB1	47.8	64.7	50.8	50.6	65.6	55.2	54.4	57.1	0.714	1.050
Thrombospondin-4	THBS4	8298.1	7383	7923.1	9139.4	6392.8	9246.5	7868.1	8259.6	0.721	1.050
Ciliary Neurotrophic Factor	CNTF	95.6	86.2	87.1	98.5	89.2	94.7	89.6	94.1	0.328	1.050
Complement C3	C3	57	61.5	55.4	58.2	60.1	64.4	58.0	60.9	0.320	1.051
Galactoside 3(4)-L-fucosyltransferase	FUT3	63.2	60	60.5	69.8	62.7	60.5	61.2	64.3	0.388	1.051
Nicotinamide phosphoribosyltransferase	NAMPT	132.8	86.9	85.8	127.3	98.9	94.8	101.8	107.0	0.796	1.051
A disintegrin and metalloproteinase with thrombospondin motifs 4	ADAMTS4	100.8	94.9	97.5	103.2	99.8	105.1	97.7	102.7	0.098	1.051
Carbonic anhydrase 9	CA9	710.2	633.2	683	614.2	674.3	841.8	675.5	710.1	0.669	1.051
Small nuclear ribonucleoprotein F	SNRPF	605.5	677.7	732.7	722.6	738.9	657.9	672.0	706.5	0.486	1.051
3-hydroxyisobutyrate dehydrogenase, mitochondrial	HIBADH	191.6	188.2	188.5	198.7	189.4	209.5	189.4	199.2	0.232	1.052
Tyrosine-protein kinase Fgr	FGR	50.3	60	50.6	52	58	59.2	53.6	56.4	0.520	1.052
Protein FAM107B	FAM107B	159.7	155.5	156.4	162.4	161.3	172.5	157.2	165.4	0.137	1.052
Tumor necrosis factor receptor superfamily member 4	TNFRSF4	56.4	61.6	54.3	63.2	63	55.2	57.4	60.5	0.426	1.053
Mitogen-activated protein kinase 14	MAPK14	1104.9	1265.2	1073.3	1275.3	1275.8	1074.8	1147.8	1208.6	0.534	1.053
6-phosphogluconate dehydrogenase, decarboxylating	PGD	42036.8	45899.4	44175.7	50723.2	46269.7	42224.6	44037.3	46405.8	0.449	1.054
Stem Cell Growth Factor-beta	CLEC11A	121.4	118.2	114.3	124.6	123.9	124.5	118.0	124.3	0.089	1.054
Reticulon-4	RTN4	51.1	56	51.4	56.6	53.4	57.2	52.8	55.7	0.222	1.055
Complement factor H	CFH	66.9	72.1	69.3	68.6	74.7	76.6	69.4	73.3	0.258	1.056
S-formylglutathione hydrolase	ESD	753.5	778	723.2	777.4	946.1	657.2	751.6	793.6	0.668	1.056
T-cell surface glycoprotein CD4	CD4	117.1	109.3	116.5	120.4	117.9	123.8	114.3	120.7	0.112	1.056
Pyruvate kinase PKM	PKM2	159655.4	126351.2	142337.2	146603.4	142287	163476.3	142781.3	150788.9	0.533	1.056
Ephrin-A4	EFNA4	81.6	71.5	78.5	86.1	78.9	79.6	77.2	81.5	0.318	1.056
Tumor necrosis factor ligand superfamily member 9	TNFSF9	93	85.1	85.8	90.6	91.9	96.3	88.0	92.9	0.189	1.056
Apolipoprotein E (isoform E4)	APOE	108.9	107.7	110	118.7	113.6	112.8	108.9	115.0	0.066	1.057
Serine/threonine-protein kinase pim-1	PIM1	179.1	187.1	250	194.9	194.6	261.9	205.4	217.1	0.730	1.057
Immunoglobulin M	IGHM IGJ IGK@ IGL@	161.9	172.4	161.8	178.2	172.4	173.9	165.4	174.8	0.097	1.057
Fibroblast growth factor receptor 1	FGFR1	35241.9	28770.8	30291.7	35898.7	30555.2	33260.7	31434.8	33238.2	0.511	1.057
Lactotransferrin	LTF	158.4	85.9	66.4	72.2	67	189.4	103.6	109.5	0.909	1.058
Metalloproteinase inhibitor 2	TIMP2	92612.1	68125.7	85303	89501.6	84161.2	86605.3	82013.6	86756.0	0.583	1.058

High affinity cGMP-specific 3',5'-cyclic phosphodiesterase 9A	PDE9A	124	122.4	128.2	120.3	138.2	138	124.9	132.2	0.344	1.058
Endothelial cell-selective adhesion molecule	ESAM	241.2	214	226.7	250.9	226.4	244.5	227.3	240.6	0.284	1.059
X-ray repair cross-complementing protein 6	XRCC6	35.7	35.7	32.7	35.6	37.2	37.4	34.7	36.7	0.170	1.059
Fibroblast growth factor 17	FGF17	53.7	48.4	50.3	51.8	54.4	55.2	50.8	53.8	0.193	1.059
Contactin-2	CNTN2	124.9	121.8	115.4	132.3	125.6	125.7	120.7	127.9	0.119	1.059
Receptor tyrosine-protein kinase erbB-2	ERBB2	167.2	141.2	175	163.5	159.2	189.5	161.1	170.7	0.529	1.060
BDNF/NT-3 growth factors receptor	NTRK2	41.2	40.4	39	44.2	41.3	42.3	40.2	42.6	0.093	1.060
C-X-C motif chemokine 6	CXCL6	86.1	75.1	76.1	85.6	79.1	86.8	79.1	83.8	0.335	1.060
Granzyme A	GZMA	76.9	70.1	71.6	81.8	73.7	76.2	72.9	77.2	0.241	1.060
Fibrinogen gamma chain	FGG	201718.6	172902.2	191518.4	194367.5	194898.2	211063.1	188713.1	200109.6	0.330	1.060
Heparan-sulfate 6-O-sulfotransferase 1	HS6ST1	79.7	64.5	75.9	79.2	75	79.2	73.4	77.8	0.438	1.060
Cytotoxic T-lymphocyte protein 4	CTLA4	44.5	42.4	46.7	45.8	44.1	51.8	44.5	47.2	0.381	1.061
Galectin-2	LGALS2	154.3	143.9	143.8	152.1	147.4	169.3	147.3	156.3	0.319	1.061
Interleukin-20	IL20	61.6	57.1	57.4	61.9	61.8	63.1	58.7	62.3	0.124	1.061
Human Chorionic Gonadotropin	CGA CGB	90.2	79.3	83.3	90.7	88.3	89.2	84.3	89.4	0.245	1.061
Collectin-11	COLEC11	13235.2	11891.5	12063.4	9531.5	12990	16935.6	12396.7	13152.4	0.760	1.061
Osteomodulin	OMD	35	39	35.9	40.2	38.4	38	36.6	38.9	0.202	1.061
Somatostatin-28	SST	172.5	168.8	165.1	181.6	171.1	184.7	168.8	179.1	0.112	1.061
Complement C3d fragment	C3	64.4	58.6	58.2	68.1	59.2	65.1	60.4	64.1	0.324	1.062
Intercellular adhesion molecule 2	ICAM2	232	218.9	221.8	222.5	239.6	252.3	224.2	238.1	0.246	1.062
gp41 C34 peptide, HIV	Human-virus	213.7	213.5	217.4	220	211.4	253.3	214.9	228.2	0.405	1.062
Tumor necrosis factor ligand superfamily member 6, soluble form	FASLG	181.2	169.5	173.9	179.8	184.8	192.8	174.9	185.8	0.099	1.063
Tyrosine-protein phosphatase non-receptor type 2	PTPN2	148.7	140.2	142.8	151.1	153.1	154.5	143.9	152.9	0.055	1.063
Complement component C9	C9	36.4	38.2	41.7	42.4	38.4	42.8	38.8	41.2	0.311	1.063
Sialic acid-binding Ig-like lectin 7	SIGLEC7	34.2	36.7	34.1	37.1	37.6	36.9	35.0	37.2	0.115	1.063
Calcineurin	PPP3CA PPP3R1	838.2	1166.3	806.2	1113.6	909.5	964.5	936.9	995.9	0.681	1.063
C-type lectin domain family 1 member B	CLEC1B	36	29.4	31.4	36.4	32	34.5	32.3	34.3	0.440	1.063
Lactoperoxidase	LPO	57.8	64.3	53.8	63.8	57.5	65.7	58.6	62.3	0.403	1.063
Seprase	FAP	70.8	73.2	69.7	77.4	78.1	71.7	71.2	75.7	0.143	1.063

Clusterin	CLU	40.5	40.3	47	46.7	43.4	45.8	42.6	45.3	0.350	1.063
Interleukin-5 receptor subunit alpha	IL5RA	161.3	149.9	155.5	168.9	168.4	159.1	155.6	165.5	0.097	1.064
Low affinity immunoglobulin gamma Fc region receptor III-B	FCGR3B	54.5	58.3	58.4	57.9	60.8	63.5	57.1	60.7	0.154	1.064
Dual specificity mitogen-activated protein kinase kinase 4	MAP2K4	8213.9	7379.2	7526.7	8410.9	8399.2	7799.6	7706.6	8203.2	0.207	1.064
Cerebral dopamine neurotrophic factor	CDNF	95	89.1	88.9	104	88.8	97.9	91.0	96.9	0.317	1.065
Protein NOV homolog	NOV	376	337.4	340.9	448.6	367.8	306.3	351.4	374.2	0.642	1.065
Proprotein convertase subtilisin/kexin type 7	PCSK7	162.4	152.2	157.5	168.5	158.8	175.5	157.4	167.6	0.160	1.065
Dipeptidyl peptidase 1	CTSC	301.3	258	291.6	302.6	289.4	314.3	283.6	302.1	0.302	1.065
Complement C5	C5	49.9	46.6	51.9	57.5	51.7	48.9	49.5	52.7	0.349	1.065
C-C motif chemokine 25	CCL25	84	76.4	81	87.5	80.6	89.1	80.5	85.7	0.200	1.065
Fibronectin Fragment 4	FN1	164676.4	123673	146561.1	146157.7	139699.7	177534.5	144970.2	154464.0	0.599	1.065
Complement C4	C4A C4B	24.5	25.5	27.6	26.6	27.4	28.7	25.9	27.6	0.207	1.066
Protein DJ-1	PARK7	224.6	192	201	253.8	191	213.4	205.9	219.4	0.561	1.066
Granulocyte colony-stimulating factor	CSF3	120	121.6	119.8	126.5	136.8	122	120.5	128.4	0.209	1.066
Aflatoxin B1 aldehyde reductase member 2	AKR7A2	39.1	37.9	34.7	39.8	42.3	37	37.2	39.7	0.290	1.066
Hepatocyte growth factor receptor	MET	80.8	69.6	74.4	78.3	78.1	83.3	74.9	79.9	0.268	1.066
SLAM family member 6	SLAMF6	45.5	48.8	44.1	58.6	44.6	44.4	46.1	49.2	0.587	1.066
Platelet-derived growth factor C	PDGFC	98.3	83.6	142.9	105.8	88.1	152.5	108.3	115.5	0.797	1.067
Cathepsin Z	CTSZ	64.3	71.1	64.1	75.2	72	65.7	66.5	71.0	0.287	1.067
Tumor necrosis factor	TNF	59	56.9	57.8	65.1	64.9	55.4	57.9	61.8	0.347	1.067
Immunoglobulin alpha Fc receptor	FCAR	60.5	59.6	58	64.6	59.7	65.8	59.4	63.4	0.154	1.067
High affinity immunoglobulin gamma Fc receptor I	FCGR1A	130.8	122.3	120.7	124.1	130.2	144.7	124.6	133.0	0.309	1.067
Protein E7_HPV16	Human-virus	38.2	35	36.1	37.1	37.6	42	36.4	38.9	0.261	1.068
Trypsin-3	PRSS3	98.2	93	95.1	103.6	98.3	103.8	95.4	101.9	0.053	1.068
Kunitz-type protease inhibitor 2	SPINT2	107.3	105.9	107	108.9	110.9	122.1	106.7	114.0	0.219	1.068
Neutrophil collagenase	MMP8	12039.6	10037.2	10250.8	11167.7	10885	12505.5	10775.9	11519.4	0.412	1.069
Cystatin-D	CST5	71.4	57.3	55.7	65.2	60.7	71.3	61.5	65.7	0.514	1.069
Heterogeneous nuclear ribonucleoprotein K	HNRNPK	75.2	72	68.8	78.4	74.9	77.8	72.0	77.0	0.094	1.070
Protein kinase C delta type	PRKCD	99	116.5	125.4	94.7	140.1	130.3	113.6	121.7	0.644	1.071
Glutathione S-transferase A3	GSTA3	34.8	62.5	33.6	33.3	42.6	64.4	43.6	46.8	0.824	1.072
Roundabout homolog 2	ROBO2	27290.3	21612.7	23471.3	24487.4	24806.1	28295	24124.8	25862.8	0.452	1.072

Coactosin-like protein	COTL1	1514.1	1393.5	1396.4	1729.8	1665.6	1220.7	1434.7	1538.7	0.586	1.073
VPS10 domain-containing receptor SorCS2	SORCS2	51.3	47.1	50	53.1	51.1	55.1	49.5	53.1	0.099	1.073
Macrophage migration inhibitory factor	MIF	297.2	328.4	339.4	324.4	399.9	313.5	321.7	345.9	0.481	1.075
Serotransferrin	TF	123	89	103.6	125.4	102.1	112	105.2	113.2	0.546	1.076
Sphingosine kinase 2	SPHK2	153.1	141.5	145.1	156.1	155.6	161.9	146.6	157.9	0.060	1.077
Cryptic protein	CFC1	825.2	834.9	793.1	894.1	850	899.3	817.7	881.1	0.037	1.078
72 kDa type IV collagenase	MMP2	11470.7	8972	9689.2	10717	10012.5	11749	10044.0	10826.2	0.439	1.078
Neurogenic locus notch homolog protein 3	NOTCH3	55.5	58.3	53.8	66.6	58	56.1	55.9	60.2	0.310	1.078
Ectonucleotide pyrophosphatase/phosphodiesterase family member 7	ENPP7	58.8	54	54.1	58.8	60.7	60.5	55.6	60.0	0.096	1.078
Cation-independent mannose-6-phosphate receptor	IGF2R	106965.4	79146.3	88476.2	85920.8	82337.4	127946	91529.3	98734.7	0.695	1.079
Glypican-5	GPC5	62.3	57.7	61.6	70.5	60.5	64.9	60.5	65.3	0.239	1.079
A disintegrin and metalloproteinase with thrombospondin motifs 15	ADAMTS15	99.9	89.1	92.8	103.8	100.4	99.8	93.9	101.3	0.132	1.079
Complement C2	C2	41.7	43.1	39.5	48.2	43.5	42.4	41.4	44.7	0.205	1.079
Group IIE secretory phospholipase A2	PLA2G2E	142.7	117.5	128	146	127	145.9	129.4	139.6	0.350	1.079
Diablo homolog, mitochondrial	DIABLO	627.8	586.9	600.9	651.2	681.6	626.4	605.2	653.1	0.080	1.079
Myeloid cell surface antigen CD33	CD33	37.6	31	33.3	35.1	36	38.9	34.0	36.7	0.310	1.079
Thrombopoietin Receptor	MPL	31	32.6	29.3	34	32.7	33.6	31.0	33.4	0.108	1.080
Alpha-(1,3)-fucosyltransferase 5	FUT5	57.2	46.9	50.1	58.5	53.4	54.7	51.4	55.5	0.313	1.080
Mitogen-activated protein kinase 9	MAPK9	1014	774.6	998.3	980.2	979	1054.3	929.0	1004.5	0.436	1.081
Carbohydrate sulfotransferase 6	CHST6	66.5	65.3	63.7	67.1	69.3	75	65.2	70.5	0.142	1.081
Sialic acid-binding Ig-like lectin 9	SIGLEC9	76.9	69.3	74.3	82.7	74.2	81.6	73.5	79.5	0.162	1.082
Caspase-2	CASP2	45.7	44.5	43.9	53.7	48.7	42.8	44.7	48.4	0.361	1.083
Renin	REN	56.5	55.5	53.1	62.2	57	59.7	55.0	59.6	0.073	1.084
Serine/threonine-protein kinase Chk2	CHEK2	103.3	92.3	97.8	108.1	103	106.9	97.8	106.0	0.106	1.084
Protein-tyrosine kinase 6	PTK6	162.6	156.1	171.1	159.6	165.3	206.1	163.3	177.0	0.451	1.084
Proteasome subunit alpha type-1	PSMA1	197.6	223.3	210.8	223.4	262.9	198.8	210.6	228.4	0.449	1.085
T-lymphocyte surface antigen Ly-9	LY9	34.8	31	36.9	37.4	33.8	40.2	34.2	37.1	0.316	1.085
C-C motif chemokine 4-like	CCL4L1	43.9	45.8	44.7	50.6	46.8	48.4	44.8	48.6	0.055	1.085

Granzyme B	GZMB	57.6	58.1	56.3	57.3	63.4	66	57.3	62.2	0.194	1.085
Secretin	SCT	40.5	40.6	37.9	45.5	40.3	43.4	39.7	43.1	0.141	1.086
C-C motif chemokine 22	CCL22	53.8	52.8	49.7	56	54.9	58.8	52.1	56.6	0.058	1.086
Interleukin-17A	IL17A	88.1	78.9	82.7	87.8	88.7	94.7	83.2	90.4	0.108	1.086
Fibroblast growth factor 23	FGF23	67.8	64.8	61.2	77.4	65.8	67.3	64.6	70.2	0.268	1.086
C3a anaphylatoxin des Arginine	C3	67.6	60.3	65.1	66.6	73.4	69.8	64.3	69.9	0.127	1.087
Epidermal growth factor receptor	EGFR	138551.8	104710.8	121421	122574.4	121700.7	152162.4	121561.2	132145.8	0.491	1.087
Leucine-rich repeat transmembrane neuronal protein 1	LRRTM1	23.7	23.9	22.4	25.9	24.6	25.6	23.3	25.4	0.031	1.087
Tumor necrosis factor receptor superfamily member 21	TNFRSF21	79.5	43.9	43.7	87.6	46.6	47.5	55.7	60.6	0.801	1.087
Brain natriuretic peptide 32	NPPB	95.7	113.3	94	109.9	108.8	110.9	101.0	109.9	0.287	1.088
Resistin	RETN	128.5	139	123.3	149.1	137.8	138.3	130.3	141.7	0.128	1.088
Interleukin-17 receptor A	IL17RA	73.5	79.2	78.6	86.6	86.7	78.4	77.1	83.9	0.119	1.088
Prostate-specific antigen	KLK3	44.6	40.4	41.7	46.8	43.2	47.9	42.2	46.0	0.120	1.088
Neurotrophin-4	NTF4	78.5	72	71.9	79.7	78.1	84.3	74.1	80.7	0.086	1.089
Dynein light chain 1, cytoplasmic	DYNLL1	102.6	94.9	119.5	107.7	104.4	133	105.7	115.0	0.466	1.089
Lymphocyte antigen 86	LY86	6474.5	5806.8	6360.1	6370.9	7389.1	6534.8	6213.8	6764.9	0.229	1.089
Sialoadhesin	SIGLEC1	24.4	22.3	21.7	23.2	25.4	25.9	22.8	24.8	0.156	1.089
Complement C4b	C4A C4B	45.9	48.2	48.3	49	51.7	54.4	47.5	51.7	0.095	1.089
interleukin-17 receptor B	IL17RB	120.7	104.7	115.4	128.2	118.8	124.2	113.6	123.7	0.153	1.089
C3a anaphylatoxin	C3	16.3	15.1	13.4	17.2	15.6	16	14.9	16.3	0.258	1.089
Cell adhesion molecule 1	CADM1	491.4	425	434.6	539.9	453.1	478.9	450.3	490.6	0.292	1.089
Contactin-1	CNTN1	4510.4	3814.7	4032.7	4661.2	4204.3	4606.9	4119.3	4490.8	0.221	1.090
Tyrosine-protein kinase Fer	FER	18.9	27.7	20.1	23.3	26.3	23.2	22.2	24.3	0.547	1.091
Metalloproteinase inhibitor 1	TIMP1	35.3	32.6	33.5	38.8	35.1	36.8	33.8	36.9	0.086	1.092
Heparin-binding EGF-like growth factor	HBEGF	86	81	83.4	86.3	90.8	96.4	83.5	91.2	0.101	1.092
Beta-2-microglobulin	B2M	21.3	20.9	20.2	24	21.9	22.4	20.8	22.8	0.071	1.095
Serine/threonine-protein kinase PAK 6	PAK6	228.2	216.8	237.6	224.1	239.2	284.2	227.5	249.2	0.355	1.095
C5a anaphylatoxin	C5	52.5	48.3	49.9	50.8	58.3	56	50.2	55.0	0.151	1.096
E-Selectin	SELE	95791.5	84481.2	90063.8	106297.3	80183.7	109748.8	90112.2	98743.3	0.459	1.096
Glypican-2	GPC2	41.7	38.6	38.5	45.2	41.5	43.5	39.6	43.4	0.064	1.096
Galectin-3-binding protein	LGALS3BP	29.7	26.6	28.1	30	30.3	32.2	28.1	30.8	0.079	1.096
C-C motif chemokine 18	CCL18	37	40.4	35.1	45.9	40.7	36.7	37.5	41.1	0.322	1.096
Endothelial cell-specific molecule 1	ESM1	146	138.3	143.2	166.8	148.4	153.4	142.5	156.2	0.116	1.096
Serine/threonine-protein kinase Chk1	CHEK1	200	199.2	205.1	210	222.2	230.3	201.4	220.8	0.070	1.096

Lymphatic vessel endothelial hyaluronic acid receptor 1	LYVE1	74.1	70.2	72.2	79.9	78.7	78.9	72.2	79.2	0.017	1.097
Scavenger receptor class F member 1	SCARF1	74.2	79.1	72.6	90.8	80.1	77	75.3	82.6	0.215	1.097
Interleukin-24	IL24	82.2	82.4	82.1	87.7	92.9	90.5	82.2	90.4	0.032	1.099
Interferon gamma receptor 1	IFNGR1	64.2	64.5	62.3	74.6	66.9	68.4	63.7	70.0	0.106	1.099
Ephrin type-A receptor 3	EPHA3	62.1	54.9	53.8	61.9	59.6	66.3	56.9	62.6	0.163	1.100
Thrombin	F2	179.9	164.4	155.4	177.4	185.5	186.6	166.6	183.2	0.133	1.100
Glyceraldehyde-3-phosphate dehydrogenase	GAPDH	316	385.9	292.7	297.1	337.7	460.2	331.5	365.0	0.593	1.101
Elafin	PI3	100.1	105.7	94.9	111.4	103.8	116.3	100.2	110.5	0.100	1.102
Plasma kallikrein	KLKB1	26.6	27.7	26.7	30	28.6	30.7	27.0	29.8	0.027	1.102
Thyroglobulin	TG	74.9	72.2	71.9	80.9	74.8	85.9	73.0	80.5	0.134	1.103
Fetuin-B	FETUB	35.2	34.6	35.5	41.3	36.7	38.2	35.1	38.7	0.110	1.104
Macrophage metalloelastase	MMP12	29.8	27.7	29.1	32.5	30.2	32.9	28.9	31.9	0.050	1.104
Calpastatin	CAST	5879.1	5254.8	5448	5848.7	5577.6	6884	5527.3	6103.4	0.286	1.104
Sex hormone-binding globulin	SHBG	34.1	33	31.1	41.4	34.5	32.6	32.7	36.2	0.328	1.105
Carbonic anhydrase 6	CA6	43	36.7	37.2	42.5	41.6	45.1	39.0	43.1	0.170	1.105
Protein S100-A9	S100A9	320.1	338.3	318.2	385.1	354.4	339.9	325.5	359.8	0.107	1.105
C-C motif chemokine 20	CCL20	122.3	119.2	118.2	119.8	129.3	148.5	119.9	132.5	0.272	1.105
Vitamin K-dependent protein S	PROS1	187195.9	152702.6	173636.8	190617.1	182985.5	194282.4	171178.4	189295.0	0.206	1.106
Sialic acid-binding Ig-like lectin 6	SIGLEC6	46.5	43.7	42.4	48.1	48.7	49.9	44.2	48.9	0.044	1.106
Integrin alpha-I: beta-1 complex	ITGA1 ITGB1	11097.1	9023.1	9390.6	10545.5	11992.3	10111.3	9836.9	10883.0	0.289	1.106
Leucine-rich repeat transmembrane neuronal protein 3	LRRTM3	48.1	44.4	45.7	48.1	47.7	57.1	46.1	51.0	0.247	1.106
Serum paraoxonase/arylesterase 1	PON1	64.6	60.3	62.7	71.2	66.5	69.9	62.5	69.2	0.024	1.107
Natural cytotoxicity triggering receptor 2	NCR2	53.1	45.7	48.1	54.9	53.6	54.4	49.0	54.3	0.130	1.109
Mitogen-activated protein kinase 12	MAPK12	70.7	64.4	69.7	73	77.6	76.7	68.3	75.8	0.041	1.110
Hepatocyte growth factor-like protein	MST1	32.5	34.1	34.4	35.6	38.7	37.9	33.7	37.4	0.036	1.111
Carbonic anhydrase 7	CA7	96.1	94.2	88.1	100	96.9	112.5	92.8	103.1	0.150	1.111
E3 ubiquitin-protein ligase Mdm2	MDM2	44.6	39.7	41	50.9	42.9	45.5	41.8	46.4	0.182	1.112
Prokineticin-1	PROK1	35.4	38.1	34.6	39.3	40.6	40.3	36.0	40.1	0.049	1.112
Interleukin-22 receptor subunit alpha-2	IL22RA2	56.7	48.7	50.3	59.2	52.7	61.3	51.9	57.7	0.177	1.112
C-type lectin domain family 4 member K	CD207	39.1	36.1	39.5	44.3	40.8	42.5	38.2	42.5	0.044	1.112

Tumor necrosis factor receptor superfamily member 9	TNFRSF9	85.8	82.1	84.6	87.4	92.5	101.2	84.2	93.7	0.134	1.113
C-C motif chemokine 8	CCL8	98.1	86.3	93.4	101.5	97.3	110.7	92.6	103.2	0.115	1.114
Arylsulfatase B	ARSB	376.1	350	352.4	424.5	382.7	395.7	359.5	401.0	0.058	1.115
Macrophage colony-stimulating factor 1 receptor	CSF1R	36.2	34.6	32.9	42	36.5	37.2	34.6	38.6	0.132	1.116
Platelet glycoprotein 4	CD36	156.2	155.4	154.5	176.7	162.1	181.4	155.4	173.4	0.089	1.116
Receptor tyrosine-protein kinase erbB-3	ERBB3	32.6	32.5	29.3	36.6	32.4	36.4	31.5	35.1	0.107	1.117
Histone H2A.z	H2AFZ	82.9	98.3	92.2	87.8	134.1	83.7	91.1	101.9	0.580	1.118
Receptor tyrosine-protein kinase erbB-4	ERBB4	54.8	50.2	51.5	57.1	53.9	64	52.2	58.3	0.163	1.118
TATA-box-binding protein	TBP	177.3	185.8	180.1	194.8	206.4	206.6	181.1	202.6	0.014	1.119
Lipopolysaccharide-binding protein	LBP	22.8	23.2	21.2	24.7	24.1	26.4	22.4	25.1	0.045	1.119
Interleukin-11 receptor subunit alpha	IL11RA	196.5	183.1	186.1	204.7	198.9	230	188.6	211.2	0.127	1.120
Erythropoietin	EPO	85.5	81.4	79.2	83.5	94.3	97.9	82.0	91.9	0.137	1.120
WAP, kazal, immunoglobulin, kunitz and NTR domain-containing protein 1	WFIKKN1	82.7	74.3	76	81.4	83	97	77.7	87.1	0.188	1.122
Kallikrein-5	KLK5	31.8	28.6	27.9	30.6	33.1	35.5	29.4	33.1	0.124	1.123
Alpha-2-macroglobulin	A2M	51.2	49.9	46.6	52.9	53.6	59.6	49.2	55.4	0.083	1.125
Interleukin-10	IL10	40.1	38.9	35.8	43.7	38.1	47.4	38.3	43.1	0.211	1.125
Tyrosine-protein kinase Lyn	LYN	189.1	213.6	184.6	246.4	244.8	169.9	195.8	220.4	0.438	1.126
Desmocollin-3	DSC3	48.9	45.3	45.8	57.5	49.2	51	46.7	52.6	0.130	1.126
Cytochrome c	CYCS	2925.4	2309.7	2840	3159.6	3583.1	2355.4	2691.7	3032.7	0.464	1.127
Enteropeptidase	TMPRSS15	66.5	65.2	62.7	76.1	69.1	74	64.8	73.1	0.038	1.128
Transforming growth factor beta-2	TGFB2	83.6	80.2	80.5	89.5	87	99.1	81.4	91.9	0.095	1.128
Interleukin-6 receptor subunit alpha	IL6R	71.6	74	71.2	81.5	83.2	80	72.3	81.6	0.002	1.129
Cathepsin L2	CTSV	84	80.6	81.7	90.9	85.2	101.9	82.1	92.7	0.159	1.129
Kallikrein-13	KLK13	657.7	608	656.7	655.9	751.6	763	640.8	723.5	0.120	1.129
Peptidoglycan recognition protein 1	PGLYRP1	32.5	29	28.3	34.2	31.4	35.8	29.9	33.8	0.102	1.129
C-C motif chemokine 16	CCL16	23.2	27.7	21.2	27.9	27.5	26.3	24.0	27.2	0.234	1.133
Lymphotactin	XCL1	74.5	68.9	71.1	89.1	75.5	78.5	71.5	81.0	0.134	1.133
Cystatin-C	CST3	58.3	31.9	38.6	59.3	41.2	45.6	42.9	48.7	0.585	1.134
COMM domain-containing protein 7	COMMD7	640.1	670	703.5	718.7	899.7	667.7	671.2	762.0	0.325	1.135

Brain-specific serine protease 4	PRSS22	37.3	35.8	35.7	44.8	39.6	39.2	36.3	41.2	0.102	1.136
Homeobox protein NANOG	NANOG	44.2	40.4	41	43.8	45.5	53.4	41.9	47.6	0.185	1.136
Tumor necrosis factor ligand superfamily member 13B	TNFSF13B	91.1	91.8	88.1	99.8	108.1	100.3	90.3	102.7	0.030	1.137
Granzyme H	GZMH	49.6	50.5	47.1	60	53.8	53.7	49.1	55.8	0.064	1.138
Hemopexin	HPX	16.1	13	16.1	18.5	15.9	17.1	15.1	17.2	0.182	1.139
cGMP-specific 3',5'-cyclic phosphodiesterase	PDE5A	1803.1	2303.2	1856.7	2264.2	2931.9	1610.6	1987.7	2268.9	0.550	1.141
Ligand-dependent nuclear receptor corepressor-like protein	LCORL	43.4	40.4	38.6	49.5	44.6	45.8	40.8	46.6	0.046	1.143
Proliferating cell nuclear antigen	PCNA	36.2	33	39.2	42.5	36	45.5	36.1	41.3	0.205	1.144
Heat shock cognate 71 kDa protein	HSPA8	29	23.8	26.7	30.1	27.9	33	26.5	30.3	0.143	1.145
Antileukoproteinase	SLPI	120.5	95.8	88.3	109	104.4	135.5	101.5	116.3	0.343	1.145
ICOS ligand	ICOSLG	172.7	174.6	159	197.8	207.3	175.4	168.8	193.5	0.103	1.147
C-C motif chemokine 19	CCL19	52.1	46.4	47.9	58.8	52.8	56.3	48.8	56.0	0.042	1.147
Thrombospondin-2	THBS2	21785.3	16827.2	19484.2	23608.8	20139	22908.6	19365.6	22218.8	0.191	1.147
Properdin	CFP	52.7	54.5	53.6	56.8	73.4	54.3	53.6	61.5	0.318	1.147
Triosephosphate isomerase	TPI1	8314.1	10152.1	8205.3	9899.5	12084.6	8620.4	8890.5	10201.5	0.344	1.147
Interleukin-23	IL12B IL23A	34.2	31.3	32.5	38.6	37.3	36.6	32.7	37.5	0.012	1.148
Interferon lambda-2	IFNL2	69.3	64.8	67.3	77.1	75	79.1	67.1	77.1	0.005	1.148
Thioredoxin domain-containing protein 12	TXNDC12	779.6	676.8	744.2	827.2	871	829.2	733.5	842.5	0.050	1.149
Granulins	GRN	84.8	85	81.6	92.1	76.1	120.9	83.8	96.4	0.439	1.150
Tyrosine-protein kinase receptor Tie-1, soluble	TIE1	126.6	123	113.2	137.9	136.9	143.6	120.9	139.5	0.026	1.153
Spectrin alpha chain, non-erythrocytic 1	SPTAN1	34.3	39.5	35	48.9	38.6	38	36.3	41.8	0.254	1.153
Semaphorin-6A	SEMA6A	9856.1	7841.8	8165.4	10179.3	9380.1	10313.3	8621.1	9957.6	0.153	1.155
ADP-ribosyl cyclase/cyclic ADP-ribose hydrolase 2	BST1	84.6	72.6	80.7	92.8	86.4	95.6	79.3	91.6	0.055	1.155
Pappalysin-1	PAPPA	45.5	43	44	52.3	50.1	50.8	44.2	51.1	0.002	1.156
Polymeric immunoglobulin receptor	PIGR	74.6	68	65.7	64.8	66.4	110.1	69.4	80.4	0.538	1.158
Granulocyte-macrophage colony-stimulating factor	CSF2	166.8	174.9	197.6	183	221.6	220.6	179.8	208.4	0.149	1.159
Moesin	MSN	135.6	137.7	132.3	171.6	153.1	145.6	135.2	156.8	0.103	1.160
Trypsin-1	PRSS1	63	62.1	60.1	68.4	67.2	79.3	61.7	71.6	0.118	1.160
Neural cell adhesion molecule L1	L1CAM	17.4	14.3	14.7	18.2	16.8	19	15.5	18.0	0.106	1.164
Phosphoglycerate kinase 1	PGK1	1736.7	1775.6	1730.7	1870.1	2402.2	1835	1747.7	2035.8	0.257	1.165

CD166 antigen	ALCAM	7781.2	6514.2	6915.2	8322.9	8078.5	8361.2	7070.2	8254.2	0.080	1.167
Lymphotoxin alpha1:beta2	LTA LTB	17.7	14.8	14.5	17.3	18.6	19	15.7	18.3	0.106	1.168
Xaa-Pro aminopeptidase 1	XPNPEP1	3807.6	7393.3	5423.5	5715.4	8724.3	5018.4	5541.5	6486.0	0.573	1.170
Lysozyme C	LYZ	812.1	912.5	1010.6	1119.1	883.9	1199.1	911.7	1067.4	0.246	1.171
Scavenger receptor cysteine-rich type 1 protein M130	CD163	46.6	44.2	44.5	60	49.8	48.7	45.1	52.8	0.160	1.171
Neurexin-1-beta	NRXN1	218.5	715.5	199.8	299.5	765.4	264.6	377.9	443.2	0.794	1.173
Importin subunit alpha-1	KPNA2	115.1	107.3	102.2	134.1	124.5	123.3	108.2	127.3	0.020	1.177
Connective tissue-activating peptide III	PPBP	33	31.9	31.8	35.3	38.5	40.1	32.2	38.0	0.047	1.178
Interleukin-7	IL7	22.6	22.8	21.6	28.6	23.1	27.3	22.3	26.3	0.131	1.179
Plasma protease C1 inhibitor	SERPING1	60.9	58.8	58.9	67.4	76.7	66.5	59.5	70.2	0.076	1.179
Cystatin-M	CST6	44	35.9	36.4	45.3	36.4	55.6	38.8	45.8	0.341	1.181
Nidogen-1	NID1	16382.6	13564.2	13344.2	17906.4	15891.2	17331.3	14430.3	17043.0	0.099	1.181
Phosphatidylethanolamine-binding protein 1	PEBP1	32.7	29.1	32	38.2	33.1	39.5	31.3	36.9	0.082	1.181
Complement C3b	C3	17.5	16.7	15.8	19.2	19.4	20.5	16.7	19.7	0.010	1.182
14-3-3 protein family	YWHAB,YWHAE,YWHAG,YWHAH,YWHAQ,YWHAZ,SFN	16556.7	15528.5	15009.7	20512.1	18077.5	17151.2	15698.3	18580.3	0.085	1.184
26S proteasome non-ATPase regulatory subunit 7	PSMD7	166.1	141.5	150.9	174.6	186.7	181.9	152.8	181.1	0.040	1.185
Lactadherin	MFGE8	20.4	19.8	17.2	23.1	21.8	23.2	19.1	22.7	0.050	1.186
Spondin-1	SPON1	3049.8	2562.3	2711	3361.1	3020	3517.2	2774.4	3299.4	0.063	1.189
15-hydroxyprostaglandin dehydrogenase [NAD(+)]	HPGD	1929	2126.8	2178.9	2301	2929.7	2193.7	2078.2	2474.8	0.220	1.191
Mannan-binding lectin serine protease 1	MASP1	3305.1	3088.2	3039.6	3761.3	3599.7	3885.3	3144.3	3748.8	0.007	1.192
Vascular endothelial growth factor A	VEGFA	4202.4	3497.3	4006.3	4612.2	3809.2	5592.6	3902.0	4671.3	0.272	1.197
P-Selectin	SELP	33.1	31.5	32.3	43	36.8	36.3	32.3	38.7	0.091	1.198
Interleukin-1 receptor-like 1	IL1RL1	15.9	16.3	15.5	20.2	17.7	19.3	15.9	19.1	0.039	1.199
Interleukin-19	IL19	437.2	398.2	368.2	475.7	471.8	498.7	401.2	482.1	0.040	1.202
Serum albumin	ALB	29.3	41.6	29.4	36.3	34.2	50.3	33.4	40.3	0.355	1.204
A disintegrin and metalloproteinase with thrombospondin motifs 13	ADAMTS13	18.3	19.4	17.2	20.4	21.1	24.7	18.3	22.1	0.088	1.206
Netrin receptor UNC5D	UNC5D	65.1	41	49.5	73.6	54.8	59.3	51.9	62.6	0.305	1.206

MHC class I polypeptide-related sequence A	MICA	18.8	18.7	18.1	21.4	22.6	23.1	18.5	22.4	0.008	1.207
Tyrosine-protein phosphatase non-receptor type 6	PTPN6	176.5	188.8	182.4	238.1	222.3	202.6	182.6	221.0	0.052	1.211
Ephrin type-A receptor 10	EPHA10	121	110.6	113.6	147.9	113.6	156.8	115.1	139.4	0.201	1.212
Endostatin	COL18A1	7867.4	6312.4	6066.8	10135.5	6567.1	7868.5	6748.9	8190.4	0.309	1.214
Galectin-3	LGALS3	12411.2	10213.9	10627.7	14354.6	11967.5	14105.8	11084.3	13476.0	0.079	1.216
Collagen alpha-1(XXIII) chain	COL23A1	59.4	56.5	55.6	73.7	71.5	63.4	57.2	69.5	0.045	1.216
Baculoviral IAP repeat-containing protein 7 Isoform beta	BIRC7	28.4	27.2	26.9	34	31.5	34.9	27.5	33.5	0.015	1.217
Neuroblastoma suppressor of tumorigenicity 1	NBL1	398.1	295.2	307.8	542.7	323.7	360.8	333.7	409.1	0.392	1.226
Tyrosine-protein kinase Lyn, isoform B	LYN	37.9	37.2	35.5	46.9	44.9	43.9	36.9	45.2	0.002	1.227
Aminoacylase-1	ACY1	3559	3045.7	3019.7	4049.1	4703	3068.9	3208.1	3940.3	0.259	1.228
C-C motif chemokine 7	CCL7	1126.2	754.5	801.2	1310	1022.8	980.1	894.0	1104.3	0.250	1.235
Macrophage-capping protein	CAPG	419.5	236.1	366.7	452.6	310.7	501.1	340.8	421.5	0.365	1.237
Annexin A6	ANXA6	747.7	993.9	825.1	1073.4	1148.9	970.2	855.6	1064.2	0.087	1.244
Vascular endothelial growth factor A, isoform 121	VEGFA	105.1	99.7	96.7	125.1	113.9	136.5	100.5	125.2	0.049	1.245
Tumor necrosis factor receptor superfamily member 19	TNFRSF19	251.7	210.8	225.1	330.4	232	295.6	229.2	286.0	0.177	1.248
Tumor necrosis factor receptor superfamily member EDAR	EDAR	287.9	282	295.7	378.9	343.9	360.4	288.5	361.1	0.011	1.251
Dual specificity mitogen-activated protein kinase kinase 2	MAP2K2	229.5	247.4	220.4	277.5	359.6	236.5	232.4	291.2	0.243	1.253
Chloride intracellular channel protein 1	CLIC1	541.4	595.3	642.1	745.1	786	700.2	592.9	743.8	0.018	1.254
Neutrophil gelatinase-associated lipocalin	LCN2	57.3	55.1	50.4	62.1	59.2	83.3	54.3	68.2	0.202	1.257
NudC domain-containing protein 3	NUDCD3	35.8	35.9	34.4	47.3	46.9	40.5	35.4	44.9	0.044	1.270
Phosphoglycerate mutase 1	PGAM1	113.6	95	92.5	142.9	104.6	135.2	100.4	127.6	0.132	1.271
Proteasome activator complex subunit 1	PSME1	717.7	733.2	688.2	817	1345.7	558	713.0	906.9	0.491	1.272
Stem Cell Growth Factor-alpha	CLEC11A	7532.7	6348.8	7816.2	8743.5	8060.3	10921.8	7232.6	9241.9	0.130	1.278
Periostin	POSTN	325.3	292.2	315.5	392.3	342.6	465	311.0	400.0	0.121	1.286
Protein Wnt-7a	WNT7A	13077.4	10630.3	10883.3	18844.5	13222.1	12491.5	11530.3	14852.7	0.234	1.288
Testican-1	SPOCK1	3494.4	3261	3410.8	4133.6	4654.3	4330.5	3388.7	4372.8	0.012	1.290
Ciliary neurotrophic factor receptor subunit alpha	CNTFR	993.1	884.1	852	1390.8	1049.5	1125.6	909.7	1188.6	0.099	1.307
Fibroblast growth factor 12	FGF12	43.5	42.7	40.8	51.2	52.4	62.6	42.3	55.4	0.063	1.309

Ribosomal protein S6 kinase alpha-3	RPS6KA3	306.7	396.8	288.7	446.5	511.8	345.4	330.7	434.6	0.161	1.314
Integrin alpha-V: beta-5 complex	ITGAV ITGB5	8060.2	4181.6	6389.6	6293.2	7369.9	10849.1	6210.5	8170.7	0.334	1.316
Bcl-2-related protein A1	BCL2A1	1068.6	1052.6	1024.1	1384.4	1523.4	1232.5	1048.4	1380.1	0.055	1.316
14-3-3 protein sigma	SFN	6187.4	3284.9	6179.2	6854.9	5480.8	8434.9	5217.2	6923.5	0.257	1.327
Legumain	LGMN	9020.1	9715.5	9973.3	12316.7	15610.7	10179	9569.6	12702.1	0.182	1.327
Growth/differentiation factor 9	GDF9	308.3	291	297.5	385.6	383.1	425.8	298.9	398.2	0.011	1.332
Interleukin-2	IL2	1948.3	1676.4	1843	2485.9	1890.4	2932.7	1822.6	2436.3	0.173	1.337
Serine protease HTRA2, mitochondrial	HTRA2	467.4	533.9	421.2	665.5	815.1	428.2	474.2	636.3	0.284	1.342
Interleukin-1 alpha	IL1A	2731.4	2266.8	2487	3145.8	3583.2	3317.9	2495.1	3349.0	0.010	1.342
Melanoma-derived growth regulatory protein	MIA	3475.3	2888.1	3106.6	4758.8	3596.1	4542.4	3156.7	4299.1	0.067	1.362
Neural cell adhesion molecule 1, 120 kDa isoform	NCAM1	58.7	78.7	72.2	59.1	168.4	60.8	69.9	96.1	0.545	1.375
Adenylosuccinate lyase	ADSL	194.8	194.5	212.3	261.4	300.4	268.2	200.5	276.7	0.012	1.380
Brother of CDO	BOC	5334.3	3825.5	4723.3	7000.8	6267	6170.6	4627.7	6479.5	0.031	1.400
Caspase-10	CASP10	7512.7	6468.7	7156.6	9428.9	10130	10136.6	7046.0	9898.5	0.002	1.405
Ras-related C3 botulinum toxin substrate 1	RAC1	15513.8	21133.2	18394	27295	28189.4	22098.7	18347.0	25861.0	0.041	1.410
Cathepsin B	CTSB	416.7	356.1	372.8	447.7	619.6	564.1	381.9	543.8	0.072	1.424
Tumor necrosis factor receptor superfamily member 27	EDA2R	592.4	561.1	561.9	839.7	761.4	851.6	571.8	817.6	0.007	1.430
Tumor necrosis factor-inducible gene 6 protein	TNFAIP6	63	56.1	60.6	88.3	93	76.8	59.9	86.0	0.020	1.436
Proteasome subunit alpha type-2	PSMA2	98.3	101.1	104.7	158.8	148	132.4	101.4	146.4	0.023	1.444
Macrophage mannose receptor 1	MRC1	11890.9	10861.4	9494.5	16115.3	18102.2	14367.8	10748.9	16195.1	0.018	1.507
Carbohydrate sulfotransferase 15	CHST15	20965.1	17680.5	17591.8	28672.1	25333	31040.1	18745.8	28348.4	0.012	1.512
Follistatin-related protein 3	FSTL3	13398.3	10132.6	11357.2	20231	14381	18352	11629.4	17654.7	0.052	1.518
Eukaryotic translation initiation factor 4 gamma 2	EIF4G2	278.6	351.2	286.2	444.6	599.5	348.7	305.3	464.3	0.152	1.521
Interleukin-18-binding protein	IL18BP	12916	11683.8	14529.8	13125.1	32393.2	14119.4	13043.2	19879.2	0.389	1.524
NT-3 growth factor receptor	NTRK3	913.9	851.7	775.1	1391.8	1224.5	1283.7	846.9	1300.0	0.002	1.535
Glucokinase regulatory protein	GCKR	241.5	188.6	162	257.6	446.8	207.4	197.4	303.9	0.279	1.540
Glial fibrillary acidic protein	GFAP	448.4	424.2	388.3	692.1	622.3	634.3	420.3	649.6	0.001	1.545
Carbonic anhydrase 3	CA3	1543.1	618.5	460.9	2066.9	1013.8	1009	874.2	1363.2	0.373	1.559
Cell adhesion molecule-related/down-regulated by oncogenes	CDON	53.9	65.2	58.6	66.9	152.2	58.5	59.2	92.5	0.382	1.562
Sorting nexin-4	SNX4	263.9	247.5	233.9	401.3	376.2	397.2	248.4	391.6	0.0003	1.576

Atrial natriuretic factor	NPPA	3748.2	2829.5	2331.8	3842.4	3973.1	6348.2	2969.8	4721.2	0.152	1.590
Antithrombin-III	SERPINC1	77501.5	57791.1	77095.7	116339.7	100274.8	124653.9	70796.1	113756.1	0.012	1.607
Thymidylate synthase	TYMS	314.4	199	304	527.5	340.9	467.8	272.5	445.4	0.068	1.635
Agouti-related protein	AGRP	260.3	229.2	244.5	473.3	348.7	383.1	244.7	401.7	0.045	1.642
Insulin-like growth factor 1 receptor	IGF1R	2063.6	1726.7	1792	3207.8	2779.3	3195.2	1860.8	3060.8	0.003	1.645
Insulin receptor	INSR	955	813.4	778	1377.9	1400.8	1410	848.8	1396.2	0.008	1.645
Angiopoietin-2	ANGPT2	119.6	111.2	107.7	196.6	197.4	164.2	112.8	186.1	0.015	1.649
Angiopoietin-related protein 3	ANGPTL3	58449.3	47212.2	51275.1	86120.6	78408.9	96159.2	52312.2	86896.2	0.008	1.661
Trypsin-2	PRSS2	157.3	166.9	117.4	270.5	240.3	225	147.2	245.3	0.009	1.666
SPARC-like protein 1	SPARCL1	66241	50782.9	56343.3	106329.4	91602.2	96343.2	57789.1	98091.6	0.003	1.697
von Willebrand factor	VWF	47989.2	46539.8	45649.1	72161.1	81840.2	84740.6	46726.0	79580.6	0.011	1.703
Olfactomedin-4	OLFM4	46.8	62.3	45.7	51	158.3	55.1	51.6	88.1	0.408	1.708
Thyroid Stimulating Hormone	CGA TSHB	80.3	52.8	51.2	164.7	77.5	86.9	61.4	109.7	0.216	1.786
Neurexin-3-beta	NRXN3	613.9	475.6	528.6	1032.1	880.3	987.7	539.4	966.7	0.002	1.792
Glutathione S-transferase P	GSTP1	1070.6	388.3	244.9	714.6	1940.6	426.6	567.9	1027.3	0.448	1.809
Protein-glutamine gamma-glutamyltransferase E	TGM3	129.2	124.5	129.5	238	101.6	356.3	127.7	232.0	0.292	1.816
Neuroligin-4, X-linked	NLGN4X	615.3	450.4	656.3	1053.2	867.2	1428.9	574.0	1116.4	0.067	1.945
Heme oxygenase 2	HMOX2	198	139.4	135.7	290.8	377.8	259.9	157.7	309.5	0.030	1.963
Stanniocalcin-1	STC1	10585.7	7683.7	8998.7	17194.9	15341.4	21352	9089.4	17962.8	0.023	1.976
Dual specificity protein phosphatase 3	DUSP3	365.5	391.3	329.6	855.4	732.6	623.4	362.1	737.1	0.024	2.036
Mitochondrial import inner membrane translocase subunit TIM14	DNAJC19	132.3	134.8	134.1	191.2	557	139.9	133.7	296.0	0.342	2.214
Tumor necrosis factor receptor superfamily member 12A	TNFRSF12A/ TWEAKR	334.8	250.9	247.3	808.5	618.4	491.3	277.7	639.4	0.049	2.303
WAP, Kazal, immunoglobulin, Kunitz and NTR domain-containing protein 2	WFIKKN2/GASP2	1921.4	1530.5	1837.7	4562.2	3457.2	4539.7	1763.2	4186.4	0.015	2.374
Heat shock 70 kDa protein 1A/1B	HSPA1A	1873.1	1105.5	1482	3345.3	3760.5	3581.1	1486.9	3562.3	0.003	2.396
Peptide YY	PYY	462.9	471.5	461.4	1091.9	1054.1	1221	465.3	1122.3	0.006	2.412
Growth/differentiation factor 2/BMP9	GDF2/BMP9	1410.6	990.8	826.3	4269.6	2071.4	1745.5	1075.9	2695.5	0.173	2.505
Insulin-like growth factor-binding protein 2	IGFBP2	11210.1	9592.3	10057	27217.8	17678.4	35037.5	10286.5	26644.6	0.081	2.590
Myoglobin	MB	384.1	145.7	105.4	511.8	471.9	810.3	211.7	598.0	0.051	2.824
Glucagon	GCG	178.2	196.3	177	788.2	428.1	463	183.8	559.8	0.081	3.045
Muellerian-inhibiting factor	AMH	1419.7	2428.2	1306.8	1742	3872.7	11434.4	1718.2	5683.0	0.309	3.307

Glypican-3	GPC3	1618.4	1104.1	1246.7	5245.7	4879.4	5428.6	1323.1	5184.6	0.0001	3.919
alpha-1-antichymotrypsin complex	SERPINA3	31.1	28.1	31.1	32.9	33.9	378.7	30.1	148.5	0.412	4.934
60 kDa heat shock protein, mitochondrial	HSPD1	1935.7	2295.6	1945.9	7875.9	23162.8	2815.4	2059.1	11284.7	0.270	5.480
Insulin-like growth factor-binding protein 1	IGFBP1	2138.8	1553.4	730.9	33004.5	32952.6	45645.8	1474.4	37201.0	0.013	25.232

Appendix Table S2. Genes encoding secreted proteins with altered expression in 16-day-old αKOγKO mouse hearts compared to littermate control mouse hearts by RNA-Seq (3 mice per sequencing sample, total of 6 mice per genotype). The proteins are ordered based on fold change. The proteins labeled in blue fonts were tested in Fig 3A based on their selective cardiac expression.

Gene ID	Symbol	αHetγWT RPKM		αHetγKO RPKM		αKOγWT RPKM		αKOγKO RPKM		Fold (αKOγKO /αHetγWT)	P-value	FDR
		#1	#2	#1	#2	#1	#2	#1	#2			
23886	Gdf15	0.4748715	0.997886	1.3095189	0.90588	1.3821645	1.442862	52.784284	19.685112	49.2	2E-173	5E-172
21828	Thbs4	2.4328687	3.296331	6.0846005	6.672508	7.1481602	5.067814	80.599456	71.382332	26.5	0	0
18158	Nppb	74.361504	79.00695	112.34144	121.902	98.99381	206.3011	1952.2222	1393.3965	21.8	0	0
22403	Wisp2	1.2888254	1.359669	1.2930031	1.317705	1.2865563	1.15069	43.951836	11.670889	21.0	2E-118	4E-117
14219	Ctgf	46.579672	60.59649	61.670973	76.32406	57.915667	69.75104	749.12833	650.98171	13.1	0	0
68588	Cthrc1	1.7394549	1.295392	1.493909	1.968447	3.1220728	1.651626	27.425871	9.4353384	12.1	3E-48	2E-47
338417	Scgb1c1	3.3480373	4.50621	7.2392293	5.410324	5.6546353	4.768481	30.361942	41.331863	9.1	7E-119	1E-117
230899	Nppa	2313.1298	2621.314	3367.0254	3175.973	2304.1302	4241.753	22181.396	20368.714	8.6	0	0
19215	Ptgds	16.221988	13.78672	24.39247	30.79052	34.686119	36.91037	118.38455	132.77958	8.4	0	0
20319	Sfrp2	6.0196907	7.437124	6.4733163	7.182672	6.9140704	7.327876	71.378339	33.653565	7.8	2E-293	8E-292
16007	Cyr61	14.366596	16.68046	15.259123	17.29943	14.995867	15.23186	68.57925	173.12288	7.8	0	0
21825	Thbs1	5.2286394	3.92648	3.5903884	4.29786	4.5163257	7.564889	17.176203	30.610852	5.2	1E-271	4E-270
77794	Adamtsl2	19.03228	23.75014	26.019397	26.93898	25.948815	20.96592	142.43255	64.987238	4.8	0	0
67177	Cdt1	31.305857	25.9891	28.969764	28.84586	24.363905	40.06579	133.65543	110.20554	4.3	5E-241	2E-239
21809	Tgfb3	9.8482108	10.63078	12.500611	12.66916	11.079037	13.58571	54.54594	27.524886	4.0	3E-207	9E-206
14314	Fstl1	95.65887	92.46069	97.810682	118.4005	122.69809	107.1257	418.58811	215.07455	3.4	0	0
20720	Serpine2	18.989725	19.13562	22.156414	24.23935	28.168398	31.67206	62.327611	61.08442	3.2	4E-268	1E-266
50781	Dkk3	39.449144	41.66463	46.51386	47.15701	44.868508	59.43272	115.96042	121.9906	2.9	0	0
21857	Timp1	4.8158094	6.012509	6.1172618	5.885596	6.3051527	6.732955	20.654534	10.84075	2.9	1E-16	5E-16
68797	Pdgfrl	7.5298333	8.359186	9.3026695	11.01675	10.502204	10.46082	27.837356	18.362325	2.9	5E-52	5E-51
15200	Hbegf	9.0384409	9.907712	10.147895	9.703299	11.127983	9.440277	31.519358	22.729357	2.9	3E-103	4E-102
12759	Clu	156.70592	152.5458	159.14623	161.5556	123.2628	136.7365	412.15012	390.77409	2.6	0	0
12154	Bmp10	49.534809	50.72414	59.22744	49.63983	31.393906	49.73052	125.94341	125.52264	2.5	1E-220	5E-219
18188	Nrtn	17.510922	24.22381	33.267796	33.94542	37.424718	31.31054	40.634166	55.890149	2.3	3E-151	6E-150
20377	Sfrp1	7.2559878	7.625291	8.368658	9.159584	9.3186335	8.634294	17.32039	16.796048	2.3	1E-120	2E-119
20347	Sema3b	6.437754	6.684743	7.0868546	5.924346	5.6907788	5.994387	16.317912	13.754031	2.3	2E-64	3E-63
22339	Vegfa	153.1366	137.4003	121.68159	124.7486	129.97455	122.1809	85.475104	75.049859	0.6	0	0
22340	Vegfb	139.70188	149.1219	134.92534	134.3411	146.86478	150.2959	55.301087	61.706414	0.4	1E-290	4E-289
239126	C1qtnf9	37.256811	35.70728	37.800366	37.13734	34.658747	27.70463	13.563144	15.45212	0.4	5E-129	9E-128
54612	Sfrp5	3.8652209	3.019122	3.7542527	2.270912	2.5670184	4.3389	0.7435759	1.6351465	0.3	2E-14	9E-14
233813	Vwa3a	5.2057406	4.9079	4.522291	4.800111	3.8811038	4.494185	1.1995982	1.9895772	0.3	6E-51	6E-50
17329	Cxcl9	1.8664876	1.083232	1.7591279	0.952627	0.8998267	0.706654	0.4115116	0.3940112	0.3	1E-23	6E-23
22370	Vtn	19.134422	19.91285	16.405382	17.31733	16.020371	11.57004	2.6198243	4.0963681	0.2	4E-137	8E-136

Appendix Table S3. Sequences of qPCR primers used.

Mouse primer	Sequence
Esrra For	CTCAGCTCTCTACCCAAACGC
Esrra Rev	CCGCTTGGTGATCTCACACTC
Esrrg For	GAATCTTTTTCCCTGCACTACGA
Esrrg Rev	GCTGGAATCAATGTGTCGATCTT
Ghrh For	GGTGCTCTTTGTGATCCTCATC
Ghrh Rev	GTTTCCTGTAGTTGGTGGTGAAG
Gh For	GCTACAGACTCTCGGACCTC
Gh Rev	CGGAGCACAGCATTAGAAAACAG
Npy For	ATGCTAGGTAACAAGCGAATGG
Npy Rev	TGTCGCAGAGCGGAGTAGTAT
Pomc For	ATGCCGAGATTCTGCTACAGT
Pomc Rev	CCACACATCTATGGAGGTCTGAA
Igf1 (class II) For	CCTGGGTGTCCAAATGTAACTA
Igf1 (class II) Rev	TTTACACAGCAGGTCAGAGTG
Igfbp3 For	CCAGGAAACATCAGTGAGTCC
Igfbp3 Rev	GGATGGAACTTGGAATCGGTCA
Igfals For	CTGCCCGATAGCATCCCAG
Igfals Rev	GAAGCCAGACTTGGTGTGTGT
Gdf15 For	GAGAGGACTCGAACTCAGAAC
Gdf15 Rev	GACCCCAATCTCACCTCTG
36b4 For	AGATGCAGCAGATCCGCAT
36b4 Rev	GTTCTTGCCCATCAGCACC

Appendix Table S4. Information of human plasma samples used in Fig 6A.
There are 4 tabs in this file. Mean weight percentage is based on standard body weight chart with 50% as the median BW.

GDF15 (pg/ml)	Control	HD normal BW	HD FTT
mean	176	304	538
median	148	216	357
Standard deviation (SD)	108	300	560
Standard error of the mean (SEM)	16	52	84
% of samples outside of 2 SD	4.5%	5.9%	4.5%
t-test (control vs HD normal BW)	0.02		
t-test (control vs HD FTT)	0.00012		
t-test (HD normal BW vs HD FTT)	0.02		

Group 1: control

Sample #	Age (yrs)	Gender	% BW	Heart disease diagnosis
1	3	F	64.4%	None
2	3	M	62.5%	None
3	2	F	62.9%	None
4	3	M	64.1%	None
5	2	F	46.6%	None
6	2	M	52.8%	None
7	3	F	50.4%	None
8	3	F	68.9%	None
9	3	M	51.0%	None
10	3	F	68.3%	None
11	2	M	40.1%	None
12	2	F	42.9%	None
13	2	F	69.9%	None
14	2	F	62.3%	None
15	2	M	47.4%	None
16	2	F	66.3%	None
17	3	M	57.5%	None
18	2	M	68.8%	None
19	2	M	65.4%	None
20	2	F	58.4%	None
21	3	F	44.0%	None
22	3	M	53.8%	None
23	2	F	57.8%	None
24	3	F	50.0%	None
25	3	M	48.6%	None
26	2	F	65.0%	None
27	3	F	55.9%	None
28	3	F	68.0%	None
29	2	M	58.5%	None
30	3	M	61.6%	None
31	2	M	59.6%	None
32	3	M	45.9%	None
33	3	M	69.5%	None
34	2	M	61.6%	None
35	3	F	47.4%	None
36	3	M	46.2%	None
37	3	F	50.8%	None
38	2	M	62.9%	None
39	2	F	57.2%	None
40	3	M	44.4%	None
41	2	M	65.9%	None
42	2	F	54.9%	None
43	3	F	63.4%	None
44	3	M	52.8%	None
45	2	M	65.9%	None

Group 2: children with heart disease and normal body weight

Sample #	Age (yrs)	Gender	% BW	Heart disease diagnosis
46	2	M	69.7%	PDA, PFO, VSD
47	3	M	69.5%	TGV, DORV
48	3	F	69.3%	PPS
49	3	M	68.4%	ASD (Ostium secundum)
50	2	F	67.1%	Muscular VSD
51	2	F	66.8%	Congenital anomaly of aortic arch, PDA
52	3	F	62.1%	AVC
53	3	M	61.6%	Heart abnormality
54	3	M	59.0%	PPS
55	3	F	58.4%	Acyanotic congenital heart disease
56	2	F	57.1%	Congenital malposition of heart and cardiac apex, Mesocardia, Scimitar
57	3	M	56.8%	PPS
58	2	F	55.5%	PPS
59	3	M	55.3%	Complete common AVC, Other congenital ECD, Partial anomalous
60	3	F	55.2%	PPS
61	2	F	55.0%	ASD (Ostium secundum), VSD, Aortic arch hypoplasia
62	3	M	53.6%	Congenital anomaly of aortic arch, Congenital malposition of heart and
63	2	M	53.1%	Muscular VSD, ASD (Ostium secundum), PDA, PFO
64	3	M	53.1%	ASD (Ostium secundum), PDA
65	2	M	52.8%	Persistent left SVC
66	2	F	52.6%	ASD (Ostium secundum)
67	3	F	52.4%	PDA
68	2	M	52.4%	VSD
69	2	M	52.0%	HLHS, Pulmonary atresia, Right ventricular aorta with pulmonary atresia
70	3	F	51.6%	VSD (ventricular septal defect)
71	3	F	51.6%	VSD
72	2	M	51.1%	PPS
73	3	F	48.9%	ASD
74	2	F	48.5%	PDA
75	2	M	48.3%	PDA
76	3	F	44.5%	PFO
77	2	F	41.9%	PFO
78	3	F	41.6%	Congenital anomaly of aortic arch
79	3	F	40.9%	Other primary cardiomyopathies
80	3	F	40.2%	Anomalies of aortic arch, CoA (preductal, postductal), Congenital AVI

ASD: atrial septal defect
AVC: atrioventricular canal
AVI: aortic valve insufficiency
CoA: coarctation of the aorta
DORV: double outlet right ventricle
ECD: endocardial cushion defect
HLHS: hypoplastic left heart syndrome
PDA: patent ductus arteriosus
PFO: patent foramen ovale
PPS: peripheral pulmonic stenosis
PVS: pulmonary vein stenosis
SAS: subvalvar aortic stenosis
SVC: superior vena cava
TAPVC: total anomalous pulmonary venous connection
TAPVR: total anomalous pulmonary venous return
TGV: transposition of the great vessels
VSD: ventricular septal defect

Group 3: children with heart disease and FTT

Sample #	Age (yrs)	Gender	% BW	Heart disease diagnosis
81	2	M	8.4%	PFO
82	3	M	8.2%	PDA
83	2	M	8.0%	Congenital stenosis of pulmonary valve, Dysplastic pulmonary valve, PDA
84	3	F	7.9%	Congenital anomalies of pulmonary artery, Congenital malposition of heart and cardiac apex, Heart dextroposition, PDA
85	3	M	7.9%	ASD (Ostium secundum), VSD
86	2	M	7.8%	ASD (Ostium secundum), PDA, Other specified congenital anomaly of the heart
87	3	M	7.8%	TAPVC, TAPVR
88	2	M	7.2%	Bulbus cordis anomalies and anomalies of cardiac septal closure, Congenital mitral stenosis, Congenital stenosis of aortic valve, Congenital stenosis of pulmonary valve, HLHS, PVS, Other congenital anomalies of great veins, Other specified congenital anomaly of heart
89	3	M	6.6%	ASD
90	3	F	6.4%	PFO
91	3	F	6.1%	PFO
92	2	F	6.0%	CoA (preductal, postductal), Unspecified congenital anomaly of heart
93	3	M	5.9%	ASD (Ostium secundum), PFO
94	3	M	5.8%	Other primary cardiomyopathies
95	3	F	5.2%	ASD (Ostium secundum), PDA
96	3	M	5.0%	PDA
97	2	M	5.0%	PDA
98	2	F	4.8%	ASD (Ostium secundum), PFO
99	2	F	4.7%	ASD (Ostium secundum), PDA,VSD
100	3	M	4.6%	ASD (Ostium secundum)
101	2	M	3.8%	PDA
102	3	F	3.4%	PDA
103	3	F	3.4%	CoA (preductal, postductal), TGV, DORV, VSD, Other specified congenital anomaly of heart
104	2	F	3.3%	Complete common AVC, Congenital AVI, ECD, PDA, VSD
105	2	M	3.3%	ASD (Ostium secundum), Congenital malposition of heart and cardiac apex, Other congenital anomalies of pulmonary valve, PDA, Other specified congenital anomaly of heart
106	2	M	3.2%	ASD (Ostium secundum), VSD
107	3	F	2.8%	Congenital anomalies of pulmonary artery, ASD (Ostium secundum)
108	2	M	2.8%	Congenital anomaly of aortic arch
109	2	M	2.5%	TGV (D-loop, intact ventricular septum), ASD (Ostium secundum)
110	2	M	2.4%	Persistent left SVC, ASD (Ostium secundum), PFO
111	3	F	2.1%	Patent ductus arteriosus
112	2	F	1.5%	ASD (Ostium Secundum), PFO
113	3	M	1.5%	VSD
114	3	F	1.5%	Abnormality of aortic valve, Complete common AVC, Complete ECD, Congenital bilateral SVC, Congenital subaortic stenosis, SAS
115	2	F	1.3%	Congenital anomalies of pulmonary artery
116	2	M	1.3%	Muscular VSD, PDA
117	2	F	1.1%	PDA
118	2	F	1.0%	PDA, PFO
119	3	F	1.0%	ASD (Ostium secundum), Other congenital anomalies of great veins, Unspecified congenital anomaly of heart

120	3	M	0.7%	VSD
121	3	M	0.5%	PDA
122	3	M	0.3%	ASD (Ostium Secundum)
123	2	M	0.3%	ASD (Ostium secundum), Common (single) atrium, PFO
124	2	F	0.3%	ASD (Ostium secundum), PDA, Persistent left SVC
125	3	F	0.2%	PDA

ASD: atrial septal defect
AVC: atrioventricular canal
AVI: aortic valve insufficiency
CoA: coarctation of the aorta
DORV: double outlet right ventricle
ECD: endocardial cushion defect
HLHS: hypoplastic left heart syndrome
PDA: patent ductus arteriosus
PFO: patent foramen ovale
PVS: pulmonary vein stenosis
SAS: subvalvar aortic stenosis
SVC: superior vena cava
TAPVC: total anomalous pulmonary venous connection
TAPVR: total anomalous pulmonary venous return
TGV: transposition of the great vessels
VSD: ventricular septal defect

Appendix Table S5. Statistical analysis information.

Figure	Groups	p value
1A	Body weight of αKOγKO vs αHetγWT or αHetγKO or αWTγKO (biggest p value presented)	p = 0.02 (d5), 0.003 (d6), 0.016 (d7), $8x10^{-5}$ (d8), $3x10^{-5}$ (d9), $3x10^{-6}$ (d10), $2x10^{-7}$ (d11), $9x10^{-8}$ (d12), $2x10^{-8}$ (d13), $3x10^{-9}$ (d14), $3x10^{-9}$ (d15), $3x10^{-7}$ (d16)
1C	Plasma IGF1 of P10 αKOγKO vs αHetγWT or αHetγKO or αWTγKO (biggest p value presented)	p = 0.04
1D	Plasma IGF1 of P16 αKOγKO vs αHetγWT or αHetγKO or αWTγKO (biggest p value presented)	p = 0.04
1F	Liver p-STAT5 between αKOγKO and αHetγWT	p = 0.0013
1G	Liver gene expression in αKOγKO vs αHetγWT or αHetγKO or αWTγKO (biggest p value presented)	p = 0.011 for Igf1, p = 0.0015 for Igfbp3, p = 0.006 for Igfals
1H	Liver/plasma IGFBP3 between αKOγKO and αHetγWT	p = 0.000009 for liver IGFBP3, p = 0.000007 for plasma IGFBP3
3A	Plasma IGF1 between GDF15 and PBS injected mice	p = 0.004
3C	Liver gene expression between GDF15 and PBS injected mice	p = 0.004 for Igf1, p = 0.02 for Igfbp3, p = 0.0013 for Igfals
3D	Plasma IGF1 between GDF15 and PBS injected mice	p = 0.006
3E	Plasma IGFBP3 between GDF15 and PBS injected mice	p = 0.04
3G	Body weight between GDF15 and PBS injected mice	p = 0.03 (d8), 0.004 (d9), 0.0008 (d10), 0.003 (d11)
4A	Cardiac Gdf15 mRNA level between αKOγKO and αHetγWT	p = 0.007 (P7), 0.00016 (P10), 0.0009 (P13)
4C	Plasma GDF15 between αKOγKO and αHetγWT	p = 0.02 (P7), 0.0012 (P10)
5B	Cardiac Gdf15 mRNA in AAV-shRNA injected mice	p = 0.004 (αHetγWT shControl vs αKOγKO shControl), p = 0.006 (αKOγKO shControl vs αKOγKO shGdf15)
5C	Plasma GDF15 in AAV-shRNA injected mice	p = 0.02 (αHetγWT shControl vs αKOγKO shControl), p = 0.04 (αKOγKO shControl vs αKOγKO shGdf15)
5D	Cardiac Bnp mRNA in AAV-shRNA injected mice	p = 0.0011 (αHetγWT shControl vs αKOγKO shControl), p = 0.0008 (αHetγWT shControl vs αKOγKO shGdf15)
5E	Liver p-STAT5 in AAV-shRNA injected mice	p = 0.0009 (αHetγWT shControl vs αKOγKO shControl), p = 0.03 (αKOγKO shControl vs αKOγKO shGdf15)
5F	Plasma IGF1 in AAV-shRNA injected mice	p = 0.0005 (αHetγWT shControl vs αKOγKO shControl), p = 0.03 (αKOγKO shControl vs αKOγKO shGdf15), p = 0.0019 (αHetγWT shControl vs αKOγKO shGdf15)
6A	Plasma GDF15 in children	p = 0.02 (control vs HD normal BW), p = 0.00012 (control vs HD FTT), p = 0.02 (HD normal BW vs HD FTT)
