# Supplementary figures and images for: GDF15 is a heart‐derived hormone that regulates body growth
Source: EMBO Mol Med. 2017 Jun 1;9(8):1150–64. doi: 10.15252/emmm.201707604 (PMC5538424; doi:10.15252/emmm.201707604)

Figure 1E

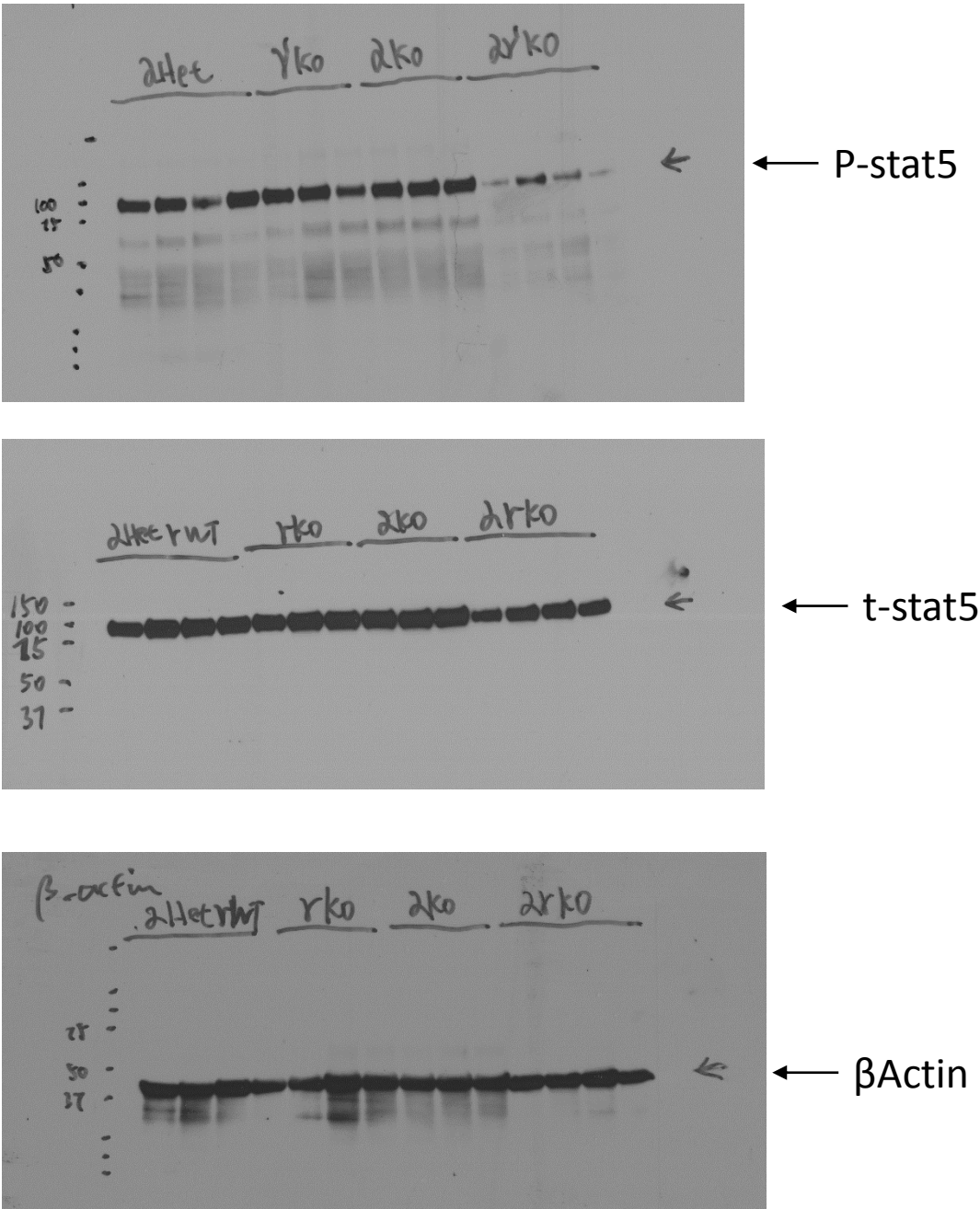

Supplement: Supplementary file 3 — Source Data for Figure 1 [file EMMM-9-1150-s002.pdf]

Figure 2A

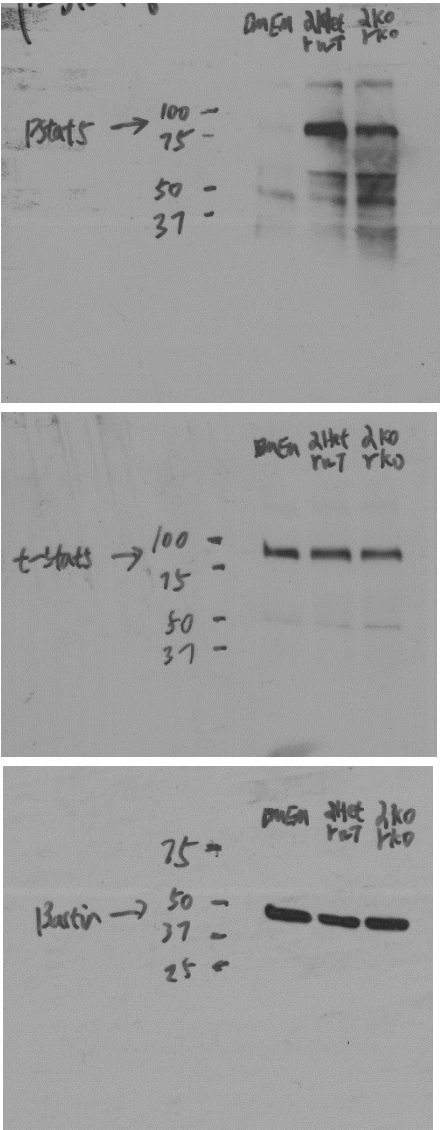

Figure 2B

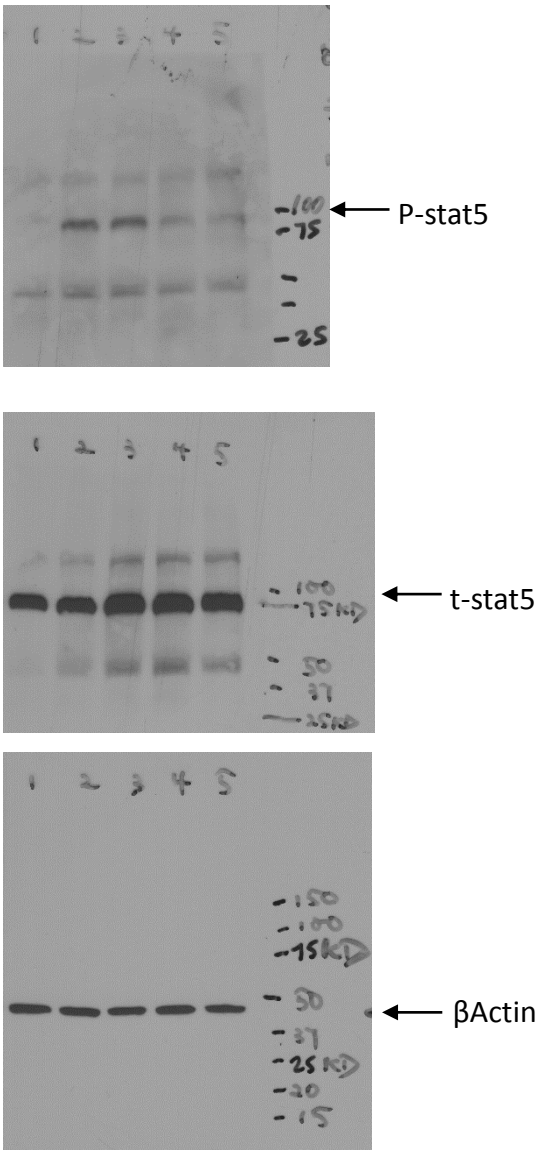

Supplement: Supplementary file 4 — Source Data for Figure 2 [file EMMM-9-1150-s003.pdf]

Figure 3B

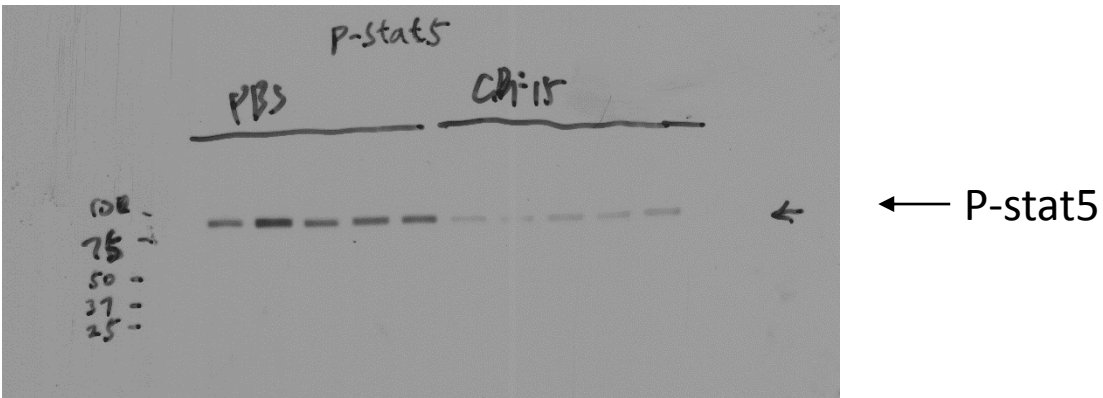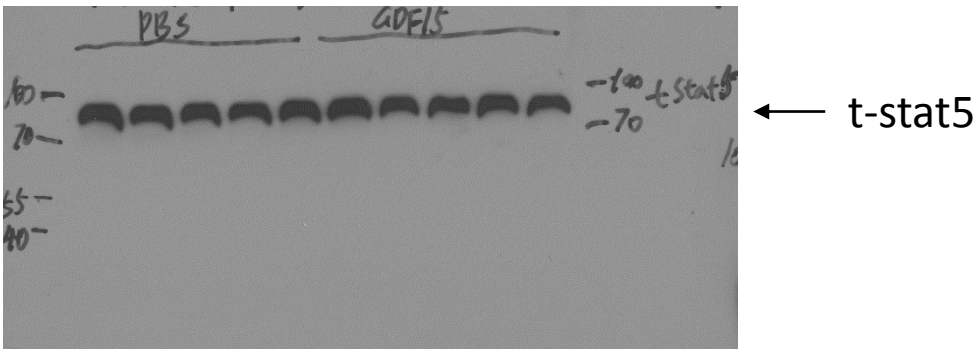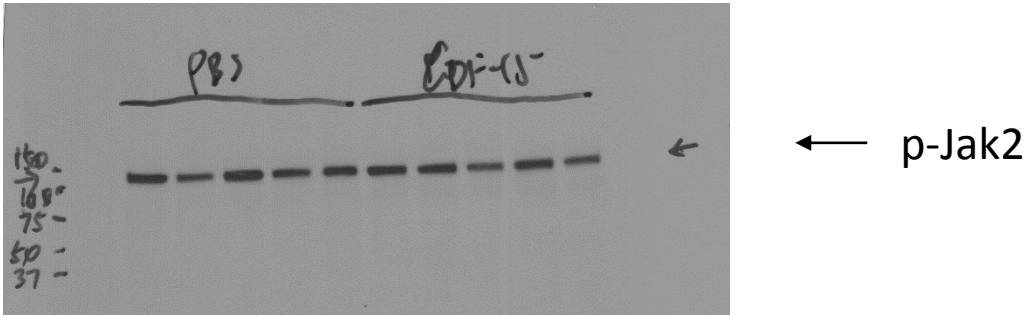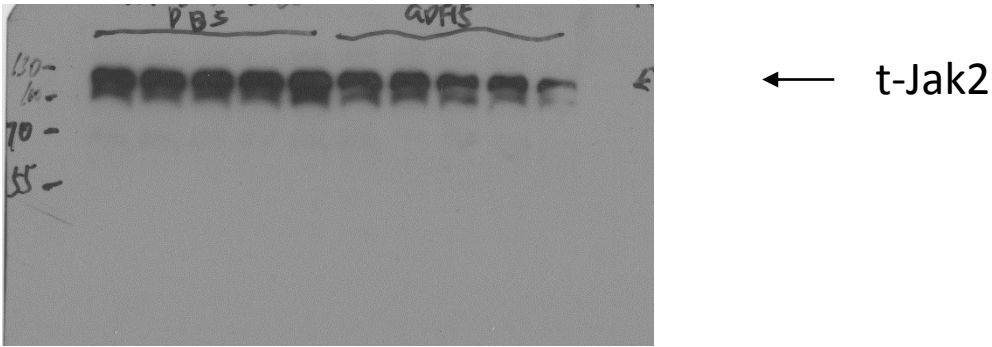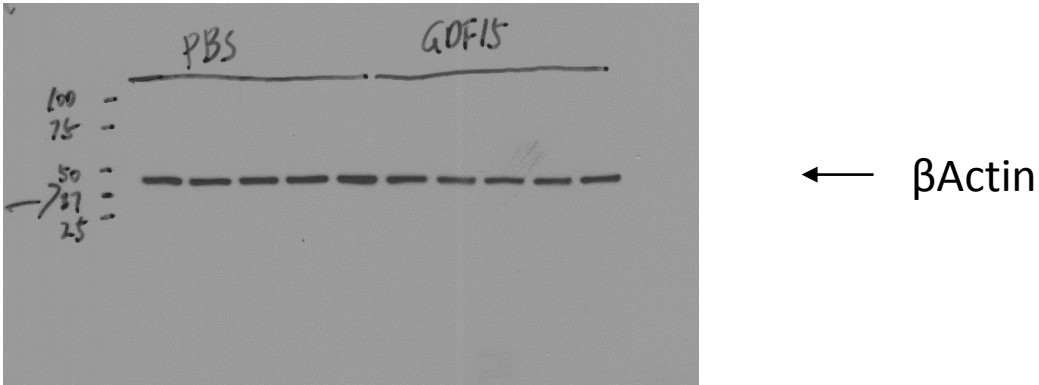

Supplement: Supplementary file 5 — Source Data for Figure 3 [file EMMM-9-1150-s004.pdf]
